# Exploring the therapeutic potential of triterpenoid saponins from *Gymnema sylvestre*: Mechanistic insights into hepatoprotection, immunomodulation, anticancer activities, molecular docking, and pharmacokinetics

**DOI:** 10.1016/j.heliyon.2024.e40850

**Published:** 2024-11-30

**Authors:** Vasudeva Reddy Netala, Tianyu Hou, Rajakumari Devarapogu, Murali Satyanarayana Bethu, Zhijun Zhang, Tartte Vijaya

**Affiliations:** a Department of Biotechnology, Sri Venkateswara University, Tirupati, A.P, 517502, India; b School of Chemical Engineering and Technology, North University of China, Taiyuan, Shanxi, 030051, China; c Department of Zoology, Sri Venkateswara University, Tirupati, A.P, 517502, India; d Department of Toxicology and Cancer Biology, CSIR-Indian Institute of Chemical Technology, Hyderabad, Telangana, 500007, India; e Department of Botany, Sri Venkateswara University, Tirupati, A.P, 517502, India

**Keywords:** *Gymnema sylvestre*, Triterpenoid saponin extract, Hepatoprotective, Antioxidant, Immunomodulatory, Anticancer, TNF-α inhibition, HER-2 inhibition

## Abstract

The study comprehensively investigated the therapeutic potential of *Gymnema sylvestre* triterpenoid saponin extract (GST), encompassing its hepatoprotective, immunomodulatory, and anticancer activities. The study employed a Prednisolone (PRD)-induced immunosuppressed rat model to assess the hepatoprotective and immunomodulatory effects of GST. Using this model, GST was found to modulate haematopoiesis, improving RBC, platelet, and WBC counts, underscoring its potential in hematopoietic homeostasis. Organ atrophy, a hallmark of immunosuppression in spleen, thymus, liver, and kidneys, was reversed with GST treatment, reinforcing its hepatotrophic and organotropic capabilities. Elevated hepatic biomarkers, including alanine transaminase (ALT), aspartate transaminase (AST), alkaline phosphatase (ALP), and lipid peroxidase (LPO), indicative of hepatocellular injury and oxidative stress, were reduced with GST, underscoring its hepatoprotective and antioxidative effects. Additionally, GST restored depleted antioxidants glutathione (GSH) and superoxide dismutase (SOD), highlighting its strong antioxidative capabilities. Molecular insights revealed a downregulation of interleukin-2 (IL-2) and interleukin-4 (IL-4) mRNA in the spleen of immunosuppressed rats, while GST treatment significantly upregulated IL-2 and IL-4 mRNA expression, showcasing its immunomodulatory potential. Increased levels of tumor necrosis factor-α (TNF-α) associated with immune dysregulation were effectively decreased by GST, underscoring its role in modulating inflammatory responses and restoring immune balance. Molecular docking studies indicated strong inhibition of TNF-α by GST compounds. In terms of anticancer activity, GST demonstrated significant cytotoxicity against MCF-7, and MDA-MB-231 (breast cancer cell lines). Notably, GST demonstrated biocompatibility with normal CHO (Chinese hamster ovary cell line) and HUVEC (Human umbilical vein endothelial cells) cell lines. Molecular docking studies indicated strong inhibition of breast cancer proteins HER1 and HER2 (human epidermal growth factor receptors) by GST compounds. Additionally, pharmacokinetics, bioavailability, drug-likeness, and toxicity risk predictions suggest that GST compounds are pharmacologically favourable with no adverse effects.

## Highlights

• Triterpenoid saponins exhibited strong hepatoprotective and immunomodulatory properties.
• Triterpenoid saponins exhibit potent cytotoxicity against breast cancer cells and show biocompatibility towards normal cells.
• Triterpenoid saponins increase IL-2 and IL-4 expression levels while suppressing TNF-α expression levels.
• Triterpenoid saponins are found to be effective inhibitors of TNF-α, EGFR, and HER-2, as validated by molecular docking studies.
• Triterpenoid saponins meet pharmacokinetic profiles, bioavailability, ADMET properties, and drug-likeness parameters, as confirmed by *in silico* approaches.

## Graphical abstract

**Figure:**
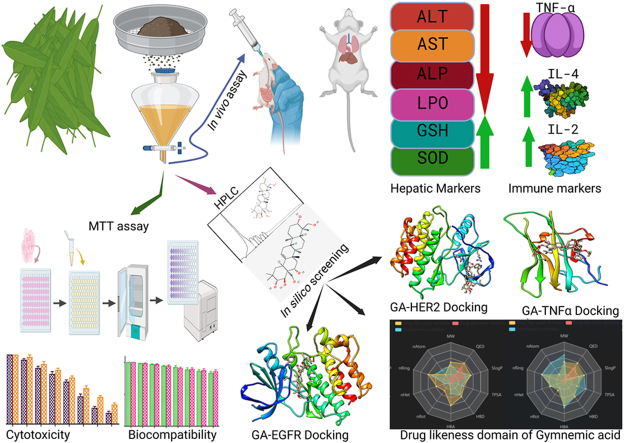

## 1 Introduction

The immune system is a complex defense mechanism evolved to protect organisms from pathogens and diseases. It involves a network of specialized cells and molecules that recognize and neutralize foreign antigens, comparable in complexity to the nervous system [1]. Effective immune responses rely on antigen presentation, B- and T-cell activation, and the release of antibodies and cytokines [2]. The immune system's function can be modulated by various factors, resulting in either immune enhancement or suppression. Agents that modify these processes are known as immunomodulators [3]. Recently, there has been a growing interest in using natural therapeutic agents due to their safety and efficacy. With increasing concerns about adverse effects of synthetic drugs, herbal immunomodulators are being explored as alternative treatments for various diseases [4]. These remedies are believed to strengthen the body's innate resistance, and their immunomodulatory properties have been confirmed across many plant species. The search for new bioactive compounds with immunomodulatory potential from plants has become a significant area of modern research [5].

Plant-based treatments with immunomodulatory properties are gaining attention from scientists. Using advanced technology and research on natural products like plants and their extracts can help find new immunomodulatory agents. These agents could enhance current cancer treatments [6]. Triterpenoid saponins are bioactive compounds renowned for their capacity to enhance antibody production and modulate both T-cell-dependent and T-cell-independent immune responses [7]. Triterpenoid saponins exert robust immunomodulatory effects, including the potentiation of cytotoxic CD8^+^ lymphocyte responses and enhancement of mucosal antigen reactivity [8]. Moreover, triterpenoid saponins exhibit a dual modulatory role, stimulating specific immune components while also eliciting non-specific immune reactions such as inflammation and monocyte proliferation [9]. However, the molecular mechanisms underlying their activity are not fully understood. Studies have highlighted the immunomodulatory potential of triterpenoid saponins from various plant sources. For instance: Triterpenoid saponins from the rhizomes of *Anemone raddeana* enhanced both humoral and cellular immune responses in mice [10]. Triterpenoid saponins from *Panax ginseng* increased natural killer (NK) cell activity and improved phagocytic functions, enhancing the immune response to foot and mouth disease vaccination in mice [11]. Triterpenoid saponins from *Moringa oleifera* leaves exhibited antioxidant and immunomodulatory activities [12]. Triterpenoid saponins from *Pulsatilla chinensis* roots acted as adjuvants in murine models [13]. Triterpenoids from *Mollugo verticillata* reduced cyclophosphamide-induced toxicity in mice [14]. Triterpenoid saponins from *Centella asiatica* roots showed hepatoprotective and immunomodulatory effects against cyclophosphamide-induced immunosuppression [15]. Thus, the plant-based immunomodulators, particularly triterpenoid saponins, show promising potential as alternative therapeutic agents for enhancing immune responses and treating various diseases.

Moreover, triterpenoid saponins have shown a broad spectrum of anticancer activities against various cancer cell lines, as demonstrated by their ability to induce cell cycle arrest, trigger apoptosis, and inhibit proliferation in different cancer types [6]. These compounds have the potential to act as potent inhibitors of tumor growth and metastasis, thereby improving patient outcomes and quality of life [16]. Triterpenoid saponins have shown promise in both modulating immune responses and inhibiting cancer cell proliferation, making them attractive candidates for the development of novel anticancer agents [17]. Leveraging the immune-enhancing capabilities of these compounds could lead to innovative therapeutic strategies. By harnessing the body's immune system to target cancer cells, these compounds may offer a more holistic approach to cancer treatment, potentially enhancing the efficacy of current cancer therapies [18].

*G. sylvestre* R. Br., indigenous to India, South Asia, and Africa, is a vine belonging to the Asclepiadaceae family and is widely recognized by its common name, Gur-mar (sugar destroyer). With a history spanning over two millennia, this plant's leaves have been integral to traditional Indian and Chinese medicinal practices [19,20] Traditionally consumed as a tea or by directly chewing its leaves, *G. sylvestre* has seamlessly transitioned into modern dietary regimens, being available in powder, pill, and tablet forms [21]. *G. sylvestre* has garnered significant attention for its potent antidiabetic properties and has been incorporated into functional foods aimed at managing blood sugar levels, promoting weight loss, and supporting cardiovascular health [22]. In addition to its antidiabetic prowess, *G. sylvestre* leaves exhibit a spectrum of bioactive attributes, including hypolipidemic, anti-inflammatory, antioxidant, and antimicrobial activities [[19], [20], [21], [22]]. These multifaceted therapeutic effects are primarily attributed to the triterpenoid saponins present in the leaves, with gymnemic acid representing the collective term for this diverse group of compounds. The leaves of *G. sylvestre* are known to harbor approximately 18 distinct gymnemic acids, from GA I to GA XIV, each characterized by gymnemagenin as their sapogenin backbone and embellished with unique sugar moieties [23].

In recent years, *G. sylvestre* has expanded its influence beyond traditional medicinal applications, establishing a significant presence in the herbal and pharmaceutical sectors. Acknowledged as a natural reservoir of potent bioactive compounds, this plant has distinguished itself as a valuable functional ingredient in a diverse range of herbal formulations [[24], [25], [26], [27]]. This prominence aligns with the escalating consumer demand for natural and health-enhancing additives. Concurrently, the pharmaceutical industry has recognized the therapeutic potential of *G. sylvestre*, positioning it as a promising candidate for the development of innovative therapeutic agents targeting a spectrum of health conditions, including diabetes, liver disorders, inflammation, and microbial infections [[24], [25], [26], [27]].

Despite existing research highlighting the immunomodulatory potential of triterpenoid saponins from various botanical sources, the in vivo immunomodulatory and anticancer activities of *G. sylvestre* triterpenoid saponins remain relatively unexplored. This presents a compelling research gap and an opportunity for innovative investigation. Consequently, the present study endeavors to elucidate the immunomodulatory and anticancer properties of *G. sylvestre* triterpenoid saponin extract.

To achieve this objective, a comprehensive evaluation of the hepatoprotective, immunomodulatory, and anticancer efficacies of these triterpenoid saponins will be undertaken. This rigorous assessment will encompass a multidimensional approach, integrating analyses of oxidative stress markers, hematological parameters, relative organ weights, gene expression profiles of immune markers, cell viability assay, and molecular docking studies. Additionally, biocompatibility, bioavailability, drug-likeness, and toxicity evaluations were meticulously conducted to provide a comprehensive understanding of the compound's pharmacological profile using *in silico* approaches. This systematic and integrative research initiative aims to illuminate the therapeutic potential of *G. sylvestre* triterpenoid saponins, bridging the knowledge gap between traditional medicinal wisdom and contemporary scientific validation. Furthermore, this study seeks to elucidate the promising role of these bioactive compounds in oncological therapeutics, thereby paving the way for the exploration and exploitation of *G. sylvestre* multifaceted health benefits in modern medical applications.

## 2 Materials and methods

### 2.1 Chemicals and instruments

Prednisolone, levamisole hydrochloride, and all other chemicals utilized in this study were procured from reputable suppliers including Sigma Aldrich, Himedia, Thermo Fisher Scientific, and Merck India. Centrifugation was performed using a REMI cooling microfuge (Model: CM-12, India). Optical density (OD) values were recorded using an Ultraviolet–Visible (UV–Vis) double-beam spectrophotometer (Analytical Technologies Limited, India). High-quality Milli-Q water (Merck Millipore, USA) was employed to ensure accurate and reliable results.

### 2.2 Cell lines and cell culture

All cancer cell lines, including COLO205 (Human colon cancer), PC3 (Human prostate carcinoma), SKOV3 (Human ovarian carcinoma), B16F10 (Mouse melanoma), MCF-7, and MDA-MB-231 as well as the normal CHO and HUVEC cell lines, were obtained from the National Centre for Cell Science (NCCS) in Pune, India. Cells were cultured in either RPMI (Roswell Park Memorial Institute) 1640 medium (for PC3) or Dulbecco's Modified Eagle's Medium (DMEM) (COLO205, SKOV3, B16F10, MCF-7, MDA-MB-231). The culture media were supplemented with 10 % (v/v) heat-inactivated fetal bovine serum (FBS), 1 mM NaHCO3, 2 mM L-glutamine, 100 units/mL penicillin, and 100 μg/mL streptomycin. All cell lines were maintained in culture at 37 °C in a humidified atmosphere containing 5 % CO2. The cell lines used in this study were purchased from the National Centre for Cell Science (NCCS). The NCCS conducted rigorous authentication procedures, including genetic profiling and identity verification, before distributing cell lines. The NCCS also performed mycoplasma testing to ensure that the cell lines are free from contamination. Only after passing these tests are the cell lines provided to researchers.

### 2.3 Preparation of GST

Fresh and healthy leaves of *G. sylvestre* were harvested from Tirumala hills, located in the Eastern Ghats of Andhra Pradesh, India. The plant specimen was authenticated by a qualified taxonomist at Sri Venkateswara University, Tirupati, India, and voucher specimen number (Ref. No. SVUTYGS-01-05) has been archived at the Herbarium Centre, Department of Botany, Sri Venkateswara University, Tirupati, India. Freshly harvested leaves were washed thoroughly with distilled water to remove dust and other impurities. The leaves were then air-dried in the shade at room temperature for 7 days to prevent degradation of active compounds. Once completely dried, the leaves were ground into a fine powder using an electric grinder and stored in an airtight container at room temperature. *G. sylvestre* leaf powder was defatted using petroleum ether for 3–4 h. After defatting, the extracted material was combined with 5 vol of methanol and subjected to refluxing at 50 °C for 4 h, followed by concentration. The concentrated extract was then refluxed again with an additional 5 vol of methanol at 50 °C for 2 h under vacuum conditions. Then the extract was treated with methanol:acetone solution (1:5 ratio) for about 4 h. This process is repeated twice to obtain the saponin precipitate. Then this precipitate was dried under vacuum and the dried precipitate was purified via C-18 silica gel column using chloroform and methanol (7:3). The purified extract was concentrated using a rotary evaporator at 50 °C under vacuum to remove excess solvent and yield a thick, semi-solid residue which was named as *G. sylvestre* triterpenoid saponin extract (GST) [28,29]. GST was stored in amber glass bottles at 4 °C to prevent light and moisture exposure, ensuring the stability of the active compounds until further use.

### 2.4 High-performance liquid chromatography (HPLC) analysis of GST

One gram of GST was initially treated with 4 mL of 12 % potassium hydroxide (KOH) and refluxed for 1 h. Subsequently, 10 mL of 4 N hydrochloric acid (HCl) was added to the extract and refluxed for an additional 1 h. This acid:base treatment was employed to release gymnemagenin, backbone of gymnemic acids which is regarded as standard for the detection and quantification of triterpenoid saponins of *G. sylvestre.* After refluxing, this solution was allowed to cool and then made up to 50 mL with methanol. Then it was filtered using Millipore filter paper. To ensure consistency across batches, GST was standardized based on the concentration of gymnemagenin, a key bioactive compound. HPLC was employed for this purpose using acetonitrile and methanol (80:20) as the mobile phase, with a flow rate set at 1 mL/min. The column temperature was maintained between 25 and 28 °C [28,29]. Pure gymnemagenin was obtained from Natural remedies Bangalore, India and used as reference standard.

### 2.5 Experimental animals and housing conditions

Male Wistar strain albino rats, weighing between 150 and 175 gm, were sourced from Sri Venkateswara Animal Agency, Bangalore, India. These rats were housed and acclimatized in polypropylene cages within the Department's animal facility under controlled conditions: a temperature of 24 ± 2 °C, relative humidity ranging from 44 to 56 %, and a photoperiod of 10 h light and 14 h dark. The acclimatization period lasted one week prior to the commencement of the experiment and was maintained throughout the study duration. Rats had unrestricted access to standard rodent pellet chow (Sri Sai Durga Animal Feeds, Bangalore, India) and water ad libitum. All experimental procedures were conducted in accordance with the guidelines set by the Committee for the Purpose of Control and Supervision of Experiments on Animals (CPCSEA), India. The study protocol was approved by the Institutional Animal Ethical Committee (IAEC) at the Department of Zoology, Sri Venkateswara University, Tirupati, India (IAEC/No.438/(i)/(a)/CPCSEA). For this study, a dosage of 5 mg of prednisolone per kg body weight was administered based on established literature [30]. Similarly, a dosage of 10 mg of levamisole per kg body weight was chosen based on existing literature [31]. Based on acute toxicity studies (Table 1S), 600 mg/kg of GST was identified as the dose at which mild toxicity symptoms were observed, while 1000 mg/kg of GST led to mortality. Therefore, the lethal dose (LD50) was estimated to be between 600 mg/kg and 1000 mg/kg of GST. For experimental purposes, a safer dose of 150 mg/kg body weight, equivalent to one-fourth of the 600 mg/kg dose, was selected to minimize toxicity risks during further studies (Table 1S supplementary file)

**Table 1:** Effect of triterpenoid saponin extract of *G. sylvestre* on hematological profile in control and experimental animals.

Parameters	CON	GST	PRD	PRD + GST	PRD + LEV
Haemoglobin (gm/dL)	14.78 ± 0.32^a^	14.93 ± 0.27^a^	12.02 ± 0.36^b^	13.86 ± 0.41^c^	14.61 ± 0.33^c^
Red Blood Cells (10^6^/μL)	7.37 ± 0.20^a^	7.42 ± 0.18^a^	6.03 ± 0.38^b^	7.14 ± 0.23^c^	7.31 ± 0.34^c^
White Blood Cells (10^3^/μL)	9.38 ± 0.26^a^	9.87 ± 0.23^a^	7.15 ± 0.36^b^	12.91 ± 0.26^c^	13.16 ± 0.33^c^
Platelets (10^5^/μL)	6.61 ± 0.28^a^	6.56 ± 0.31^a^	5.06 ± 0.24^b^	6.17 ± 0.33^c^	6.43 ± 0.29^c^
% Neutrophils	23.80 ± 1.26^a^	23.45 ± 0.91^a^	22.71 ± 0.77^a^	23.16 ± 0.58^a^	22.66 ± 0.78^a^
% Lymphocytes	73.33 ± 1.49^a^	74.18 ± 1.07^a^	72.33 ± 1.29^a^	73.15 ± 1.14^a^	73.41 ± 1.31^a^
% Monocytes	2.32 ± 0.18^a^	2.25 ± 0.15^a^	2.16 ± 0.23^a^	2.32 ± 0.21^a^	2.09 ± 0.19^a^
% Eosinophils	1.64 ± 0.14^a^	1.25 ± 0.16^a^	3.01 ± 0.18^b^	1.94 ± 0.15^a^	2.02 ± 0.16^a^
% Basophils	0.23 ± 0.06^a^	0.18 ± 0.05^a^	0.51 ± 0.08^a^	0.43 ± 0.07^a^	0.38 ± 0.08^a^

**CON**: Control; **GST**: Triterpenoid saponin extract; **PRD**: Prednisolone.

**PRD + GST**: Prednisolone + Extract; **PRD + LEV**: Prednisolone + Levamisole.

Data represent mean ± standard deviation (SD) of 6 individual rats.

Values are not sharing a common superscript (a,b,c) differ significantly at P ≤ 0.05 (DMRT).

### 2.6 Experimental design

Thirty rats were divided into five experimental groups, with each group consisting of six rats (n = 6). Group I: CON; Normal Control Rats (n = 6): These rats received standard pellet chow and water for a duration of 30 days. Group II: GST; Extract-Treated Rats (n = 6): These rats were administered GST at a dosage of 150 mg/kg body weight via gavage. They also received standard pellet chow and water for 30 days. Group III: PRD; Immunosuppressed Rats (n = 6): These rats were given PRD at a dosage of 5 mg/kg body weight via gavage, along with standard pellet chow and water for 30 days. Group IV: PRD + GST; Immunosuppression-Treated Rats (n = 6): These rats received both PRD (5 mg/kg body weight) and GST (150 mg/kg body weight) via gavage, in addition to standard pellet chow and water for 30 days. Group V: PRD + LEV; Immunosuppression-Treated Rats (n = 6): These rats were administered PRD (5 mg/kg body weight) and levamisole (LEV) (10 mg/kg body weight) via gavage, along with standard pellet chow and water for 30 days. All rats underwent an overnight fasting period but had free access to water on the final day of drug administration. For euthanasia, the rats were anesthetized using xylazine (95 mg/kg body weight) and ketamine (85 mg/kg body weight). Subsequently, selective organs such as the spleen, thymus, liver, and kidneys were dissected out for further analysis.

### 2.7 Determination of hematological parameters and organ weights

Blood samples were collected via retro-orbital plexus puncture from all experimental animals for hematological analysis. The obtained samples were subjected to quantification of the following parameters using an automated hematology analyzer: (a) Red Blood Cell (RBC) count; (b) Total White Blood Cell (WBC) count; (c) Differential White Blood Cell count and (d) Hemoglobin concentration. Before euthanasia, the body weights of the animals were recorded. Subsequent to euthanasia, the spleen, thymus, liver, and kidneys were excised, weighed, and the results were expressed as relative organ weights.

### 2.8 Estimation of oxidative stress markers and biochemical parameters

Liver tissue homogenates were prepared using ice-cold Tris buffer (0.1 M, pH 7.4) and centrifuged at 4 °C at 1500 rpm for 30 min. The resulting supernatant was utilized for the quantification of various oxidative stress markers and biochemical parameters, as follows: LPO was assessed by measuring MDA levels, according to the method described by Ohkawa et al. [32]. GSH levels were determined using the method established by Moron et al. [33]. ALT activity was evaluated using the Reitman and Frankel method [34]. AST or Glutamate Oxaloacetate Transaminase (GOT) levels were quantified following the procedure outlined by Huang et al. [35]. ALP levels were estimated based on the King method [36]. SOD activity was determined according to the method described by McCord and Fridovich [37].

### 2.9 mRNA expression levels of cytokines (IL-2 and IL-4) by RT-PCR

RNA isolation was performed using the TRIzol method. Experimental spleen tissue samples (60–80 mg) were flash-frozen in liquid nitrogen and subsequently ground to a fine powder using a mortar and pestle. RNA extraction from the spleen tissue samples was conducted utilizing TRIzol reagent (Invitrogen). The isolated RNA pellet was washed with 1 mL of 75 % ethanol, vortexed, and centrifuged at 8000 rpm for 5 min at 2 °C. The resulting RNA pellet was air-dried for 10 min and then reconstituted in DEPC (diethyl pyrocarbonate)-treated water. The concentration and purity of the RNA were assessed by measuring the optical density at A260 and A280 using a NanoDrop spectrophotometer. The concentration of total RNA (μg/mL) was calculated using the formula: Concentration = (OD260) × 40.

cDNA synthesis was carried out using the SuperScript® III First-Strand cDNA Synthesis Kit (Invitrogen). The cDNA synthesis reaction included 3 μg of template RNA, 3 μL of Oligo (dT)18 primer (50 mM), and nuclease-free water to achieve a total volume of 12 μL. The synthesized cDNA served as a template for the subsequent amplification of IL-2, IL-4, and β-actin (housekeeping gene) using specific primers [38]. The primer sequences were as follows: **IL-2:** 5′-ATGTACAGCATGCAGCTCGCAT-3′ and 5′-TCATTGTTGAGATGATGCTTTGACA -3’; **IL-4:** 5′-ATGGGTCTCAACCCCCACCTTG C -3′ and 5′-GACTAACCTCAGCCTCCACGAAGTA-3′ TNF-α: 5′- ATGAGCACGGAA AGCATGATCCGA-3′ and 5′-CCAAAGTAGACCTGCCCGGACTC-3′, **β-actin:** 5′-ATGGATGACGATATCGCTG-3′ and 5′-ATGAGGTAGTCTGTCACGT-3’. PCR cycle was conducted in a 50 μL reaction mixture containing: 5 μL of cDNA (50 ng/μL), 1 μL of dNTPs (10 mM), 1 μL of each forward and reverse primer (200 ng/μL), 5 μL of 10X PCR buffer, 1 μL of Taq DNA Polymerase, and 36 μL of nuclease-free water. The PCR cycling conditions were as follows: initial denaturation at 94 °C for 5 min, followed by 35 cycles of denaturation at 94 °C for 1 min, annealing at 58 °C for 1 min, extension at 72 °C for 1 min, and a final extension at 72 °C for 7 min. The amplified PCR products were separated by 1.5 % agarose gel electrophoresis, visualized by ethidium bromide staining using GelDoc (Major Scientifics, India), and captured with a digital camera. Band intensities were quantified using ImageJ software and normalized to β-actin expression levels.

### 2.10 Determination of anticancer activity by MTT assay

The anticancer activity of the GST was evaluated using the MTT assay, as per the method described by Mossman [[39], [40], [41]]. Cells (5 x 10^3) were seeded in each well of 96-well plates, each containing 100 μL of growth medium. Plates were incubated overnight at 37 °C in a humidified atmosphere with 5 % CO2. After incubation, 100 μL of medium containing varying concentrations of GST (25, 50, 75, 100, 150, 200, and 250 μg/mL) was added to the respective wells. The viability of cells was assessed after 24 h by adding 10 μL of MTT solution (5 mg/mL) to each well. The plates were then incubated for an additional 3 h at 37 °C. After the incubation period, the medium was carefully discarded, and the formazan crystals formed in the cells were dissolved in 100 μL of dimethyl sulfoxide (DMSO). The intensity of the formazan blue color was measured at 570 nm using a spectrophotometer (Spectra MAX Plus; Molecular Devices) equipped with SOFTmax PRO-5.4 software. The percentage inhibition of cell viability was calculated relative to the control values (without GST). The IC50 concentrations (the concentration causing a 50 % decrease in cell viability compared to control values) were determined using the respective regression equations.

### 2.11 Molecular docking analysis

#### 2.11.1 Retrieval of ligands and proteins

The 3D structures of triterpenoid saponins from Gymnema sylvestre, including Gymnemagenin and Gymnemic acid I to Gymnemic acid XIV, were sourced from the PubChem database. Crystal structures for TNF-α (RCSB PDB ID: 2AZ5), HER1 (RCSB PDB ID: 4HJO) and HER2 (RCSB PDB ID: 3PP0) were obtained from the Protein Data Bank (https://www.rcsb.org/).

#### 2.11.2 Virtual screening and docking

Ligand-based virtual screening was conducted using AutoDock Vina 4.05 through PyRx 0.51. Initially, all ligand molecules were uploaded and subjected to energy minimization using the universal force field with a conjugate-gradient algorithm set to 200 iterations. Virtual screening against the HER1 and HER2 proteins was performed using the Lamarckian genetic algorithm. The docking parameters were configured as follows: population size of 150 individuals, maximum energy evaluations set at 25,000, maximum number of generations at 27,000, top individual survival rate at 1, gene mutation rate of 0.02, crossover rate of 0.8, Cauchy beta value at 1.0, and a genetic algorithm window size of 10.0. The grid for docking was centered on the binding pocket coordinates X = 29.3901, Y = −42.745, Z = −51.82, with dimensions (Å) X = 90.000, Y = 105.7097, Z = 104.2448, and exhaustiveness set to 8. The most favourable docked ligand conformations were saved for further analysis, including bond angles, bond lengths, and hydrogen bonding interactions, which were examined using PyMOL.

### 2.12 Drug-likeness, pharmacokinetics, bioavailability and ADMET predictions

Physicochemical descriptors, Lipinski's Rule of Five, Solubility, Lipophilicity, Pharmacokinetics, drug-likeness, and medicinal chemistry predictions were carried out by using SwissAdme tool (http://www.swissadme.ch) Additionally, Absorption, Distribution, Metabolism, Excretion, and Toxicity (ADMET) properties, were predicted using admetSAR 3.0 (http://lmmd.ecust.edu.cn/admetsar3/about.php) tool. The bioactivity scores (nuclear receptor ligand, GPCR ligand, Protease inhibitor Enzyme inhibitor, Kinase inhibitor, and Ion channel modulator property) of triterpenoid saponins were analyzed using the Molinspiration software platform (https://molinspiration.com/).

### 2.13 Statistical analysis

All experiments were conducted in triplicate, and data are expressed as the mean ± standard deviation (SD). Statistical significance was evaluated using appropriate tests based on the type of data collected. The following statistical methods were employed [42].

When more than two groups were compared, such as in dose-dependent cytotoxicity studies or multiple time-point evaluations, we employed a one-way analysis of variance (ANOVA) followed by Tukey's post-hoc test. This allowed us to determine significant differences between different treatment groups.

IC_50_ values (the concentration required to inhibit 50 % of cell viability) were calculated using non-linear regression analysis with GraphPad Prism software. The fitted curve and confidence intervals were used to assess the potency of the extracts.

A p-value of less than 0.05 (p < 0.05) was considered statistically significant. P-values are indicated in the reledert tables and figures to show the statistical significance of the results.

The data were graphically represented using bar charts, and error bars indicate standard deviations. Detailed statistical output, including p-values, sample sizes (n), and test statistics, are provided for each figure and table.

## 3 Results and discussion

The current study aims to investigate the hepatoprotective, immunomodulatory and anticancer activities of the triterpenoid saponin extract from *G. sylvestre* (GST). Hepatoprotective and immunomodulatory properties were studied by employing prednisolone (PRD)-induced immunosuppressed rats. The immunomodulatory effects of GST were assessed using a range of parameters, including hematological indices (RBC, WBC, and platelet count), relative organ weights (spleen, kidney, thymus, and liver), liver biochemical markers (ALT, AST, ALP, GSH, SOD, and LPO), and the mRNA expression levels of cytokines (IL-2, IL-4 and TNF-α) in the spleen. Further molecular docking studies revealed that triterpenoid saponins of *G. sylvestre* were found to be best TNF-α antagonists. The comprehensive assessment of various parameters provides a holistic view of the potential therapeutic effects of GST on immunosuppression. These parameters were chosen to understand the systemic impact of GST and to provide mechanistic insights into its immunomodulatory properties. Anticancer potential of GST was assessed against a range of cancer cell lines, including COLO205, PC3, SKOV3, B16F10, MCF-7, and MDA-MB-231. Further biocompatibility of GST was evaluated towards normal CHO and HUVEC cell lines using the MTT assay. Remarkably, GST exhibited notable cytotoxicity against breast cancer cell lines, most notably MCF-7. Crucially, GST demonstrated biocompatibility with normal CHO and HUVEC cell lines at lower concentrations. Molecular docking studies revealed robust binding interactions between triterpenoid saponins and breast cancer-associated proteins HER1 and HER2. Further assessments, including physicochemical descriptors, lipophilicity, solubility, pharmacokinetics, drug-likeness, medicinal chemistry and ADMET predictions highlighted potential of triterpenoid saponins as promising pharmacological agents with a favourable safety profile, devoid of significant adverse effects.

**Fig. 1:**
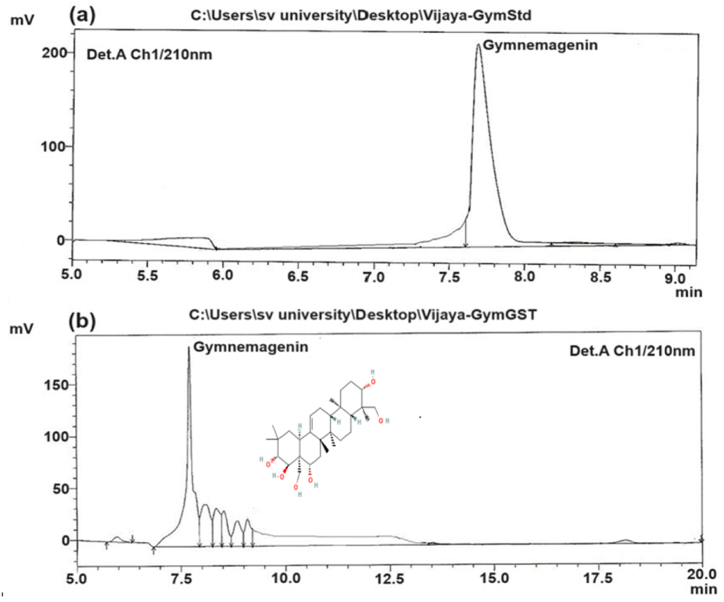
HPLC analysis of (a) Standard gymnemagenin (b) Triterpenoid saponin extract of *G. sylvestre*.

Triterpenoid saponins isolated from *G. sylvestre* are collectively termed as gymnemic acids (GAs). The leaves of *G. sylvestre* are recognized to house 18 distinct types of gymnemic acids, denoted as GA I to GA XVIII. These gymnemic acids are characterized by a shared oleanane-type triterpenoid backbone, commonly referred to as gymnemagenin. subtle differences in the sugar molecules attached to gymnemagenin result in 18 different types of gymnemic acids. This complexity likely contributes to the plant's diverse pharmacological activities. Consequently, gymnemagenin acts as a reference standard for confirming the presence or absence of these gymnemic acids, representing whole triterpenoid saponins in *G. sylvestre*. In the current study, HPLC analysis of the GST sample displayed a predominant peak with a retention time of 7.8 min, alongside minor peaks corresponding to trace impurities or other associated compounds (Fig. 1). This chromatographic pattern was juxtaposed with that of a standard gymnemagenin sample possessing 95 % purity. The HPLC analysis unequivocally confirmed the presence of gymnemic acids in the GST sample. These gymnemic acids are presumed to be the bioactive constituents responsible for the hepatoprotective, immunomodulatory and anticancer properties attributed to *G. sylvestre*.

### 3.1 Effects of GST on hematological parameters in immunosuppressed rats

Hematological parameters, including total RBC, WBC, and platelet counts, were assessed using an automated hematology analyzer across all experimental groups throughout the study period. The evaluation of these parameters is vital in hepatoprotective, oxidative stress, and immunomodulatory studies as they reflect the overall health, immune response, and potential oxidative stress levels of the organism. Animals administered with GST alone did not display significant alterations in RBC, WBC, and platelet counts. Conversely, PRD treatment induced a notable reduction in these hematological parameters compared to both control and GST-alone groups (Table 1). Co-administration of LEV to PRD-induced immunosuppressed rats resulted in a significant enhancement of RBC, WBC, and platelet counts. Similarly, the administration of GST to immunosuppressed rats also led to a significant augmentation of these hematological parameters. Notably, GST administration alone did not induce significant changes in the differential counts of neutrophils, eosinophils, monocytes, and lymphocytes compared to control rats. PRD treatment, however, led to a significant reduction in the counts of neutrophils, monocytes, and lymphocytes compared to both control and GST groups. Co-administration of GST/LEV to PRD-induced immunosuppressed rats resulted in a significant enhancement of these differential counts. Additionally, PRD-induced immunosuppressed rats exhibited a significant decrease in hemoglobin content compared to control and GST groups. Treatment with GST and LEV normalized the hemoglobin content to control levels. These results indicate that GST mitigates the adverse effects of PRD on the hematological parameters of rats, demonstrating its potential antianemic, antileukopenic, and anti-thrombocytopenic activities in immunosuppressed rats (Table 1).

The bone marrow serves as an immune-regulatory organ pivotal for modulating immunity and is a primary target for potential cytotoxic drugs, immune therapeutics, and immune vaccinations [43]. The observed hematological effects of PRD treatment may be attributed to the depletion of stem cells and the compromised ability of the bone marrow to regenerate new blood cells. In the current study, GST and LEV treatments effectively reversed the myelosuppression induced by PRD, facilitating hematopoietic recovery. These findings are consistent with previous studies. Hemalatha et al. [30] reported that saponins derived from *Ocimum sanctum* significantly increased hemoglobin concentration, RBC, and WBC counts in PRD-induced myelosuppressed Swiss albino mice. Sadigh-Eteghad et al. [44] demonstrated that *Echinacea purpurea* extracts significantly elevated differential WBC counts. Methanolic extracts of *Moringa olifera* were shown to increase WBC, neutrophil, and lymphocyte counts in myelosuppressed Wistar albino rats [45]. Sanjeev et al. [46] found that saponins from *Pongamia glabra* mitigated cyclophosphamide-induced myelosuppression in rats by modulating bone marrow activity. Furthermore, the methanolic extract of *Cardiospermum halicacabum*, rich in saponins, counteracted myelosuppressive effects induced in mice [47]. The importance of these hematological parameters, along with bone marrow function, cannot be overstated in the context of hepatoprotective, oxidative stress, and immunomodulatory studies, as they collectively provide insights into systemic health, immune status, and potential oxidative damage.

### 3.2 Effects of GST on selective organ weights in immunosuppressed rats

The spleen, thymus, liver, and kidney play crucial roles in hepatoprotective, antioxidant, and immunomodulatory studies. The spleen and thymus are central to the immune system, producing and maturing immune cells, respectively. The liver is essential for detoxification and metabolic processes, while the kidney plays a pivotal role in filtration and excretion. Therefore, assessing the relative weights of these organs provides valuable insights into the overall health and immune response of the organism, further validating the efficacy of triterpenoid saponins like GST in these therapeutic areas.

**Fig. 2:**
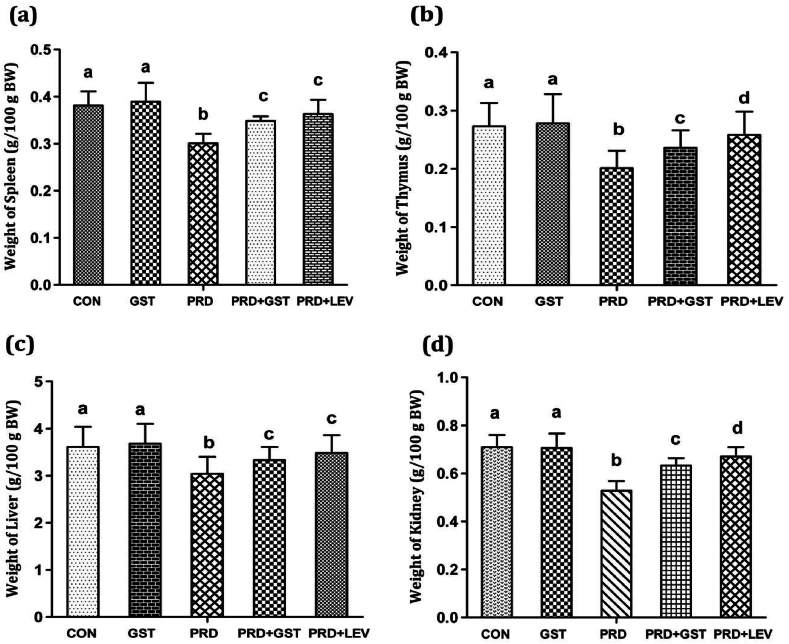
Effect of *G. sylvestre* triterpenoid saponins on the relative organ weights of (a). Spleen, (b). Thymus, (c). Liver, and (d). Kidney in control and treated rats. **CON**: Control; **GST**: Triterpenoid saponin extract; **PRD**: Prednisolone. **PRD + GST**: Prednisolone + Extract; **PRD + LEV**: Prednisolone + Levamisole. The data is represented in the form of a bar graph and plotted using mean ± SD of 6 individual rats. Bars having different superscript significantly differ from each other i.e. (P < 0.05). Bars having same superscript may not differ significantly from each other.

In this study, the relative organ weights of the spleen, thymus, liver, and kidney are represented (Fig. 2a–d). Treatment with PRD significantly reduced the relative weights of these organs, indicating potential organ atrophy due to immunosuppression. Conversely, administration of the GST to immunosuppressed rats resulted in a substantial enhancement in the relative weights of the spleen, thymus, liver, and kidney. Consistent with our findings, Pragathi et al. [15] reported that triterpenoid saponins derived from *Centella asiatica* increased the relative organ weights of the liver, spleen, and thymus in cyclophosphamide-induced immunosuppressed rats. Similarly, Mohammad et al. [48] demonstrated that triterpenoid saponins extracted from *Coffea arabica* elevated the weights of the thymus and spleen in immunosuppressed rats. These studies corroborate the immunomodulatory effects of triterpenoid saponins and highlight their potential in mitigating the adverse effects of immunosuppression on organ weights.

### 3.3 Effects of GST on LPO and GSH levels in immunosuppressed rats

**Fig. 3:**
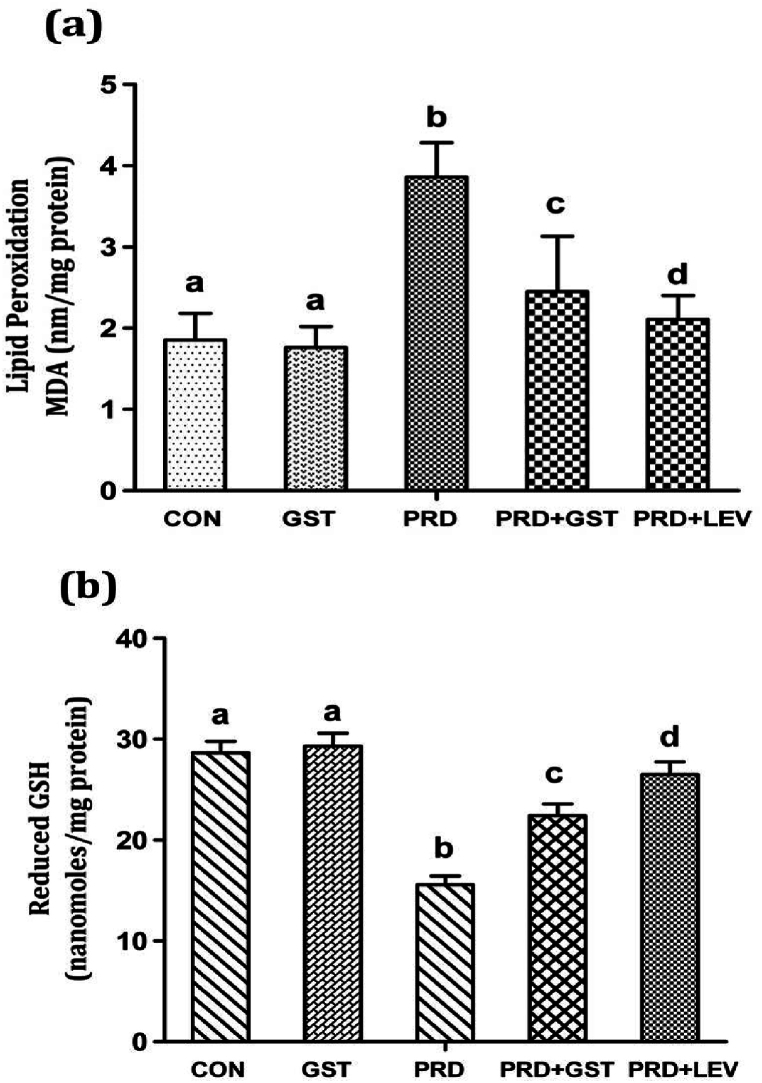
Effect of triterpenoid saponin extract of *G. sylvestre* on (a) Lipid peroxidation (LPO) and (b) Reduced glutathione (GSH) levels in the liver of control and treated rats. **CON**: Control; **GST**: Triterpenoid saponin extract; **PRD**: Prednisolone. **PRD + GST**: Prednisolone + Extract; **PRD + LEV**: Prednisolone + Levamisole. The data is represented in the form of a bar graph and plotted using mean ± SD of 6 individual rats. Bars having different superscript significantly differ from each other i.e. (P < 0.05). Bars having same superscript may not differ significantly from each other.

LPO levels were assessed by measuring MDA content (Fig. 3a). Elevated MDA levels (3.79 nm/mg protein) were observed in the immunosuppressed rats induced by PRD, compared to the normal control (1.87 nm/mg protein). Co-administration of GST significantly reduced MDA levels to 2.45 nm/mg protein. The standard immunostimulant, levamisole (LEV), also reduced MDA levels in the immunosuppressed rats. Thus, the co-administration of GST/LEV along with PRD effectively mitigated the elevated MDA levels induced by PRD. Lipid peroxidation serves as a principal indicator of oxidative damage mediated by reactive oxygen species (ROS). Elevated LPO has been associated with decreased membrane fluidity, inactivation of membrane-bound receptors, impaired membrane function, and increased nonspecific permeability to ions and molecules [49]. In the present study, the administration of GST resulted in a significant reduction in LPO levels, underscoring the antioxidant activity of the triterpenoid saponins and their potential to mitigate PRD-induced oxidative stress.

Glutathione is a tripeptide composed of glutamate, cysteine, and glycine, playing a crucial role in cellular antioxidant defense against free radicals, lipid peroxides, drugs, exogenous and endogenous toxic substances, and heavy metals. It acts as a protective antioxidant in both animals and plants [50]. During this protective process, glutathione disulfide bonds (-SH groups) are reduced to its oxidized form, glutathione disulfide (GSSG). GSH levels were determined using Ellman's reagent (Fig. 3b). PRD-induced immunosuppressed rats exhibited a substantial reduction (54.31 % of normal control) in GSH levels. Co-administration of the GST significantly elevated GSH levels to 78.25 %, while LEV treatment further enhanced GSH levels to 92.4 %. In the current study, administration of GST promoted GSH production. These findings are consistent with previous studies reporting that triterpenoid saponins from *Centella asiatica* decrease lipid peroxidation and enhance GSH levels, thus protecting against free radical-induced damage [15]. Leaf extracts of *Leptadenia reticulata* have been shown to increase GSH activity and decrease LPO levels in immunosuppressed rats [51]. Furthermore, saponins from *Ocimum sanctum* were reported to exhibit potent antioxidant and immunomodulatory activities in myelosuppressed Swiss albino mice [30]. Additionally, the leaf extract of *Ficus benghalensis* has been reported to possess antioxidant and immunomodulatory properties [52], while the root extract of *Actinidia kolomikta* and seed extract of *Citrullus lanatus* have demonstrated similar antioxidant and immunomodulatory activities [53,54].

The assessment of LPO and GSH levels holds significant importance in evaluating antioxidant, hepatoprotective, and immunomodulatory properties of therapeutic agents. Elevated LPO levels signify increased oxidative stress and damage to cellular membranes, proteins, and DNA [55]. Conversely, GSH acts as a primary antioxidant defense mechanism against oxidative stress, playing a pivotal role in detoxification and maintaining cellular redox homeostasis [56]. Therefore, agents that can reduce LPO levels and enhance GSH production, such as GST observed in this study, hold promise in mitigating oxidative stress-induced cellular damage and promoting liver health. The hepatoprotective and antioxidant effects of triterpenoid saponins from *G. sylvestre*, demonstrated by the reduction in MDA levels and enhancement of GSH levels, highlight their therapeutic potential in protecting against oxidative stress-induced hepatocellular injury.

### 3.4 Effects of GST on liver function markers in immunosuppressed rats

**Fig. 4:**
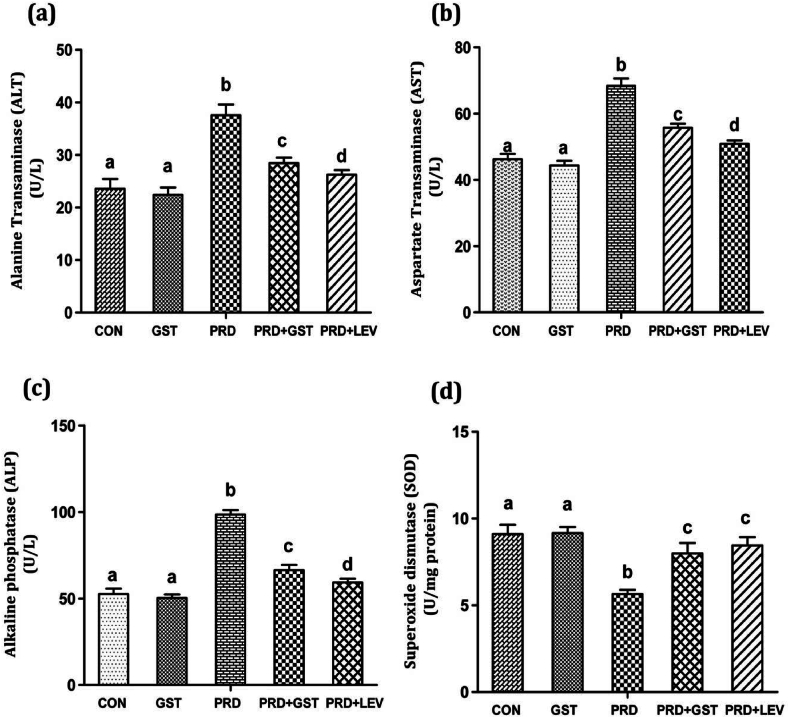
Effect of triterpenoid saponin extract of *G.sylvestre* on liver enzymes (a). Alanine transaminase (ALT), (b). Aspartate transaminase (AST), (c). Alkaline phosphatase, and (d) Superoxide dismutase (SOD) levels in control and treated rats. **CON**: Control; **GST**: Triterpenoid saponin extract; **PRD**: Prednisolone. **PRD + GST**: Prednisolone + Extract; **PRD + LEV**: Prednisolone + Levamisole. The data is represented in the form of a bar graph and plotted using mean ± SD of 6 individual rats. Bars having different superscript significantly differ from each other i.e. (P < 0.05). Bars having same superscript may not differ significantly from each other.

ALT and AST levels were determined in all experimental groups to evaluate liver function (Fig. 4a and b). Animals treated solely with GST did not exhibit significant alterations in ALT and AST levels. In contrast, PRD-induced immunosuppressed rats demonstrated a marked elevation in ALT levels (37.56 U/L) compared to the control group (23.58 U/L). Co-administration of GST to PRD-treated rats led to a significant reduction in ALT levels (28.43 U/L), as did the administration of levamisole (LEV) (26.24 U/L) compared to PRD-treated animals alone. Similarly, PRD-treated rats exhibited a significant increase in AST levels (68.35 U/L) compared to controls (46.23 U/L). Administration of GST and LEV in combination with PRD led to a notable reduction in AST levels (55.74 U/L for GST + PRD and 54.19 U/L for LEV + PRD) compared to PRD alone-treated rats. These findings indicate that GST effectively mitigates the elevated ALT and AST levels induced by immunosuppression.

In addition to ALT and AST, ALP levels were also assessed to further evaluate liver function (Fig. 4c). Rats treated solely with GST did not exhibit any significant changes in ALP levels. PRD-induced immunosuppressed rats displayed elevated ALP levels (15.56 KA) compared to control rats (7.65 KA). However, co-administration of GST and LEV led to a significant reduction in ALP levels (10.85 KA for GST + PRD and 10.01 KA for LEV + PRD). These results suggest that both GST and LEV contribute to the restoration of ALP levels in immunosuppressed rats.

ALT, AST, and ALP are predominantly found in liver cells and serve as integral components of the liver function test (LFT) panel, essential for diagnosing hepatocellular injury and assessing liver health [57]. Elevated levels of these enzymes often indicate various liver conditions, including acute and chronic hepatitis, bile duct obstruction, liver cirrhosis, inflammation, and hepatocyte loss. Additionally, increased ALT, AST, and ALP levels can be indicative of liver damage induced by many cytotoxic drugs, medications, and oxidative stress [58]. Oxidative stress plays a pivotal role in the pathogenesis of liver diseases by promoting hepatocyte injury, inflammation, and fibrosis. Reactive oxygen species (ROS) and reactive nitrogen species (RNS) generated during oxidative stress can cause lipid peroxidation, protein oxidation, and DNA damage, leading to cellular dysfunction and death [59]. The liver, being a central organ in the metabolism and detoxification of xenobiotics, is particularly susceptible to oxidative damage. Elevated levels of ALT, AST, and ALP are not only markers of hepatocellular injury but also reflect the extent of oxidative stress and the liver's antioxidant defense mechanisms [60]. In conditions of oxidative stress, the liver's antioxidant capacity may be overwhelmed, leading to the activation of inflammatory pathways and further exacerbating liver damage. By scavenging free radicals, reducing oxidative stress, and modulating inflammatory responses, GST may help in preserving hepatocellular integrity, improving liver function, and mitigating the elevated levels of ALT, AST, and ALP induced by oxidative stress and hepatotoxic agents. Understanding the relationship between liver health, oxidative stress, and the levels of ALT, AST, and ALP is crucial for the early diagnosis, management, and treatment of liver diseases. The potential hepatoprotective and antioxidant properties of GST highlight its therapeutic promise in protecting against liver damage induced by oxidative stress and hepatotoxic agents. Further research is warranted to elucidate the underlying molecular mechanisms and to explore the clinical applications of GST in hepatoprotection and liver disease management.

In the current study, the immunosuppressive drug PRD significantly elevated the levels of ALT, AST, and ALP, indicative of hepatocellular injury. The hepatoprotective effects of GST observed in this study are consistent with previous reports. Triterpene saponins isolated from various sources have been shown to exhibit antihepatotoxic activity by inhibiting ALT, AST, and ALP levels. For instance, *Nigella sativa* seed extracts containing saponins exhibited a significant decrease in ALT, AST, and ALP activities in carbon tetrachloride (CCl4)-induced hepatotoxicity in rats [61]. Similarly, saponin extract from the root of *Garcinia kola* reduced the levels of these liver enzymes in paracetamol-induced hepatotoxicity in albino rats [62]. Liping et al. [63] reported the hepatoprotective activity of total saponins from *Actinidia valvata* against CCl4-induced hepatotoxicity in rats by lowering ALT, AST, and ALP levels. Lijie et al. [64] reported the hepatoprotective effects of triterpenoid saponins isolated from *Schizandra chinensis* against alcohol-induced liver injury. The observed hepatoprotective effects of GST in this study highlight its potential therapeutic value in protecting against hepatocellular injuries induced by drugs like PRD.

### 3.5 Effects of GST on SOD levels in immunosuppressed rats

SOD levels were assessed in both control and treated groups. SOD inhibits the reduction of NBT to formazan blue, serving as an indicator of its presence in the sample. Rats with PRD-induced immunosuppression exhibited a significant reduction in SOD levels, registering at 5.65 U/mg protein compared to the normal control level of 9.1 U/mg protein (Fig. 4d). Administration of the triterpenoid extract from *G. sylvestre* to immunosuppressed rats led to a notable increase in SOD levels, elevating them to 7.98 U/mg protein. Similarly, treatment with LEV resulted in a substantial enhancement of SOD levels, reaching up to 8.45 U/mg protein (Fig. 4d).

SOD is a crucial component of the body's internal antioxidant defense mechanisms, playing an indispensable role in mitigating oxidative stress—a phenomenon implicated in the pathogenesis of numerous life-threatening diseases [65]. Effective protection against oxidative damage necessitates elevated levels of antioxidant enzymes such as SOD. In this study, PRD-induced immunosuppressed rats manifested a marked reduction in SOD levels. However, administration of the triterpenoid extract derived from *G. sylvestre* effectively restored SOD levels. This finding is corroborated by prior research. For instance, Liping et al. [63] demonstrated that saponins extracted from *Actinidia valvata* could restore the levels of oxidative enzymes, including SOD and CAT, in rats with CCl4-induced acute liver damage. Additionally, *Nigella sativa* seed extracts, rich in saponins, were found to elevate diminished hepatic SOD levels in rats subjected to CCl4-induced hepatotoxicity [61].

The estimation of SOD levels holds paramount importance in understanding the antioxidant defense mechanisms of the body. SOD serves as the first line of defense against superoxide radicals, converting them into hydrogen peroxide and oxygen, thereby preventing the formation of highly reactive and damaging hydroxyl radicals [66]. In the context of hepatoprotection, maintaining optimal SOD levels is crucial. The liver is particularly susceptible to oxidative damage due to its role in detoxification and metabolism. Reduced SOD activity can exacerbate oxidative stress in hepatic tissues, leading to inflammation, fibrosis, and ultimately, liver dysfunction [67]. In conclusion, the restoration of SOD levels through the administration of triterpenoid extract from *G. sylvestre* and LEV underscores the potential hepatoprotective effects of these agents. This study highlights the significance of SOD estimation in evaluating the antioxidant status, oxidative stress levels, and potential therapeutic interventions for liver-related disorders.

### 3.6 Effect of GST on mRNA expression levels of cytokines IL-2, IL-4 and TNF-α in the spleen

**Fig. 5:**
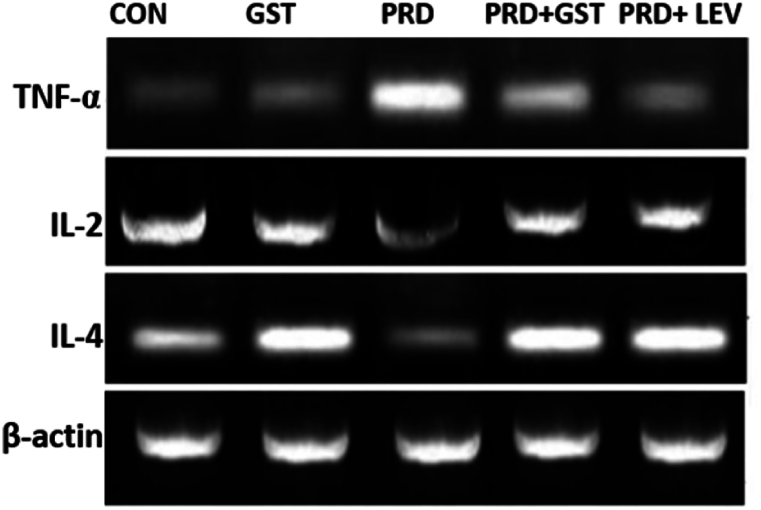
Gel Electrophoresis of RT-PCR Products Showing Differential Expression of IL-2, IL-4, and TNF-α mRNA in spleen tissue of PRD-Induced Immunosuppressed Rats Treated with *G. sylvestre* Triterpenoid Saponin Extract (GST) and Levamisole (LEV). Con.

**Fig. 6:**
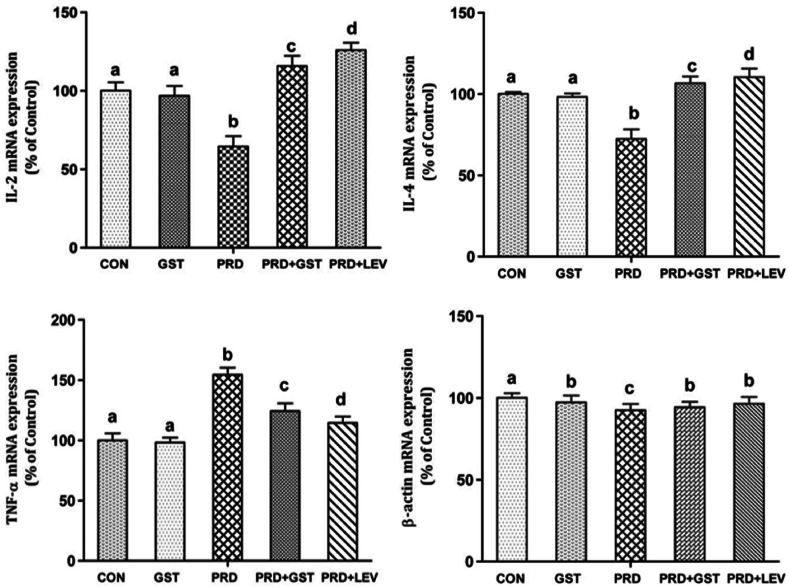
Quantitative Analysis of IL-2, IL-4, TNF-α and β-actin mRNA Expression Levels in Spleen Tissue of PRD-Induced Immunosuppressed Rats Treated with *G. sylvestre* Triterpenoid Saponin Extract (GST) and Levamisole (LEV). The data illustrate the modulation of cytokine expression following treatment, indicating the efficacy of GST and LEV in restoring immune function. The data is represented in the form of a bar graph and plotted using means ± SD of 6 individual rats. Bars having different superscript significantly differ from each other i.e. (P < 0.05). Bars having same superscript may not differ significantly from each other.

The semi-quantitative RT-PCR analysis of immunomodulating markers (IL-2, IL-4 and TNF-α) gene expression in the experimental animals was conducted in conjunction with the co-expression of the housekeeping gene, β-actin, which was constitutively expressed in all experimental groups. The gene expression levels of IL-2, IL-4 and TNF-α in both control and treated groups are represented (Fig. 5) In rats with PRD-induced immunosuppression, there was a notable decrease in the expression level of IL-2 mRNA compared to the normal control group, with levels reaching 64.51 % of the normal control (Fig. 6) However, administration of GST to these immunosuppressed rats led to a significant increase in IL-2 mRNA expression levels, reaching 115.82 % of the normal control. Similarly, administration of LEV to the immunosuppressed rats resulted in a substantial enhancement of IL-2 mRNA expression levels, reaching 124.63 % of the normal control. Thus, both GST and LEV administration to PRD-treated animals significantly enhanced the gene expression levels of IL-2. In the PRD-induced immunosuppressed group, a decreased mRNA expression level of IL-4 was evident, which was 72.38 % of the normal control. However, upon administration of GST to the immunosuppressed rats, there was a significant increase in IL-4 expression levels, reaching 106.55 % of the normal control. Additionally, LEV administration to the immunosuppressed rats led to a notable enhancement in IL-4 mRNA expression levels, reaching 110.42 % of the normal control. Increased mRNA expression levels of TNF-α (156.43 %) were observed when treated with PRD. However, co-administration of GST with PRD significantly decreased the mRNA expression levels of TNF-α to 123.67 %. Similarly, co-administration of Levamisole with PRD significantly reduced the expression levels of TNF-α to 115.26 %.

IL-2 is a polypeptide composed of 133 amino acid residues and is primarily produced by T lymphocytes. IL-2 is essential for the activation of T lymphocytes, and its binding to specific receptors on T helper (Th) cells stimulates T lymphocyte proliferation and the release of various cytokines from these cells [68,69]. IL-2 is also necessary for the generation of CD8^+^ cytolytic T (Tc) cells, which play a significant role in antiviral responses [70]. Furthermore, IL-2 enhances the effector function of natural killer (NK) cells, thereby bolstering the immune system's ability to eliminate tumor cells and impede blood flow to tumors [71]. On the other hand, IL-4 is a pleiotropic cytokine primarily produced by Th2 cells, mast cells, and NK cells. It is also produced by other specialized subsets of T cells, basophils, and eosinophils [72]. IL-4 plays a pivotal role in regulating the differentiation of antigen-activated naive T cells. These activated T cells subsequently produce IL-4 along with other Th2-type cytokines [73]. IL-4 is essential for the production of IgE and is the principal cytokine responsible for inducing isotype switching in B cells from IgG expression to IgE and IgG. Consequently, IL-4 plays a critical role in regulating allergic diseases [74]. In this study, administration of PRD led to a significant reduction in the mRNA levels of IL-2 and IL-4. In contrast, treatment with GST resulted in markedly elevated levels of IL-2 and IL-4 gene expression compared to rats treated with PRD alone. Given that triterpenoid saponins increased the expression of IL-2 and IL-4 genes, these findings suggest that triterpenoid saponins may exert an up-regulatory influence on the production of these cytokines, thereby stimulating the immune system. Our findings align with prior research. Ursolic acid, found in plants like *Rosmarinus officinalis* L. and *Sambucus nigra*, boosts IL-2 production in T-cells [75,76]. Glycyrrhizin from *Glycyrrhiza glabra* L. enhances IL-2 expression, while Ganoderic acids from *Ganoderma lucidum* elevate IL-2 levels [[77], [78], [79]]. Astragalosides (I, II, IV, and VII) from *Astragalus* L. species and Ginsenoside Rg1 and Re from *Panax ginseng* also promote IL-2 production [[80], [81], [82], [83], [84]]. Similarly, platycodigenin, a triterpenoid saponin from *Platycodon grandifloras* and hyoscyamoside from *Hyoscymus niger* found to induce IL-4 expression levels [[85], [86], [87]].

Tumor necrosis factor-alpha (TNF-α) is a potent pro-inflammatory cytokine that plays a central role in regulating immune responses, inflammation, and apoptosis [88]. It is primarily produced by activated macrophages, T cells, and other immune cells in response to various stimuli, including infection, tissue injury, and stress [89]. In the context of the spleen, TNF-α plays a pivotal role in modulating immune responses. However, dysregulation of TNF-α expression in the spleen can lead to excessive inflammation and tissue damage. Elevated levels of TNF-α have been associated with various inflammatory and autoimmune diseases, such as rheumatoid arthritis, inflammatory bowel disease, and sepsis [90]. In studies involving immunosuppressed animals or conditions of immune dysregulation, alterations in spleen TNF-α levels have been observed. In our study, TNF-α expression levels were significantly increased following PRD treatment. However, administration of GST effectively reduced these elevated levels, demonstrating their potential anti-inflammatory effects and ability to modulate TNF-α expression in the spleen. Our findings are consistent with previous research. Pragathi et al. demonstrated that triterpene saponins from *Centella asiatica* effectively attenuated elevated TNF-α levels [15]. Glycyrrhizin, a triterpenoid from *Glycyrrhiza glabra* L. decreased the production of proinflammatory cytokine TNF-α [77,78]. Boswellic acids from *Boswellia serrata* downregulated the expression levels of TNF-α [91]. Tormentic acid, a triterpenoid saponin extracted from *Rosa rugosa*, efficiently suppressed LPS-induced TNF-α expression [92]. Triterpene saponins from *Pulsatilla koreana* were found to inhibit the expression of TNF-α mRNA [93]. Triterpene saponins derived from *Caulophyllum thalictroides* exert anti-inflammatory effects by inhibiting TNF-α [94]. Strategies aimed at modulating TNF-α activity and expression have garnered significant therapeutic interest, leading to the development of TNF-α inhibitors. By binding to TNF-α molecules, these inhibitors prevent them from interacting with their receptors, thereby inhibiting inflammatory signaling pathways and alleviating symptoms in patients suffering from inflammatory and autoimmune diseases. Our molecular docking studies between TNF-α and triterpenoid saponins from *G. sylvestre* have demonstrated that these triterpenoids effectively inhibit TNF-α. This research lays the groundwork for the development of biological TNF-α inhibitors that hold promise as potential pharmaceutical drugs. Overall, the development and production of TNF-α inhibitors signify a significant advancement in the treatment of inflammatory and autoimmune diseases.

The evaluation of hepatoprotective and immunomodulatory potential of the triterpenoid saponins from *G. sylvestre* resulted in a significant increase in the hematological profile and relative organ weights of the spleen, thymus, liver, and kidneys in prednisolone-induced immunosuppressed rats. Additionally, the triterpenoid saponins led to a significant increase in GSH and SOD levels, while significantly decreasing LPO, ALT, AST, and ALP levels in prednisolone-induced immunosuppressed rats. Furthermore, triterpenoid saponins exhibited significant upregulation of IL-2 and IL-4 while downregulating TNF-α cytokines, in immunosuppressed rats. The findings of this study revealed that *G. sylvestre* possesses potential immunomodulatory (immunostimulating) and hepatoprotective (antioxidant) activities, which could play a significant role in mitigating the risks associated with immunosuppressive conditions or immunodeficiency disorders.

### 3.7 Anticancer activity of GST

**Fig. 7:**
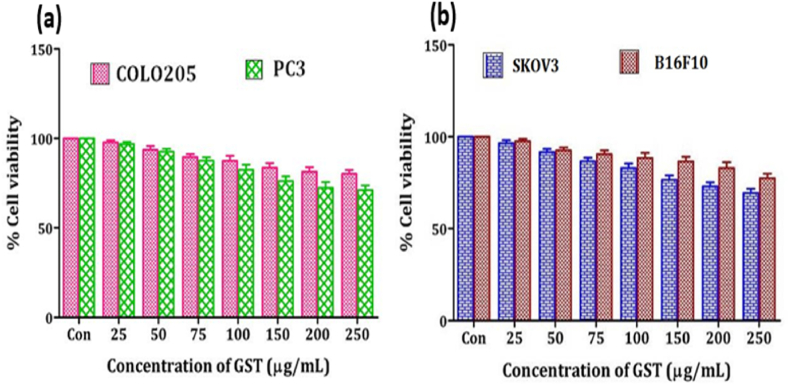
Effect of triterpenoid saponin extract of *G. sylvestre* on cell viability of (a) COLO205 (Human Colon Cancer) and PC3 (Human prostate carcinoma) cells (b) cell viability of SKOV3 (Human ovarian cancer) and B16F10 (Mouse Melanoma) cell lines. The data is represented in the form of a bar graph and plotted using means ± SD of 3 individual experiments. Con: Control; GST: Triterpenoid saponin extract of *G. sylvestre*.

The anticancer activity of the triterpenoid saponin extract of *G. sylvestre* (GST) was evaluated against different cancer cell lines including COLO205 (Human colon cancer), PC3 (Human prostate carcinoma), SKOV3 (Human ovarian carcinoma), B16F10 (Mouse melanoma), MCF-7, and MDA-MB-231 (Human breast cancer cell lines) by using MTT assay (cell viability assay). GST did not show any significant cytotoxicity against COLO205 cells. Increase in the concentration of GST could not show significant decrease in the cell viability of COLO205 cells (Fig. 7a). GST showed only 3–20 % cytotoxicity against COLO205 cells. 80 % of the COLO205 cells are viable even at the highest concentration of GST (250 μg/mL) used in this study and the IC_50_ value (concentration required to inhibit 50 % of the cells) was determined as 624.12 μg/mL. The higher IC_50_ value indicates lower cytotoxicity. Hence GST is ineffective against COLO205 cells. Increase in the concentration of GST from 25 to 250 μg/mL decreases the viability of PC3 cells from 97 to 71 %. At the highest concentration of 250 μg/mL used in this study, GST showed 29 % cytotoxicity. IC_50_ concentration was found to be 401.71 μg/mL. Based on the percentage cell viability and IC_50_ values, it is determined that GST is effective against PC3 cells compared to COLO205 Cells (Fig. 7a). Anticancer activity of GST was studied against SKOV3 and B16F10 cells (Fig. 7b). Increase in the concentration of GST decreases the cell viability and increases the cytotoxicity of SKOV3 and B16F10 cells. 69 % of the SKOV3 cells and 78 % of the B16F10 cells are viable at the highest concentrations of GST (250 μg/mL) tested in this study. The IC_50_ values for SKOV3 and B16F10 cells were found to be 395.83 and 609.24 μg/mL. The cytotoxicity of the GST against four cancer cell lines studied as follows SKOV3 (31 %) > PC3 (29 %) > B16F10 (22 %) > COLO205 (20 %).

**Fig. 8:**
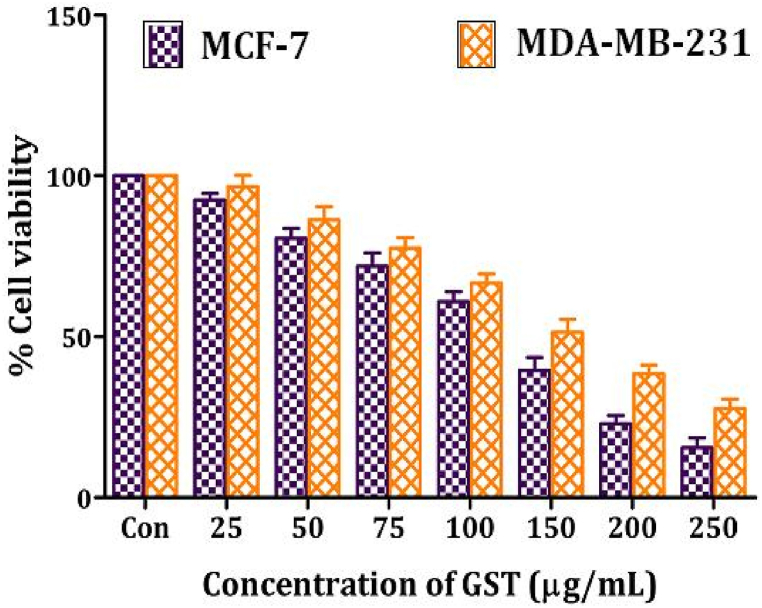
Effect of triterpenoid saponin extract of *G. sylvestre* on cell viability of Breast cancer cell lines MCF-7 and MDA-MB-231. The data is represented in the form of a bar graph and plotted using means ± SD of 3 individual experiments. Con: Control; GST: Triterpenoid saponin extract of *G. sylvestre*.

Further we studied the cytotoxicity of GST against human breast cancer cell lines including MCF-7 and MDA-MB-231 (Fig. 8). The effective cytotoxic activity of GST was reported against MCF-7, and MDA-MB-231 by measuring the number of live cells after 24 h of treatment (MTT assay). Cell viability of the tested cancer cells is decreased with an increasing concentration of GST. Only 15.48 ± 3.01 % (MCF-7), and 27.63 ± 3.02 % (in case of MDA-MB-231) cells are viable (or live) at the highest concentration of GST (250 μg/mL) used in this study. IC_50_ values are determined using linear regression graphs. GST exhibited potent antiproliferative activity and inhibited cell growth of MCF-7, and MDA-MB-231 cells with IC_50_ values of 135.08, and 160.42 μg/mL respectively. This study revealed that triterpenoid saponin extract of *G. sylvestre* (GST) showed effective anticancer activity on different breast cancer cell lines particularly MCF-7.

**Fig. 9:**
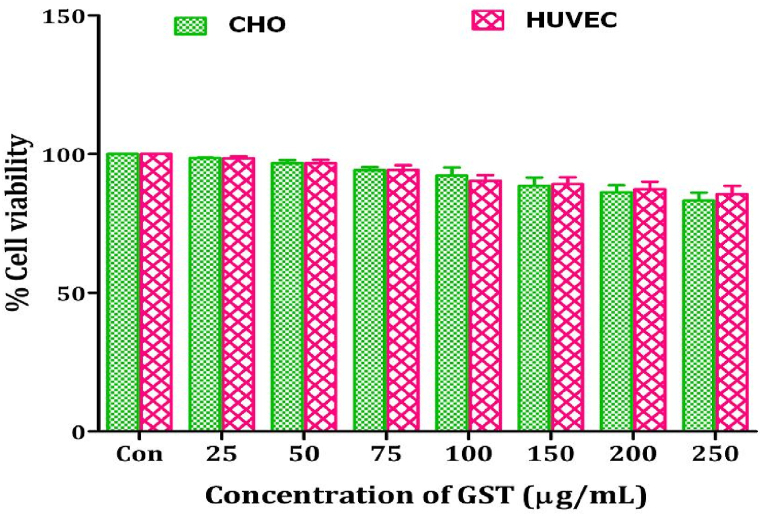
Effect of triterpenoid saponin extract of *G. sylvestre* on cell viability of normal CHO and HUVEC cells. The data is represented in the form of a bar graph and plotted using means ± SD of 3 individual experiments. Con: Control; GST: Triterpenoid saponin extract of *G. sylvestre*.

It is very crucial to check the biocompatibility of pharmaceutical bioactive compounds and their extracts for their successful application in biomedicine and diagnostic fields. The cytotoxicity of GST was checked against normal CHO (Chinese hamster ovary cell line) as well as HUVEC (Human umbilical vein endothelial cells) cell lines to check their biocompatibility, as safety is the main concern, and toxicity against normal cell lines can limit the therapeutic applications. In this study, GST could not show any inhibition against CHO and HUVEC cell lines at lower concentrations. Increase in the concentration of GST could not significantly decrease the cell viability of CHO and HUVEC cells (Fig. 9) Only 17 % (in case of CHO cells) and 15 % (in case of HUVEC cells) cells are found to be nonviable at the highest concentration of GST (250 μg/mL) used in this study. Hence this study revealed that triterpenoid saponins of *G. sylvestre* were found to be biocompatible at lower concentrations.

In the present study, triterpenoid saponins of *G. sylvestre* showed cytotoxic effects on different breast cancer cells but they did not show cytotoxic effects on normal CHO and HUVEC cells. The results are consistent with earlier reports. Saponins from *Gypsohila oldhamiana* induces apoptosis of hepatic carcinoma cells but, they did not show cytotoxic effects on normal liver cells [95]. Saponin isolated from tomato (α tomatine) inhibits the growth and induces the apoptosis of human prostatic carcinoma (PC-3) cells but normal human liver (WRL- 68) and normal human prostate (RWPE-1) cells were insensitive to α tomatine [96] Triterpenoid saponins exhibited their effects on cancer cells with more efficacy and high specificity which suggest that they could exert their cytotoxic activity without significant side-effects. Cancer cell-selectivity of saponin activity is crucial for their systemic tolerability. High specificity of the saponins may concern not only their impact on the cancer and normal cells but also implies their various effects on cancer cells of different origin [97]. In this study, triterpenoid saponins of *G. sylvestre* showed effective cytotoxic effects on breast cancer cell lines. While, they did not show significant cytotoxic effects on COLO205, PC3, SKOV3 and B16F10. These results are consistent with previous studies. For instance, Escin, a saponin isolated from *Aesculus hipocastanum* showed effective cytotoxicity against A-549 (Human lung carcinoma), PANC-1 (Human pancreatic carcinoma) and human acute leukemia Jurkat cells. But, Escin could not show cytotoxicity against SKOV3 [98]. Eight triterpenoid saponins isolated from *Ardisia japonica* selectively inhibited the growth of liver cancer cells (Bel-7402 and HepG2) without affecting the viability of the normal cells (HL-7702) [99]. Saponins from *Lysimachia ciliate* exhibit selectivity for human prostate cancer and normal cells [100].

### 3.8 Molecular docking studies

**Fig. 10:**
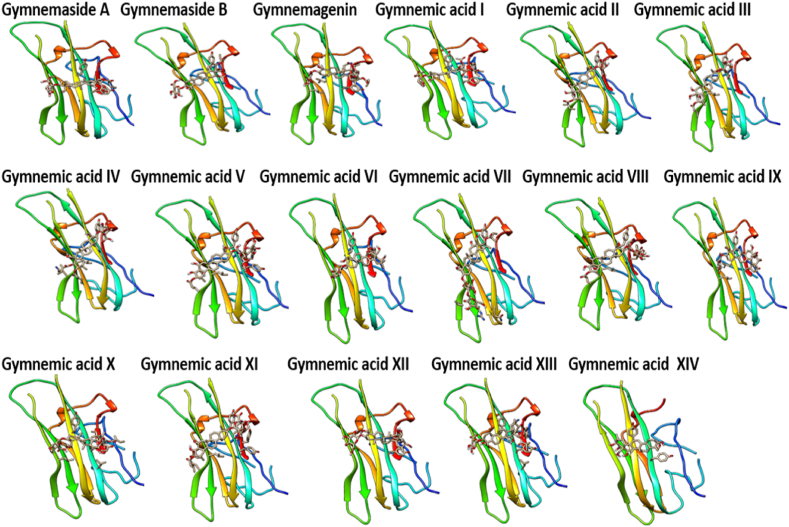
Visualizing the interaction dynamics of Gymnemic acids with TNF-α: A 3D Perspective.

*In viv*o immunomodulatory studies in this investigation revealed that triterpenoid saponins from *G. sylvestre* effectively suppressed elevated levels of TNF-α in immunosuppressed rats. Further molecular docking studies were conducted between triterpenoid saponins and TNF-α with the aim of identifying potential TNF-α inhibitors. Fig. 10 provides clear representations of the 3D interactions between various gymnemic acids (ligands) and TNF-α (protein). Additionally, a supplementary file (Fig. 1S) illustrates a 2D model of the gymnemic acids-TNFα complex, showcasing active site residues and their binding interactions. The binding energies, active site residues, hydrogen bond formations, and the corresponding residues involved were tabulated (Table 2). With a binding affinity of −7.5 kcal/mol, Gymnemic acid VI forms one hydrogen bond with Ser-A60 and engages in electrostatic interactions with active site residues such as Tyr-A59, AsnA-92, Leu-A93, Leu-A94, Ser-A95, Tyr-B119, Leu-B120, Gly-B121, and Gly-B122. Gymnemic acid IX has a binding affinity of −7.3 kcal/mol and forms one hydrogen bond with Tyr-B151 and it shows electrostatic interactions with residues like Tyr-A59, Gln-A61, AsnA-92, Leu-A93, Leu-A94, Ser-A95, Tyr-B119, Leu-B120, Gly-B121, and Tyr-B151. With a binding affinity of −7.3 kcal/mol, Gymnemic acid I interacts through electrostatic interactions with Tyr-A59, Gln-A61, Leu-A94, Ser-A95, Tyr-B119, Leu-B120, Gly-B121, and Tyr-B151. Gymnemic acid XIII and XIV have a binding affinity of −7.2 kcal/mol and form one hydrogen bond with Tyr-B151. They also exhibit pi-pi interactions with residues such as Leu-A57, Tyr-A59, Gln-A61, Ala-A96, Tyr-B119, Leu-B120, Gly-B121, and Tyr-B151. With a binding affinity of −7.1 kcal/mol, Gymnemic acid XII forms three hydrogen bonds with Ser-A60, AsnA-92, and Leu-B120. Gymnemic acid VII and VIII bind with a binding affinity of −7.1 kcal/mol and form a hydrogen bond with Ser-A95 and exhibit electrostatic interactions with other active site residues. Gymnemic acid XI has a binding affinity of −7.0 kcal/mol and forms a hydrogen bond with Gly-C121. Gymnemic acid III and IV interact with a binding affinity of −7.0 kcal/mol and form a hydrogen bond with LeuA-93. With a binding affinity of −6.8 kcal/mol, Gymnemic acid V forms a hydrogen bond with Ser-A95 and exhibits electrostatic interactions with other active site residues. Gymnemic acid X has a binding affinity of −6.7 kcal/mol and forms a hydrogen bond with Ser-A95. Gymnemagenin shows a binding energy of −6.4 kcal/mol and forms electrostatic interactions with active site residues such as Tyr-A59, Tyr-B119, Gly-B121, and Leu-B120. Gymnemaside A has a binding affinity of −6.5 kcal/mol and forms two hydrogen bonds with Ser-A95. Gymnemic acid II interacts with a binding affinity of −6.6 kcal/mol and forms two hydrogen bonds with Ser-A95. Gymnemaside B binds with a binding affinity of −6.2 kcal/mol and forms two hydrogen bonds with Ser-A95. Gymnemic acid VI with a binding energy of −7.5 kcal/mol followed by Gymnemic acid IX and I (−7.3 kcal/mol), Gymnemic acid XIII and XIV (−7.2 kcal/mol), demonstrate stronger binding affinities towards TNF-α and are thus potential inhibitors based on their molecular docking interactions. Amino acid residues such as Tyr-A59, Ser-A60, Gln-A61, ValA-91, AsnA-92, Leu-A93, Leu-A94, Ser-A95, Ala-A96, Tyr-B119, Leu-B120, Gly-B121, and Tyr-B151 were identified as key residues involved in the binding interactions, indicating their critical role in the binding mechanism of *G. sylvestre* compounds with TNF-α. In many of the ligand-protein (Gymnemic acid-TNF-α) complexes, a hydrophobic cleft formed by the residues ValA-91, Leu-A93, Leu-A94, Ala-A96, Tyr-B119, Leu-B120, Gly-B121, and Phe-B124 provided additional stability to the Gymnemic acid-TNF-α complex. The diversity in binding affinities, active site interactions, and hydrogen bond formations among the *G. sylvestre* compounds offers insights into their differential inhibitory potentials against TNF-α.

**Table 2:** Binding energies, No. of H-bonds, Active site residues and H-bond interactions between triterpenoid saponins (Ligand) and TNF-α

Compound name	ΔG (Kcal/mol)	Active site Residues	No. of H-bonds	H- bond residues
Gymnemaside A	−6.5	Tyr-A59, Ser-A60, Ser-A95, Ala-A96, Tyr-B119, Leu-B120, Gly-B121, Tyr-B151	2	Ser-A95, Ser-A95
Gymnemaside B	−6.2	Leu-A94, Ser-A95, Ala-A96, Tyr-B119, Leu-B120, Gly-B121	2	Ser-A95, Ser-A95
Gymnemagenin	−6.4	Tyr-A59, Tyr-B119, Gly-B121, Leu-B120	0	
Gymnemic acid I	−7.3	Tyr-A59, Gln-A61, Leu-A94, Ser-A95, Tyr-B119, Leu-B120, Gly-B121, Tyr-B151	0	
Gymnemic acid II	−6.6	Tyr-A59, Ser-A60, Ser-A95, Ala-A96, Tyr-B119, Leu-B120, Gly-B121, Tyr-B151	2	Ser-A95, Ser-A95
Gymnemic acid III	−7.0	ValA-91, AsnA-92, Leu-A93, Leu-A94, Ser-A95, Tyr-B119, Leu-B120, Gly-B121	1	LeuA-93
Gymnemic acid IV	−7.0	ValA-91, AsnA-92, Leu-A93, Leu-A94, Ser-A95, Tyr-B119, Leu-B120, Gly-B121	1	LeuA-93
Gymnemic acid V	−6.8	AsnA-92, Leu-A93, Leu-A94, Ser-A95, Tyr-B119, Leu-B120, Gly-B121, Phe-B124	1	Ser-A95
Gymnemic acid VI	−7.5	Tyr-A59, Ser-A60, AsnA-92, Leu-A93, Leu-A94, Ser-A95, Tyr-B119, Leu-B120, Gly-B121, Gly-B122, Tyr-B151	1	Ser-A60
Gymnemic acid VII	−7.1	Tyr-A59, Leu-A94, Ser-A95, Tyr-B119, Leu-B120, Gly-B121, Tyr-B151	1	Ser-A95
Gymnemic acid VIII	−7.1	Tyr-A59, Leu-A94, Ser-A95, Tyr-B119, Leu-B120, Gly-B121, Tyr-B151	1	Ser-A95
Gymnemic acid IX	−7.3	Tyr-A59, Gln-A61, AsnA-92, Leu-A93, Leu-A94, Ser-A95, Tyr-B119, Leu-B120, Gly-B121, Tyr-B151	2	Tyr-B151
Gymnemic acid X	−6.7	Leu-A57, Tyr-A59, Leu-A94, Ser-A95, Tyr-B119, Leu-B120, Gly-B121	1	Ser-A95
Gymnemic acid XI	−7.0	His-A15, Leu-B57, Tyr-B59, Leu-B94, Ser-B95, Tyr-C119, Leu-C120, Gly-C121, Tyr-C151	1	Gly-C121
Gymnemic acid XII	−7.1	Tyr-A59, Ser-A60, Gln-A61, AsnA-92, Leu-A93, Leu-A94, Ser-A95, Tyr-B119, Leu-B120, Gly-B121, Tyr-B151	3	Ser-A60, AsnA-92, Leu-B120
Gymnemic acid XIII	−7.2	Leu-A57, Tyr-A59, Ser-A95, Ala-A96, Tyr-B119, Leu-B120, Gly-B121, Tyr-B151	1	Tyr-B151
Gymnemic acid XIV	−7.2	Leu-A57, Tyr-A59, Gln-A61, Ala-A96, Tyr-B119, Leu-B120, Gly-B121, Tyr-B151	1	Tyr-B151

Tumor necrosis factor-α (TNF-α) is a cytokine primarily secreted by macrophages in response to inflammatory stimuli, septic shock, and cachexia [88]. It plays a pivotal role in both the immune response and cell death mechanisms, such as apoptosis and necrosis [89]. TNF-α is implicated in the pathogenesis of various autoimmune diseases, and is involved in the progression of inflammatory, edematous, neovascular, and neurodegenerative diseases [[88], [89], [90]]. Given its significant role as a mediator in infections and malignancies, a range of biological agents targeting TNF-α have been developed for the treatment of cancer and autoimmune disorders [101]. Several TNF-α antagonists, such as infliximab, etanercept, adalimumab, certolizumab, and golimumab, have been developed for therapeutic interventions. However, these therapies have inherent limitations, including an increased risk of infections, high treatment costs, and the necessity for intravenous administration [102]. Therefore, the development of biological inhibitors targeting TNF-α remains a critical area of research. Our study explores how *G. sylvestre* compounds can inhibit TNF-α. We've found valuable insights into how these compounds interact with TNF-α. This could lead to new treatments for inflammatory and autoimmune diseases. Understanding these interactions helps us advance research in fighting these conditions. Our study paves the way for advancing this field of research.

**Fig. 11:**
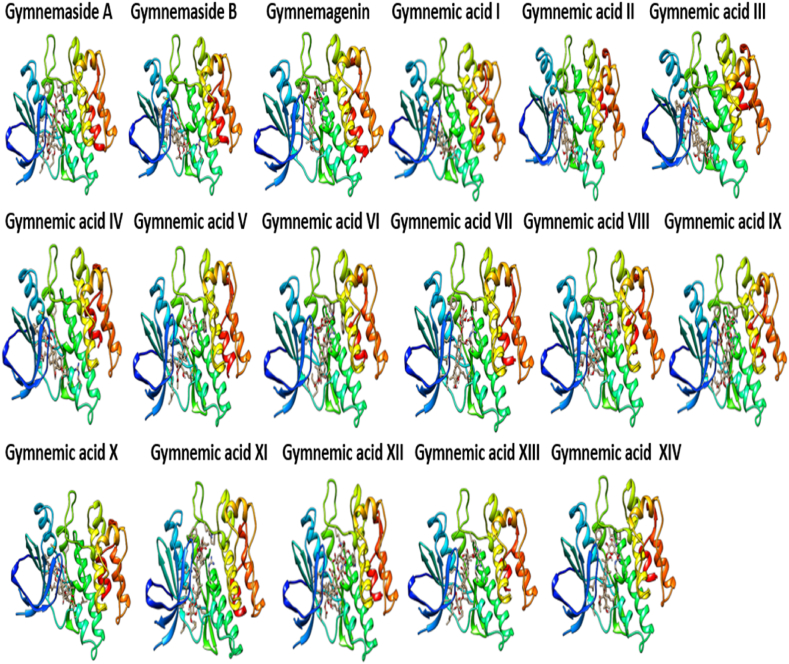
Unveiling the molecular interactions between Gymnemic Acids and EGFR: 3D confirmations of ligand-protein interactions.

Molecular docking analysis was performed to find out the binding affinity of gymnemic acids with breast cancer proteins EGFR and HER2. 3D representations illustrating the interactions between various gymnemic acids (ligands) and EGFR (protein) were depicted in Fig. 11. 2D model of the gymnemic acids-EGFR complex, highlighting active site residues and binding interactions, is available in the supplementary file (Fig. 2S). The results of the molecular docking studies between different gymnemic acids and HER1/EGFR protein are summarized in Table .3. These compounds were bound at the cleft of the enzyme catalytic site by interactions with polar residues such as Asp-B831, Thr-B830, Arg-B817, Cys-B773, Met-B769, Thr-B766, Cys-B751, Lys-B721 and Ser-A696. Gymnemic acid XIII demonstrates a strong binding affinity to EGFR/HER 1 with a binding energy of −8.8 kcal/mol. It engages in three hydrogen bonds with specific residues, including Ala-A698 and Met-B769 and it forms electrostatic interactions with other active site residues. Gymnemagenin exhibits a binding energy of −8.6 kcal/mol and forms a single hydrogen bond with Asp-B813. Gymnemaside B shows a binding energy of −8.4 kcal/mol and forms six hydrogen bonds with residues Cys-B751, Cys-B773, Thr-B766, Thr-B830, and Phe-B832. Gymnemic acid VII exhibits a binding energy of −8.1 kcal/mol and interacts with active site residues through pi-pi interactions. Gymnemaside A exhibits a binding energy of −8.0 kcal/mol. It forms six hydrogen bonds with specific residues: Met-B769, Met-B769, Gln-B767, Cys-B773, Thr-B766, and Thr-B830. Gymnemic acid IV has a binding energy of −7.7 kcal/mol. It forms five hydrogen bonds with the following residues: Thr-B766, Thr-B830, Thr-B830, Thr-B830, and Asp-B83. Gymnemic acid X demonstrates a binding energy of −7.6 kcal/mol. It forms seven hydrogen bonds with specific residues: Leu-A694, Thr-B766, Thr-B830, Thr-B830, Thr-B830, Asp-831, and Phe-832. Gymnemic acid XI has a binding energy of −7.5 kcal/mol and forms four hydrogen bonds with the following residues: Ala-A698, Thr-B766, Gln-B767, and Met-B769. Gymnemic acid III exhibits a binding energy of −7.2 kcal/mol. It forms six hydrogen bonds with specific residues: Leu-A694, Thr-B766, Thr-B830, Thr-B830, Thr-B830, and Asp-B831. Gymnemic acid I and II both exhibit a binding energy of −7.0 kcal/mol, forming six hydrogen bonds with residues like Leu-A694, Thr-B766, and Thr-B830. Gymnemic acid V has a binding energy of −6.9 kcal/mol with 1 hydrogen bond. Gymnemic acid VI shows a binding energy of −6.5 kcal/mol with 4 hydrogen bonds. Gymnemic acid XIV exhibits a binding energy of −6.6 kcal/mol with 4 hydrogen bonds. Gymnemic acid VIII has a binding energy of −6.1 kcal/mol with 6 hydrogen bonds. Gymnemic acid IX demonstrates a binding energy of −5.5 kcal/mol with 3 hydrogen bonds. Lastly, Gymnemic acid XII shows the lowest binding affinity with a binding energy of −5.0 kcal/mol and 1 hydrogen bond. The epidermal growth factor receptor (EGFR; ErbB-1; HER1 in humans) is a transmembrane protein of family receptor tyrosine kinases (RTKs). EGFR is a key factor in malignancies and its activity enhances tumor growth, invasion and metastasis [103]. In cancer cells, EGFR is perpetually stimulated due to sustainable production of EGFR ligands in the tumor microenvironment. Aberrant expression of EGFR by tumors typically confers a more aggressive phenotype and is thus often predictive of poor prognosis. Hence EGFR has emerged as a principal target for therapeutic intervention [104]. *In silico* screening revealed 15 good binding lead compounds in this study which selectively inhibit the HER1 or EGFR. Hence, these lead molecules will be useful for designing selective EGFR inhibitors.

**Table 3:** Binding energies, No. of H-bonds, Active site residues and H-bond interactions between triterpenoid saponins (Ligand) and EGFR/HER 1 (Protein).

Compound name	ΔG (kcal/mol)	No of H bonds	Active site residues	H Bond Residues
Gymnemaside A	−8.0	6	Ser-A696, Gly-A697, Gly-A695, Val-A702, Gly-B772, Leu-A694, Cys-B773, Met-B769, Leu-B768, Gln-B767, Thr-B766, Ala-B719, Thr-B830, Leu-B820, Lys-B721, Asp-B831, Arg-B817, Asp-B813, Lys-B851, Pro-B853, Val-B852, Phe-A699	Met-B769, Met-B769, Gln-B767, Cys-B773, Thr-B766, Thr-B830
Gymnemaside B	−8.4	6	Met-B742, Phe-B832, Leu-B753, Leu-B764, Leu-B834, Lys-B721, Arg-B817, Arg-B779, Asp-B776, Cys-B773, Leu-A694, Gly-A695, Val-A702, Gly-B772, Leu-B820, Ala-B719, Asp-B831, Thr-B766, Thr-B830, Ile-B829, Cys-B751	Cys-B751, Cys-B773, Thr-B766, Thr-B830, Thr-B830, Phe-B832
Gymnemagenin	−8.6	1	Lys-B851, Asn-B818, Arg-B817, Ser-A696, Gly-A695, Cys-B773, Val-A702, Leu-B820, Leu-A694, Asp-B831, Lys-B721, Phe-A699, Pro-B853, Asp-B813	Asp-B813
Gymnemic acid I	−7.0	6	Leu-A694, Cys-B773, Gly-A695, Val-A702, Leu-B820, Ala-B719, Thr-B766, Leu-B834, Leu-B753, Leu-B764, Met-B742, Lys-B721, Phe-B832, Cys-B751, Thr-B830, Asp-B831, Ile-B829, Arg-B817, Asp-B776, Arg-B779	Leu-A694, Thr-B766, Thr-B830, Thr-B830, Thr-B830, Asp-B831
Gymnemic acid II	−6.8	6	Leu-A694, Cys-B773, Gly-A695, Val-A702, Lys-B721, Leu-B820, Ala-B719, Thr-B766, Leu-B834, Leu-B753, Leu-B764, Met-B742, Phe-B832, Cys-B751, Thr-B830, Asp-B831, Ile-B829, Arg-B817, Asp-B776, Arg-B779	Leu-A694, Thr-B766, Thr-B830, Thr-B830, Thr-B830, Asp-B831
Gymnemic acid III	−7.2	6	Leu-A694, Gly-A695, Val-A702, Leu-B820, Ala-B719, Lys-B721, Thr-B766, Leu-B834, Ile-B829, Leu-B753, Met-B742, Phe-B832, Cys-B751, Leu-B764, Thr-B830, Asp-B831, Arg-B817, Asp-B776, Arg-B779, Cys-B773	Leu-A694, Thr-B766, Thr-B830, Thr-B830, Thr-B830, Asp-B831
Gymnemic acid IV	−7.7	5	Cys-B773, Gly-A695, Val-A702, Leu-A694, Leu-B820, Ala-B719, Lys-B721, Thr-B766, Leu-B834, Leu-B753, Met-B742, Phe-B832, Cys-B751, Leu-B764, Thr-B830, Asp-B831, Ile-B829, Arg-B817, Asp-B776, Arg-B779, Leu-B775	Thr-B766, Thr-B830, Thr-B830, Thr-B830, Asp-B831
Gymnemic acid V	−6.9	1	Gly-A697, Ser-A696, Cys-B773, Gly-A695, Leu-B820, Met-B769, Leu-B768, Thr-B830, Val-A702, Leu-B764, Gln-B767, Lys-B721, Ala-B719, Thr-B766, Asp-B831, Ile-B720, Leu-A694, Arg-B817, Lys-B851, Trp-B856	Thr-B830
Gymnemic acid VI	−6.5	4	Ser-A696, Gly-A697, Ala-A698, Phe-A699, Gly-A700, Lys-B721, Asn-B818, Asp-B813, Arg-B817, Lys-B851, Pro-B853, Trp-B856, Val-B852, Asp-B776, Val-A702, Leu-A694, Met-B769, Leu-B820, Gly-B772, Cys-B773	Lys-B721, Arg-B817, Arg-B817, Val-B852
Gymnemic acid VII	−8.1	0	Gly-B772, Leu-A694, Cys-B773, Val-A702, Gly-A695, Ser-A696, Gly-A697, Arg-B817, Lys-B851, Pro-B853, Phe-A699, Leu-B834, Asp-B813, Leu-B838, Ala-B835, Lys-B721, Asp-B831, Leu-B820, Ala-B719, Met-B769	
Gymnemic acid VIII	−6.1	6	Leu-B820, Thr-B766, Ala-B719, Thr-B830, Asp-B831, Val-B852, Asn-B818, Gly-A700, Lys-B721, Asp-B813, Phe-A699, Gly-A697, Arg-B817, Lys-B851, Trp-B856, Ala-A698, Ser-A696, Pro-B853, Gly-A695, Leu-A694, Val-A702	Ser-A696, Lys-B721, Thr-B766, Asp-B813, Arg-B817, Arg-B817
Gymnemic acid IX	−5.5	3	Val-A702, Leu-A694, Ala-B719, Met-B769, Gly-B772, Leu-B820, Cys-B773, Asp-B776, Pro-B853, Val-B852, Lys-B851, Lys-B721, Phe-A699, Gly-A697, Ala-A698, Arg-B817, Ser-A696	Ala-A698, Lys-B721, Lys-B851
Gymnemic acid X	−7.6	7	Leu-B753, Thr-B766, Ala-B719, Lys-B721, Leu-B775, Leu-B820, Val-A702, Cys-B773, Asp-B776, Leu-A694, Gly-A695, Ser-A696, Ile-B829, Arg-B817, Asp-B831, Leu-B764, Phe-B832, Thr-B830	Leu-A694, Thr-B766, Thr-B830, Thr-830, Thr-830, Asp-831, Phe-832
Gymnemic acid XI	−7.5	4	Leu-A694, Ala-B719, Thr-C830, Leu-C820, Asp-C831, Val-A702, Ser-A696, Lys-B721, Arg-C817, Ala-A698, Leu-C838, Pro-C853, Phe-A699, Gly-A697, Lys-C851, Cys-B773, Gly-B772, Met-B769, Leu-B768, Gln-B767, Thr-B766	Ala-A698, Thr-B766, Gln-B767, Met-B769
Gymnemic acid XII	−5.0	1	Lys-B851, Pro-B853, Val-B852, Trp-B856, Arg-B817, Asp-B831, Thr-B830, Lys-B721, Leu-B764, Thr-B766, Leu-A694, Leu-B768, Ala-B719, Met-B769, Gly-A695, Val-A702, Leu-B820, Cys-B773, Ser-A696, Leu-B775	Val-B852
Gymnemic acid XIII	−8.8	3	Lys-B721, Gly-A695, Arg-B817, Leu-A694, Asp-B831, Thr-B830, Leu-B820, Ala-B719, Thr-B766, Leu-B768, Met-B769, Gln-B767, Gly-B772, Cys-B773, Val-A702, Ser-A696, Phe-A699, Gly-A697, Ala-A698, Lys-B851, Pro-B853, Val-B852, Trp-B856	Ala-A698, Met-B769, Met-B769
Gymnemic acid XIV	−6.6	4	Cys-B773, Gly-B772, Met-B769, Leu-B768, Ala-B719, Gln-B767, Leu-A694, Val-A702, Leu-B820, Gly-A697, Gly-A695, Ala-A698, Ser-A696, Arg-B817, Phe-A699, Trp-B856, Lys-B851, Pro-B853, Val-B852	Cys-B773, Cys-B773, Arg-B817, Lys-B851

**Fig. 12:**
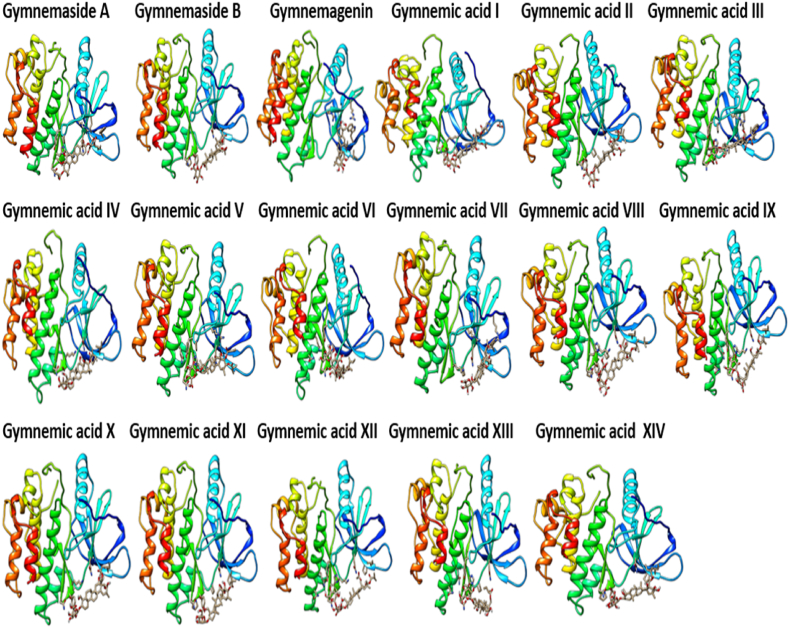
Gymnemic acids (ligands) and HER2 protein: Molecular docking insights from detailed 3D interaction.

Fig. 12 presents detailed 3D representations showcasing the interactions between various gymnemic acids (ligands) and the HER2 protein. Each ligand's unique binding and interaction patterns with HER2 are highlighted, offering insights into their structural relationships and potential pharmacological implications. Additionally, in the supplementary file (Fig. 3S), a 2D model of the gymnemic acids-HER2 complex is provided. This illustration emphasizes the active site residues and the precise binding interactions between the ligands and the HER2 protein, offering a more detailed view of their molecular interactions. The molecular docking studies yielded insightful data, which are summarized in Table 4. Gymnemic acid V exhibits a binding energy of −5.5 kcal/mol and forms a single hydrogen bond with Asn-857. Gymnemic acid IX has a binding energy of −5.4 kcal/mol and forms five hydrogen bonds with residues Gln-799, Gln-828, and Asn-857. Gymnemic acid VII exhibits a binding energy of −5.2 kcal/mol and it forms pi-pi interactions with active site residues. Gymnemic acid XIV forming a singular hydrogen bond with Asn-857 with a binding energy of −5.2 kcal/mol. Gymnemagenin demonstrates a binding energy of −5.2 kcal/mol, forming two H-bond interactions with two residues Arg-713 and Arg-784. Gymnemic acid III has a binding energy of −5.2 kcal/mol and forms hydrogen bonds with four residues: Tyr-781, Asn-857, Asn-857, and His-858. Gymnemic acid VIII exhibits a binding energy of −5.1 kcal/mol and forms hydrogen bonds with three residues: Ser-855, Asn-857, and Asn-857. Gymnemic acid XIII has a binding energy of −5.1 kcal/mol and engages in hydrogen bond interactions with four residues: Tyr-781, Tyr-781, Gln-799, and Gln-828. Gymnemic acid I exhibit a binding energy of −5.0 kcal/mol and forms hydrogen bonds with three residues: Gln-799, Lys-854, and Ser-855. Gymnemic acid II exhibits a binding energy of −5.0 kcal/mol and forms hydrogen bonds with two residues: Ser-855 and Asn-857. Gymnemic acid IV exhibits a binding energy of −5.0 kcal/mol and forms hydrogen bonds with two residues: Gln-799 and Ser-855. Gymnemic acid X exhibits a binding energy of −5.0 kcal/mol and forms hydrogen bonds with two residues: Asn-857 and Asn-857. Gymnemic acid XII exhibits a binding energy of −4.9 kcal/mol and forms hydrogen bonds with four residues: Gln-828, Asn-857, Asn-857, and Asn-857. Gymnemic acid XI exhibits a binding energy of −4.8 kcal/mol and forms hydrogen bonds with three residues: Gln-828, Asn-857, and Asn-857. Gymnemaside A exhibits a binding energy of −4.8 kcal/mol and forms hydrogen bonds with four residues: Arg-784, Arg-784, Arg-784, and Gln-799. Gymnemaside B exhibits a binding energy of −4.8 kcal/mol and forms hydrogen bonds with three residues: Tyr-803, Ser-855, and Asn-857. Gymnemic acid VI exhibits a binding energy of −4.7 kcal/mol and forms hydrogen bonds with four residues: Gln-828, Gln-828, Lys-854, and Asn-857. These compounds predominantly interacted with polar residues such as His858, Asn857, Ser855, Lys854, Gln828, Tyr803, Gln799, Arg784, Tyr781, and Arg713, positioning themselves at the cleft of the enzyme's catalytic site. HER2, a member of the human epidermal growth factor receptor (HER/EGFR/ERBB) family of receptor tyrosine protein kinases (RTKs), is also referred to as CD340 or Proto-oncogene Neu [105]. Normally present on the surface of breast cells, HER2 can become amplified or overexpressed in approximately 30 % of breast cancers. Elevated levels of HER2 can stimulate breast cell growth and division, contributing to the onset and progression of breast cancer [106]. HER2 inhibitors have emerged as effective targeted therapies that have revolutionized the treatment of HER2-positive breast cancer, leading to improved outcomes and survival for patients [107]. Currently, several drugs specifically targeting HER2-positive breast cancer, such as Herceptin, Kadcyla, Perjeta, and Tykerb, are available. These drugs function by inhibiting HER2, thereby impeding the growth and proliferation of HER2-positive breast cancer cells [108]. In this study, the strong binding affinities exhibited by the triterpenoid saponins suggest their potential therapeutic utility in combating HER2-positive breast cancer. The insights gained from this study may facilitate the design and synthesis of more potent and selective HER2 inhibitors derived from natural sources, offering new avenues for therapeutic intervention in HER2-positive breast cancer.

**Table 4:** Binding energies, No. of H-bonds, Active site residues and H-bond interactions between triterpenoid saponins (Ligand) and HER 2 (Protein).

Compound name	ΔG (kcal/mol)	No of H bonds	Active site residues	H Bond Residues
Gymnemaside A	−4.8	4	Arg-784, Leu-786, Trp-739, Gln-799, Lys-854, Pro-780, Tyr-781, His-858, Asn-857	Arg-784, Arg-784, Arg-784, Gln-799
Gymnemaside B	−4.8	3	Lys-747, Ile-748, Pro-749, Gln-799, Pro-802, Tyr-803, Lys-854, Ser-855, Pro-856, Asn-857	Tyr-803, Ser-855, Asn-857
Gymnemagenin	−5.2	2	Val-746, Lys-747, Pro-749, Ile-748, Trp-739, Arg-713, Arg-784, Leu-786	Arg-713, Arg-784
Gymnemic acid I	−5.0	3	Val-746, Lys-747, Ile-748, Pro-749, Gln-799, Pro-802, Tyr-803, Lys-854, Ser-855, Pro-856, Asn-857	Gln-799, Lys-854, Ser-855
Gymnemic acid II	−5.0	2	Val-746, Lys-747, Ile-748, Pro-749, Gln-799, Pro-802, Tyr-803, Lys-854, Ser-855, Pro-856, Asn-857	Ser-855, Asn-857
Gymnemic acid III	−5.2	4	Arg-784, Trp-739, Ile-748, Leu-786, Gln-799, Lys-854, His-858, Ser-855, Asn-857, Pro-780, Tyr-781	Tyr-781, Asn-857, Asn-857, His-858
Gymnemic acid IV	−5.0	2	Pro-749, Ile-748, Gln-799, Pro-802, Leu-800, Lys-854, Ser-855, Asn-857	Gln-799, Ser-855
Gymnemic acid V	−5.5	1	Val-746, Ile-748, Arg-784, Trp-739, Leu-786, Gln-799, Lys-854, Tyr-781, Pro-780, Asn-857, His-858	Asn-857
Gymnemic acid VI	−4.7	4	Tyr-781, Asn-857, Gln-828, His-858, Asn-824, Ser-855, Lys-854, Pro-802, Pro-749, Gln-799, Leu-800	Gln-828, Gln-828, Lys-854, Asn-857
Gymnemic acid VII	−5.2	0	Lys-854, Pro-802, Pro-749, Gln-799, Ile-748, Leu-786, Trp-739, Arg-713, Arg-784	
Gymnemic acid VIII	−5.1	3	Val-746, Lys-747, Ile-748, Pro-749, Gln-799, Pro-802, Tyr-803, Asn-857, His-858, Pro-856, Ser-855, Lys-854	Ser-855, Asn-857, Asn-857
Gymnemic acid IX	−5.4	5	Ile-748, Pro-749, Gln-799, Pro-802, Tyr-781, Gln-828 Asn-824, His-858, Asn-857, Ser-855, Lys-854	Gln-799, Gln-828, Asn-857, Asn-857, Asn-857
Gymnemic acid X	−5.0	2	Ile-748, Pro-749, Gln-799, Pro-802, Tyr-803, Pro-856, Asn-857, Ser-855, Lys-854	Asn-857, Asn-857
Gymnemic acid XI	−4.8	3	Ile-748, Pro-749, Gln-799, Leu-800, Pro-802, Tyr-803, Pro-856, Asn-857, Ser-855, Lys-854	Gln-828, Asn-857, Asn-857
Gymnemic acid XII	−4.9	4	Val-746, Lys-747, Ile-748, Pro-749, Gln-799, Leu-800, Pro-802, Tyr-781, His-858, Gln-828, Asn-857, Ser-855, Lys-854	Gln-828, Asn-857, Asn-857, Asn-857
Gymnemic acid XIII	−5.1	4	Gln-799, Leu-800, Pro-802, Tyr-781, His-858, Gln-828, Asn-824, Asn-857, Ser-855, Lys-854	Tyr-781, Tyr-781, Gln-799, Gln-828
Gymnemic acid XIV	−5.2	1	Lys-747, Ile-748, Pro-749, Gln-799, Tyr-803, Lys-854, Ser-855, Pro-856, Asn-857	Asn-857

The current investigation provides comprehensive insights into the potential therapeutic applications of triterpenoid saponins from *G. sylvestre* in targeting inflammatory mediators like TNF-α and breast cancer-associated proteins HER1 and HER2. The binding interactions between *G. sylvestre* compounds and TNF-α, HER1, and HER2, as revealed by molecular docking, highlight the potential of these natural compounds in developing novel inhibitors targeting these proteins. The identification of key amino acid residues involved in the binding mechanisms further supports the specificity and selectivity of these compounds towards their respective targets. TNF-α, HER1, and HER2 play crucial roles in various pathological conditions, including inflammation, autoimmune diseases, and cancer. Current therapeutic agents targeting these proteins, such as TNF-α antagonists and HER2 inhibitors, have limitations, including side effects, high costs, and the necessity for intravenous administration. Therefore, the development of novel, effective, and safe inhibitors targeting these proteins is of utmost importance. The insights gained from this study may facilitate the design and synthesis of more potent, selective, and safe inhibitors derived from natural sources, offering new avenues for therapeutic intervention in TNF-α-mediated inflammatory diseases and HER1-and HER2-driven malignancies. Further research is warranted to validate the therapeutic potential of these compounds and to explore their application in the treatment of inflammatory and autoimmune diseases, as well as HER2-positive breast cancer.

### 3.9 Prediction of physicochemical descriptors, pharmacokinetics, drug-likeness, medicinal chemistry and ADMET properties of gymnemic acids

**Fig. 13:**
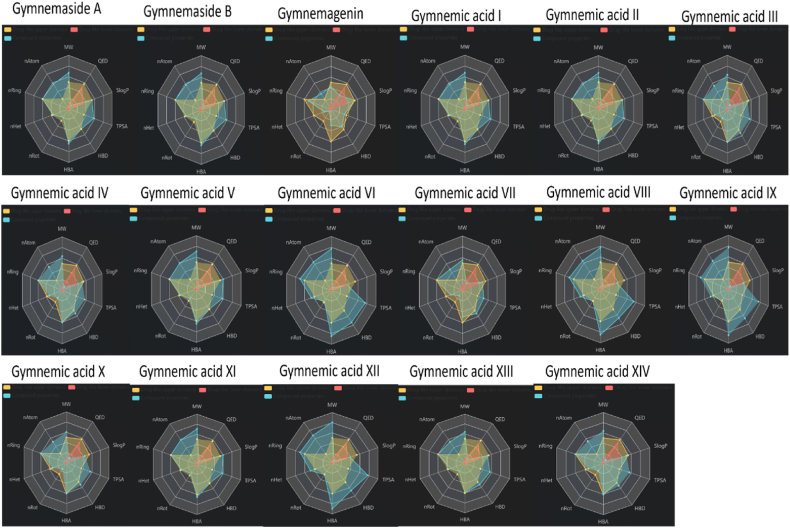
Spider web graphs Depicting compound properties and Drug likeness domains of triterpenoid saponins of *Gymnema Sylvestre*.

Physicochemical descriptors, pharmacokinetics, drug-likeness and medicinal chemistry properties of triterpenoid saponins were represented in Table 5A, Table 5BA–B. Spider graphs depicting physicochemical descriptors, drug likeness properties and drug like domains of gymnemic acids were represented in Fig. 13. The compounds such as Gymnemagenin, Gymnemaside A, Gymnemaside B, and Gymnemic acids I to XIV exhibit molecular weights ranging from 666.84 to 969.12 g/mol. Although this might impact their drug-likeness, with appropriate optimization, they could still be tailored for specific therapeutic applications. The compounds exhibit consensus Log Po/w values ranging from 0.81 to 5.61, indicating moderate to high lipophilicity. This property could be advantageous for targeting specific biological pathways and optimizing drug delivery. While most of the compounds demonstrate moderate solubility, Gymnemagenin, Gymnemic acid VII, and XI show good solubility. Gymnemagenin demonstrates high GI absorption, potentially leading to better oral bioavailability. All compounds are P-gp substrates and do not inhibit major cytochrome P450 enzymes. This suggests they might not have significant drug-drug interactions, simplifying potential co-administration with other medications. Additionally, none of the compounds permeate the blood-brain barrier, reducing the likelihood of central nervous system side effects. The compounds generally exhibit low to moderate skin permeation, which may limit their use in transdermal formulations but could be advantageous for targeted topical applications. Most of the compounds violate Lipinski, Ghose, and Veber rules, pointing to potential drug-likeness issues. Nonetheless, they adhere to Egan's criteria, suggesting they might achieve better oral bioavailability with further optimization. None of the compounds exhibit PAINS alerts, which is favourable. Given their natural origin and known therapeutic effects, Gymnemaside A, Gymnemaside B, Gymnemagenin, and Gymnemic acids I-V, as well as Gymnemic acid VI through XIV, hold promise as drug candidates or ingredients for herbal formulations. Gymnemagenin and Gymnemic acid VII and XI, with their higher solubility and better GI absorption, stand out as potential lead compounds.

**Table 5A:** Predicted Physicochemical, Pharmacokinetic, Druglikeness, and Medicinal Chemistry Properties of Triterpenoid Saponins from *G. sylvestre* using SwissADME Tool.

SwissADME	Gymnemaside A	Gymnemaside B	Gymnemagenin	Gymnemic acid I	Gymnemic acid II	Gymnemic acid III	Gymnemic acid IV	Gymnemic acid V
**Physicochemical Properties**								
Formula	C43H66O14	C43H66O14	C30H50O6	C43H66O14	C43H68O14	C41H66O13	C41H64O13	C46H70O14
Molecular weight	806.98 g/mol	806.98 g/mol	506.71 g/mol	806.98 g/mol	808.99 g/mol	766.95 g/mol	764.94 g/mol	847.04 g/mol
Num. heavy atoms	57	57	36	57	57	54	54	60
Num. arom. heavy atoms	0	0	0	0	0	0	0	0
Fraction Csp3	0.84	0.84	0.93	0.84	0.88	0.90	0.85	0.80
Num. rotatable bonds	10	10	2	10	11	9	8	11
Num. H-bond acceptors	14	14	6	14	14	13	13	14
Num. H-bond donors	7	7	6	7	7	8	8	7
Molar Refractivity	207.11	207.11	140.69	207.11	207.58	197.84	197.37	221.05
TPSA	229.74 Å^2^	229.74 Å^2^	121.38 Å^2^	229.74 Å^2^	229.74 Å^2^	223.67 Å^2^	223.67 Å^2^	229.74 Å^2^
**Lipophilicity**								
Log *P*_o/w_ (iLOGP)	4.47	4.47	3.32	4.49	5.29	3.58	3.45	4.58
Log *P*_o/w_ (XLOGP3)	4.41	4.41	3.97	3.86	4.06	4.04	3.84	5.61
Log *P*_o/w_ (WLOGP)	3.03	3.03	3.03	3.03	3.11	2.54	2.46	3.98
Log *P*_o/w_ (MLOGP)	1.25	1.25	2.62	1.25	1.34	1.02	0.93	1.66
Log *P*_o/w_ (SILICOS-IT)	2.55	2.55	3.03	2.55	2.71	2.16	2.00	3.49
Consensus Log *P*_o/w_	3.14	3.07	3.19	3.04	3.30	2.67	2.54	3.86
**Water Solubility**								
Log *S* (ESOL)	−6.96	−6.96	−5.35	−6.62	−6.69	−6.55	−6.47	−7.90
Solubility	8.82e-05 mg/ml; 1.09e-07 mol/l	8.82e-05 mg/ml; 1.09e-07 mol/l	2.26e-03 mg/ml; 4.46e-06 mol/l	1.96e-04 mg/ml; 2.43e-07 mol/l	1.66e-04 mg/ml; 2.05e-07 mol/l	2.18e-04 mg/ml; 2.84e-07 mol/l	2.57e-04 mg/ml; 3.36e-07 mol/l	1.07e-05 mg/ml; 1.26e-08 mol/l
Class	Poorly soluble	Poorly soluble	Moderately soluble	Poorly soluble	Poorly soluble	Poorly soluble	Poorly soluble	Poorly soluble
Log *S* (Ali)	−8.95	−8.95	−6.22	−8.38	−8.59	−8.44	−8.23	−10.20
Solubility	9.01e-07 mg/ml; 1.12e-09 mol/l	9.01e-07 mg/ml; 1.12e-09 mol/l	3.05e-04 mg/ml; 6.03e-07 mol/l	3.35e-06 mg/ml; 4.16e-09 mol/l	2.09e-06 mg/ml; 2.58e-09 mol/l	2.78e-06 mg/ml; 3.63e-09 mol/l	4.47e-06 mg/ml; 5.85e-09 mol/l	5.38e-08 mg/ml; 6.35e-11 mol/l
Class	Poorly soluble	Poorly soluble	Poorly soluble	Poorly soluble	Poorly soluble	Poorly soluble	Poorly soluble	Insoluble
Log *S* (SILICOS-IT)	−2.85	−2.85	−3.55	−2.85	−3.21	−2.62	−2.26	−3.26
Solubility	1.15e+00 mg/ml; 1.43e-03 mol/l	1.15e+00 mg/ml; 1.43e-03 mol/l	1.43e-01 mg/ml; 2.82e-04 mol/l	1.15e+00 mg/ml; 1.43e-03 mol/l	5.03e-01 mg/ml; 6.22e-04 mol/l	1.83e+00 mg/ml; 2.39e-03 mol/l	4.18e+00 mg/ml; 5.47e-03 mol/l	4.69e-01 mg/ml; 5.53e-04 mol/l
Class	Soluble	Soluble	Soluble	Soluble	Soluble	Soluble	Soluble	Soluble
**Pharmacokinetics**								
GI absorption	Low	Low	High	Low	Low	Low	Low	Low
BBB permeant	No	No	No	No	No	No	No	No
P-gp substrate	Yes	Yes	Yes	Yes	Yes	Yes	Yes	Yes
CYP1A2 inhibitor	No	No	No	No	No	No	No	No
CYP2C19 inhibitor	No	No	No	No	No	No	No	No
CYP2C9 inhibitor	No	No	No	No	No	No	No	No
CYP2D6 inhibitor	No	No	No	No	No	No	No	No
CYP3A4 inhibitor	No	No	No	No	No	No	No	No
Log *K*_p_ (skin permeation)	−8.09 cm/s	−8.09 cm/s	−6.57 cm/s	−8.48 cm/s	−8.35 cm/s	−8.11 cm/s	−8.24 cm/s	−7.48 cm/s
**Drug likeness**								
Lipinski	No; 3 violations: MW > 500, NorO>10 NHorOH>5	No; 3 violations: MW > 500, NorO>10 NHorOH>5	No; 2 violations: MW > 500, NHorOH>5	No; 3 violations: MW > 500, NorO>10 NHorOH>5	No; 3 violations: MW > 500, NorO>10 NHorOH>5	No; 3 violations: MW > 500, NorO>10 NHorOH>5	No; 3 violations: MW > 500, NorO>10 NHorOH>5	No; 3 violations: MW > 500, NorO>10 NHorOH>5
Ghose	No; 3 violations: MW > 480, MR > 130 #atoms>70	No; 3 violations: MW > 480, MR > 130 #atoms>70	No; 3 violations: MW > 480, MR > 130 #atoms>70	No; 3 violations: MW > 480, MR > 130 #atoms>70	No; 3 violations: MW > 480, MR > 130 #atoms>70	No; 3 violations: MW > 480, MR > 130 #atoms>70	No; 3 violations: MW > 480, MR > 130 #atoms>70	No; 3 violations: MW > 480, MR > 130 #atoms>70
Veber	No; 1 violation: TPSA>140	No; 1 violation: TPSA>140	yes	No; 1 violation: TPSA>140	No; 2 violations: Rotors>10 TPSA>140	No; 1 violation TPSA>140	No; 1 violation TPSA>140	No; 2 violations: Rotors>10 TPSA>140
Egan	No; 1 violation: TPSA>131.6	No; 1 violation: TPSA>131.6	yes	No; 1 violation: TPSA>131.6	No; 1 violation: TPSA>131.6	No; 1 violation: TPSA>131.6	No; 1 violation: TPSA>131.6	No; 1 violation: TPSA>131.6
Muegge	No; 4 violations MW > 600, TPSA>150, H-acc>10, H-don>5	No; 4 violations MW > 600, TPSA>150, H-acc>10, H-don>5	No; 1 violation H-don>5	No; 4 violations MW > 600, TPSA>150, H-acc>10, H-don>5	No; 4 violations MW > 600, TPSA>150, H-acc>10, H-don>5	No; 4 violations MW > 600, TPSA>150, H-acc>10, H-don>5	No; 4 violations MW > 600, TPSA>150, H-acc>10, H-don>5	No; 5 violations MW > 600, XLOGP3>5, TPSA>150, H-acc>10, H-don>5
Bioavailability Score	0.11	0.11	0.17	0.11	0.11	0.11	0.11	0.11
**Medicinal Chemistry**								
PAINS	0 alert	0 alert	0 alert	0 alert	0 alert	0 alert	0 alert	0 alert
Brenk	4 alerts: isolated alkene Michael acceptor 1 more than 2 esters, saponine derivative	4 alerts: isolated alkene Michael acceptor 1 more than 2 esters, saponine derivative	1 alert: isolated alkene	4 alerts: isolated alkene Michael acceptor 1 more than 2 esters, saponine derivative	3 alerts: isolated alkene more than 2 esters, saponine derivative	2 alerts: isolated alkene, saponine derivative	3 alerts: isolated alkene more than 2 esters, saponine derivative	4 alerts: isolated alkene Michael acceptor 1 more than 2 esters, saponine derivative
Leadlikeness	No; 3 violations: MW > 350, Rotors>7, XLOGP3>3.5	No; 3 violations: MW > 350, Rotors>7, XLOGP3>3.5	No; 2 violations: MW > 350, XLOGP3>3.5	No; 3 violations: MW > 350, Rotors>7, XLOGP3>3.5	No; 3 violations: MW > 350, Rotors>7, XLOGP3>3.5	No; 3 violations: MW > 350, Rotors>7, XLOGP3>3.5	No; 3 violations: MW > 350, Rotors>7, XLOGP3>3.5	No; 3 violations: MW > 350, Rotors>7, XLOGP3>3.5
Synthetic accessibility	8.82	8.81	6.82	8.81	8.89	8.68	8.60	9.13

**Table 5B:** Predicted Physicochemical, Pharmacokinetic, Druglikeness, and Medicinal Chemistry Properties of Triterpenoid Saponins from *G. sylvestre* using SwissADME Tool.

SwissADME	Gymnemic acid VI	Gymnemic acid VII	Gymnemic acid VIII	Gymnemic acid IX	Gymnemic acid X	Gymnemic acid XI	Gymnemic acid XII	Gymnemic acid XIII	Gymnemic acid XIV
**Physicochemical Properties**									
Formula	C47H74O18	C36H58O11	C47H74O18	C47H72O18	C38H60O13	C46H70O14	C49H76O19	C41H66O13	C41H64O13
Molecular weight	927.08 g/mol	666.84 g/mol	927.08 g/mol	925.06 g/mol	724.88 g/mol	847.04 g/mol	969.12 g/mol	766.95 g/mol	764.94 g/mol
Num. heavy atoms	65	47	65	65	51	60	68	54	54
Num. arom. heavy atoms	0	0	0	0	0	0	0	0	0
Fraction Csp3	0.87	0.92	0.89	0.85	0.89	0.80	0.86	0.90	0.85
Num. rotatable bonds	11	5	12	11	7	11	13	9	8
Num. H-bond acceptors	18	11	18	18	13	14	19	13	13
Num. H-bond donors	11	8	10	10	8	7	10	8	8
Molar Refractivity	229.75	172.52	229.26	228.79	183.42	221.05	239.49	197.84	197.37
TPSA	302.82 Å^2^	197.37 Å^2^	299.66 Å^2^	299.66 Å^2^	223.67 Å^2^	229.74 Å^2^	308.89 Å^2^	223.67 Å^2^	223.67 Å^2^
**Lipophilicity**									
Log *P*_o/w_ (iLOGP)	2.93	3.06	2.99	2.32	3.89	5.02	4.64	3.44	3.38
Log *P*_o/w_ (XLOGP3)	2.25	3.04	2.88	2.67	2.09	5.06	2.27	3.49	3.29
Log *P*_o/w_ (WLOGP)	0.28	1.97	0.57	0.49	1.51	3.98	0.85	2.54	2.46
Log *P*_o/w_ (MLOGP)	−1.34	0.94	−1.34	−1.43	0.51	1.66	−1.02	1.02	0.93
Log *P*_o/w_ (SILICOS-IT)	−0.05	1.41	0.70	0.54	1.07	3.49	0.51	2.16	2.00
Consensus Log *P*_o/w_	0.81	2.08	1.16	0.92	1.81	3.84	1.45	2.53	2.41
**Water Solubility**									
Log *S* (ESOL)	−6.28	−5.56	−6.61	−6.53	−5.19	−7.55	−6.42	−6.20	−6.13
Solubility	4.87e-04 mg/ml; 5.26e-07 mol/l	1.84e-03 mg/ml; 2.76e-06 mol/l	2.27e-04 mg/ml; 2.45e-07 mol/l	2.72e-04 mg/ml; 2.94e-07 mol/l	4.69e-03 mg/ml; 6.47e-06 mol/l	2.37e-05 mg/ml; 2.80e-08 mol/l	3.68e-04 mg/ml; 3.80e-07 mol/l	4.84e-04 mg/ml; 6.31e-07 mol/l	5.71e-04 mg/ml; 7.46e-07 mol/l
Class	Poorly soluble	Moderately soluble	Poorly soluble	Poorly soluble	Moderately soluble	Poorly soluble	Poorly soluble	Poorly soluble	Poorly soluble
Log *S* (Ali)	−8.25	−6.85	−8.83	−8.61	−6.42	−9.63	−8.39	−7.87	−7.66
Solubility	5.27e-06 mg/ml; 5.69e-09 mol/l	9.41e-05 mg/ml; 1.41e-07 mol/l	1.36e-06 mg/ml; 1.47e-09 mol/l	2.25e-06 mg/ml; 2.43e-09 mol/l	2.77e-04 mg/ml; 3.83e-07 mol/l	2.00e-07 mg/ml; 2.36e-10 mol/l	3.92e-06 mg/ml; 4.04e-09 mol/l	1.03e-05 mg/ml; 1.35e-08 mol/l	1.66e-05 mg/ml; 2.18e-08 mol/l
Class	Poorly soluble	Poorly soluble	Poorly soluble	Poorly soluble	Poorly soluble	Poorly soluble	Poorly soluble	Poorly soluble	Poorly soluble
Log *S* (SILICOS-IT)	−0.39	−2.09	−1.45	−1.09	−1.85	−3.26	−0.96	−2.62	−2.26
Solubility	3.79e+02 mg/ml; 4.09e-01 mol/l	5.46e+00 mg/ml; 8.18e-03 mol/l	3.32e+01 mg/ml; 3.58e-02 mol/l	7.59e+01 mg/ml; 8.20e-02 mol/l	1.03e+01 mg/ml; 1.42e-02 mol/l	4.69e-01 mg/ml; 5.53e-04 mol/l	1.05e+02 mg/ml; 1.08e-01 mol/l	1.83e+00 mg/ml; 2.39e-03 mol/l	4.18e+00 mg/ml; 5.47e-03 mol/l
Class	Soluble	Soluble	Soluble	Soluble	Soluble	Soluble	Soluble	Soluble	Soluble
**Pharmacokinetics**									
GI absorption	Low	Low	Low	Low	Low	Low	Low	Low	Low
BBB permeant	No	No	No	No	No	No	No	No	No
P-gp substrate	Yes	Yes	Yes	Yes	Yes	Yes	Yes	Yes	Yes
CYP1A2 inhibitor	No	No	No	No	No	No	No	No	No
CYP2C19 inhibitor	No	No	No	No	No	No	No	No	No
CYP2C9 inhibitor	No	No	No	No	No	No	No	No	No
CYP2D6 inhibitor	No	No	No	No	No	No	No	No	No
CYP3A4 inhibitor	No	No	No	No	No	No	No	No	No
Log *K*_p_ (skin permeation)	−10.36 cm/s	−8.21 cm/s	−9.91 cm/s	−10.05 cm/s	−9.24 cm/s	−7.87 cm/s	−10.60 cm/s	−8.50 cm/s	−8.63 cm/s
**Druglikeness**									
Lipinski	No; 3 violations: MW > 500, NorO>10 NHorOH>5	No; 3 violations: MW > 500, NorO>10 NHorOH>5	No; 3 violations: MW > 500, NorO>10 NHorOH>5	No; 3 violations: MW > 500, NorO>10 NHorOH>5	No; 3 violations: MW > 500, NorO>10 NHorOH>5	No; 3 violations: MW > 500, NorO>10 NHorOH>5	No; 3 violations: MW > 500, NorO>10 NHorOH>5	No; 3 violations: MW > 500, NorO>10 NHorOH>5	No; 3 violations: MW > 500, NorO>10 NHorOH>5
Ghose	No; 3 violations: MW > 480, MR > 130 #atoms>70	No; 3 violations: MW > 480, MR > 130 #atoms>70	No; 3 violations: MW > 480, MR > 130 #atoms>70	No; 3 violations: MW > 480, MR > 130 #atoms>70	No; 3 violations: MW > 480, MR > 130 #atoms>70	No; 3 violations: MW > 480, MR > 130 #atoms>70	No; 3 violations: MW > 480, MR > 130 #atoms>70	No; 3 violations: MW > 480, MR > 130 #atoms>70	No; 3 violations: MW > 480, MR > 130 #atoms>70
Veber	No; 2 violations: Rotors>10, TPSA>140	No; 1 violation: TPSA>140	No; 2 violations: Rotors>10, TPSA>140	No; 2 violations: Rotors>10, TPSA>140	No; 1 violation: TPSA>140	No; 2 violations: Rotors>10, TPSA>140	No; 2 violations: Rotors>10, TPSA>140	No; 1 violation: TPSA>140	No; 1 violation: TPSA>140
Egan	No; 1 violation: TPSA>131.6	No; 1 violation: TPSA>131.6	No; 1 violation: TPSA>131.6	No; 1 violation: TPSA>131.6	No; 1 violation: TPSA>131.6	No; 1 violation: TPSA>131.6	No; 1 violation: TPSA>131.6	No; 1 violation: TPSA>131.6	No; 1 violation: TPSA>131.6
Muegge	No; 4 violations MW > 600, TPSA>150, H-acc>10, H-don>5	No; 4 violations MW > 600, TPSA>150, H-acc>10, H-don>5	No; 4 violations MW > 600, TPSA>150, H-acc>10, H-don>5	No; 4 violations MW > 600, TPSA>150, H-acc>10, H-don>5	No; 4 violations MW > 600, TPSA>150, H-acc>10, H-don>5	No; 5violations MW > 600, XLOGP3>5, TPSA>150, H-acc>10, H-don>5	No; 4 violations MW > 600, TPSA>150, H-acc>10, H-don>5	No; 4 violations MW > 600, TPSA>150, H-acc>10, H-don>5	No; 4 violations MW > 600, TPSA>150, H-acc>10, H-don>5
Bioavailability Score	0.11	0.11	0.11	0.11	0.11	0.11	0.11	0.11	0.11
**Medicinal Chemistry**									
PAINS	0 alert	0 alert	0 alert	0 alert	0 alert	0 alert	0 alert	0 alert	0 alert
Brenk	3 alerts: isolated alkene Michael acceptor 1, saponine derivative	2 alerts: isolated alkene, saponine derivative	3 alerts: isolated alkene, saponine derivative Michael acceptor 1,	3 alerts: isolated alkene, saponine derivative, Michael acceptor 1,	2 alerts: isolated alkene, saponine derivative	4 alerts: isolated alkene Michael acceptor 1, more than 2 esters, saponine derivative	4 alerts: isolated alkene Michael acceptor 1, more than 2 esters, saponine derivative	2 alerts: isolated alkene, saponine derivative	3 alerts: isolated alkene Michael acceptor 1, saponine derivative
Lead likeness	No; 2 violations: MW > 350, Rotors>7	No; 1 violation: MW > 350	No; 2 violations: MW > 350, Rotors>7	No; 2 violations: MW > 350, Rotors>7	No; 1 violation: MW > 350	No; 3 violations: MW > 350, Rotors>7, XLOGP3>3.5	No; 2 violations: MW > 350, Rotors>7	No; 2 violations: MW > 350, Rotors>7	No; 2 violations: MW > 350, Rotors>7
Synthetic accessibility	9.67	8.04	9.62	9.55	8.22	9.12	9.89	8.65	8.57

Absorption, distribution, metabolism, excretion, and toxicity (ADMET) properties of triterpenoid saponins were represented in Table 6A, Table 6BA–B. The compounds under investigation, Gymnemic acid I to XIV, exhibit promising ADMET profiles suggesting potential therapeutic utility. They demonstrate varying solubility and lipophilicity, with logS values ranging from −4.04 to −6.8 and logP values from 1.54 to 4.41. The pKa values indicate differential ionization at physiological pH, with acidic pKa values ranging from 4.39 to 6.12 and basic pKa values from 5.78 to 7.20. All compounds showing intestinal absorption, reflected by Caco-2 values ranging from −5.61 to −7.04, All compounds inhibit OATP1B1 and BSEP, and are substrates for P-glycoprotein, exhibiting high plasma protein binding potential. Regarding metabolism and excretion, all compounds are substrates for UGT and undergo renal clearance, with a half-life ranging from −0.14 to −0.90 and mean retention time from −0.08 to −0.77. All compounds, except Gymnema side A, Gymnemic acid IV, and Gymnemic acid V, are classified as having moderate acute oral toxicity. None of the compounds display endocrine disruption, mutagenicity, carcinogenicity, skin or eye irritation, or nephrotoxicity. Gymnemagenin shows potential binding to aromatase, and Gymnemic acid VIII exhibits potential binding to glucocorticoid receptors.

**Table 6A:** Predicted Absorption, Distribution, Metabolism, Excretion and Toxicity (ADMET), Endocrine Disruption, and Medicinal Chemistry Friendliness Properties of Triterpenoid Saponins from *G. sylvestre* using admetSAR 3.0 Tool.

ADMET Properties	Gymnema side A	Gymnema side B	Gymnema genin	Gymnemic acid I	Gymnemic acid II	Gymnemic acid III	Gymnemic acid IV	Gymnemic acid V	Gymnemic acid VI
**Absorption**									
logS	−4.87	−4.76	−3.88	−4.65	−4.45	−4.57	−4.84	−5.3	−4.53
logP	3.11	2.89	1.9	2.95	2.78	2.45	2.52	4.26	1.82
pKa	5.61	5.36	5.24	5.66	5.42	5.02	5.42	6.12	5.96
Acidic pKa	5.74	5.46	5.65	5.63	5.25	5.07	5.55	6.3	6.07
Basic pKa	7.34	7.36	5.93	7.51	7.78	7.28	7.24	6.88	7.33
Caco-2	−6.78	−6.74	−5.61	−6.68	−6.58	−6.7	−6.8	−6.45	−7.01
HIA	1	0	1	0	0	0	0	1	0
MDCK	0	0	1	0	0	0	0	0	0
F50 %	0	0	0	0	0	0	0	0	0
F30 %	0	0	0	0	0	0	0	0	0
F20 %	0	0	0	0	0	0	0	0	0
**Distribution**									
BBB	0	0	1	0	0	0	0	0	0
OATP1B1 inhibitor	1	0	1	1	1	1	1	0	1
OATP1B3 inhibitor	1	1	1	1	1	1	1	0	1
OATP2B1 inhibitor	0	0	0	0	0	0	0	0	0
OCT1 inhibitor	0	0	0	0	0	0	0	0	0
OCT2 inhibitor	0	0	0	0	0	0	0	0	0
BCRP inhibitor	0	0	0	0	0	0	0	1	0
BSEP inhibitor	1	1	1	1	1	1	1	1	1
MATE1 inhibitor	0	0	0	0	0	0	0	0	0
Pgp inhibitor	0	0	0	0	0	0	0	1	0
Pgp substrate	1	1	1	1	1	1	1	1	1
PPB	1	1	1	1	1	1	1	1	1
VDss	−0.69	−0.74	−0.32	−0.66	−0.76	−0.61	−0.53	−0.74	−0.54
**Metabolism**									
CYP1A2 inhibitor	0	0	0	0	0	0	0	0	0
CYP3A4 inhibitor	0	0	0	0	0	0	0	0	0
CYP2B6 inhibitor	0	0	0	0	0	0	0	0	0
CYP2C9 inhibitor	0	0	0	0	0	0	0	0	0
CYP2C19 inhibitor	0	0	0	0	0	0	0	0	0
CYP2D6 inhibitor	0	0	0	0	0	0	0	0	0
CYP1A2 substrate	0	0	0	0	0	0	0	0	0
CYP3A4 substrate	0	0	0	0	0	0	0	0	0
CYP2B6 substrate	0	0	0	0	0	0	0	0	0
CYP2C9 substrate	0	0	0	0	0	0	0	0	0
CYP2C19 substrate	0	0	0	0	0	0	0	0	0
CYP2D6 substrate	0	0	0	0	0	0	0	0	0
HLM	0	0	0	0	0	0	0	0	0
RLM	0	0	0	0	0	0	0	0	0
UGT substrate	1	1	1	1	1	1	1	1	1
**Excretion**									
Plasma clearance	0	0	0	0	0	0	0	0	0
Renal Clearance	1	0	1	1	1	1	1	1	1
Half-life (T1/2)	−0.36	−0.23	−0.73	−0.44	−0.17	−0.34	−0.64	−0.6	−0.42
Mean retention time	−0.09	−0.07	−0.54	−0.18	−0.1	−0.09	−0.41	−0.32	−0.19
**Toxicity**									
Neurotoxicity	−2.88	−2.79	−2.46	−2.8	−2.76	−2.66	−2.67	−3.01	−2.61
hERG 1uM	0	0	0	0	0	0	0	0	0
Respiratory toxicity	1	1	1	1	1	1	1	1	1
Nephrotoxicity	0	0	0	0	0	0	0	0	0
Eye irritation	0	0	0	0	0	0	0	0	0
Skin irritation	0	0	0	0	0	0	0	0	0
Acute dermal toxicity	1	1	1	1	1	1	1	1	1
Mouse carcinogenicity	0	0	0	0	0	0	0	0	0
Ames mutagenesis	0	0	0	0	0	0	0	0	0
Reproductive toxicity	1	1	1	1	1	1	1	1	1
Hemolytic toxicity	1	1	1	1	1	1	1	1	1
Repeated dose toxicity	0	0	0	0	0	0	0	0	0
Acute oral toxicity	0	0	0	1	0	0	0	1	0
Fish toxicity	1	1	1	1	1	1	1	1	1
Bee toxicity	0	0	0	1	0	0	0	1	0
Biodegradability	0	0	0	0	0	0	0	0	0
**Endocrine disruption**									
Androgen receptor	0	0	0	0	0	0	0	0	0
Estrogen receptor	0	0	0	0	0	0	0	0	0
Aromatase	0	0	1	0	0	0	0	0	0
ARE	0	0	0	0	0	0	0	0	0
HSE	0	0	0	0	0	0	0	0	0
P53	0	0	0	0	0	0	0	0	0
PPARϒ	0	0	0	0	0	0	0	0	0
MMP	0	0	0	0	0	0	0	0	0
TR	0	0	0	0	0	0	0	0	0
GR	0	0	1	0	0	0	0	0	0
**Medicinal chemistry**									
QED	0.08	0.08	0.32	0.08	0.1	0.1	0.08	0.08	0.06
Pfizer rule	Accept	Accept	Accept	Accept	Accept	Accept	Accept	Accept	Accept

**Table 6B:** Predicted Absorption, Distribution, Metabolism, Excretion and Toxicity (ADMET), Endocrine Disruption, and Medicinal Chemistry Friendliness Properties of Triterpenoid Saponins from *G. sylvestre* using admetSAR 3.0 Tool.

ADMET Properties	Gymnemic acid VII	Gymnemic acid VIII	Gymnemic acid IX	Gymnemic acid X	Gymnemic acid XI	Gymnemic acid XII	Gymnemic acid XIII	Gymnemic acid XIV
**Absorption**								
logS	−4.04	−4.45	−4.6	−4.24	−5.46	−4.48	−4.43	−4.56
logP	1.54	1.77	1.86	1.8	4.41	2.21	2.43	2.34
pKa	4.94	5.76	5.89	4.35	6.2	5.53	5.21	5.31
Acidic pKa	5.02	5.79	6.04	4.39	6.12	5.67	5.16	5.42
Basic pKa	6.27	7.16	7.03	5.78	7.04	7.13	7.2	6.99
Caco-2	−6.51	−6.93	−7.04	−6.6	−6.35	−6.85	−6.68	−6.65
HIA	1	0	0	0	0	0	0	0
MDCK	0	0	0	0	0	0	0	0
F50 %	0	0	0	0	0	0	0	0
F30 %	0	0	0	0	0	0	0	0
F20 %	1	0	0	0	0	0	0	0
**Distribution**								
BBB	1	0	0	0	0	0	0	0
OATP1B1 inhibitor	1	1	1	1	0	1	1	1
OATP1B3 inhibitor	1	1	1	1	0	1	1	1
OATP2B1 inhibitor	0	0	0	0	0	0	0	0
OCT1 inhibitor	0	0	0	0	0	0	0	0
OCT2 inhibitor	0	0	0	0	0	0	0	0
BCRP inhibitor	0	0	0	0	0	0	0	0
BSEP inhibitor	1	1	1	1	1	1	1	1
MATE1 inhibitor	0	0	0	0	0	0	0	0
Pgp inhibitor	0	0	0	0	1	0	0	0
Pgp substrate	1	1	1	1	1	1	1	1
PPB	1	1	1	1	1	1	1	1
VDss	−0.42	−0.59	−0.54	−0.56	−0.72	−0.63	−0.56	−0.48
**Metabolism**								
CYP1A2 inhibitor	0	0	0	0	0	0	0	0
CYP3A4 inhibitor	0	0	0	0	0	0	0	0
CYP2B6 inhibitor	0	0	0	0	0	0	0	0
CYP2C9 inhibitor	0	0	0	0	0	0	0	0
CYP2C19 inhibitor	0	0	0	0	0	0	0	0
CYP2D6 inhibitor	0	0	0	0	0	0	0	0
CYP1A2 substrate	0	0	0	0	0	0	0	0
CYP3A4 substrate	0	0	0	0	0	0	0	0
CYP2B6 substrate	0	0	0	0	0	0	0	0
CYP2C9 substrate	0	0	0	0	0	0	0	0
CYP2C19 substrate	0	0	0	0	0	0	0	0
CYP2D6 substrate	0	0	0	0	0	0	0	0
HLM	0	0	0	0	0	0	0	0
RLM	0	0	0	0	0	0	0	0
UGT substrate	1	1	1	1	0	1	1	1
**Excretion**								
Plasma clearance	0	0	0	0	0	0	0	0
Renal Clearance	0	1	1	1	1	1	1	1
Half-life (T1/2)	−0.9	−0.14	−0.34	−0.56	−0.79	−0.35	−0.52	−0.75
Mean retention time	−0.77	−0.11	−0.11	−0.41	−0.53	−0.08	−0.3	−0.55
**Toxicity**								
Neurotoxicity	−2.77	−2.66	−2.67	−2.69	−2.96	−2.59	−2.67	−2.61
hERG 1uM	0	0	0	0	0	0	0	0
Respiratory toxicity	1	1	1	1	1	1	1	1
Nephrotoxicity	0	0	0	0	0	0	0	0
Eye irritation	0	0	0	0	0	0	0	0
Skin irritation	0	0	0	0	0	0	0	0
Acute dermal toxicity	1	1	1	1	1	1	1	1
Mouse carcinogenicity	0	0	0	0	0	0	0	0
Ames mutagenesis	0	0	0	0	0	0	0	0
Reproductive toxicity	1	1	1	1	1	1	1	1
Hemolytic toxicity	1	1	1	1	1	1	1	1
Repeated dose toxicity	0	0	0	0	0	0	0	0
Acute oral toxicity	0	1	1	0	1	1	1	1
Fish toxicity	1	1	1	1	1	1	1	1
Bee toxicity	0	0	0	0	1	1	0	0
Biodegradability	0	0	0	0	0	0	0	0
**Endocrine disruption**								
Androgen receptor	0	0	0	0	0	0	0	0
Estrogen receptor	0	0	0	0	0	0	0	0
Aromatase	0	0	0	0	0	0	0	0
ARE	0	0	0	0	0	0	0	0
HSE	0	0	0	0	0	0	0	0
P53	0	0	0	0	0	0	0	0
PPARϒ	0	0	0	0	0	0	0	0
MMP	0	0	0	0	0	0	0	0
TR	0	0	0	0	0	0	0	0
GR	0	1	0	0	0	0	0	0
**Medicinal chemistry**								
QED	0.16	0.08	0.06	0.11	0.08	0.06	0.1	0.08
Pfizer rule	Accept	Accept	Accept	Accept	Accept	Accept	Accept	Accept

The compounds, Gymnemaside A to XIV, demonstrate a range of pharmacological activities across various targets (Table 7). Most compounds show potential as GPCR ligands, with Gymnemic acid X exhibiting the highest positive score at 0.18. Gymnemagenin and Gymnemic acid VII to XIV indicate potential as ion channel modulators, while Gymnemic acid III and IV show promise as kinase and protease inhibitors, respectively. Gymnemic acid VI and VIII to XIV have positive scores as nuclear receptor ligands, and Gymnemagenin and Gymnemic acid X as enzyme inhibitors. These pharmacological activities suggest therapeutic potential, particularly in metabolic disorders, neurological conditions, and inflammation.

**Table 7:** Bioactivity score of Triterpenoid saponins as predicted by Molinspiration Tool.

Compound name	GPCR ligand	Ion channel modulator	Kinase inhibitor	Nuclear receptor ligand	Protease inhibitor	Enzyme inhibitor
Gymnemaside A	−1.62	−2.81	−2.77	−2.03	−1.14	−1.63
Gymnemaside B	−1.64	−2.83	−2.78	−2.03	−1.15	−1.67
Gymnemagenin	0.18	−0.09	−0.26	0.67	0.17	0.54
Gymnemic acid I	−1.62	−2.79	−2.77	−1.98	−1.13	−1.62
Gymnemic acid II	−1.59	−2.78	−2.76	−2.02	−1.03	−1.66
Gymnemic acid III	−1.04	−2.18	−2.10	−1.31	−0.61	−1.06
Gymnemic acid IV	−1.08	−2.19	−2.11	−1.26	−0.72	−1.02
Gymnemic acid V	−2.26	−3.26	−3.36	−2.63	−1.68	−2.30
Gymnemic acid VI	−3.14	−3.79	−3.96	−3.26	−2.72	−2.98
Gymnemic acid VII	−0.14	−1.02	−0.91	−0.12	−0.04	0.01
Gymnemic acid VIII	−3.12	−3.79	−3.97	−3.29	−2.62	−2.99
Gymnemic acid IX	−3.15	−3.80	−3.98	−3.26	−2.71	−2.96
Gymnemic acid X	−0.59	−1.59	−1.48	−0.71	−0.26	−0.54
Gymnemic acid XI	−2.26	−3.25	−3.35	−2.58	−1.67	−2.29
Gymnemic acid XII	−3.49	−4.01	−4.24	−3.54	−3.16	−3.27
Gymnemic acid XIII	−1.03	−2.21	−2.08	−1.33	−0.59	−1.08
Gymnemic acid XIV	−1.07	−2.22	−2.10	−1.28	−0.70	−1.04

Medicinally, all compounds have QED values above 0.06, indicating good druglikeness, and they all adhere to the Pfizer rule, reflecting favourable medicinal chemistry properties. To further optimize the bioavailability and druglikeness of these compounds, several strategies can be employed. Enhancing solubility through prodrug design or salt formation could improve their oral bioavailability. Utilizing nanoformulations or lipid-based formulations can also enhance solubility and absorption. Modifying the chemical structure to reduce plasma protein binding and P-glycoprotein substrate potential may improve their pharmacokinetic profile. *In vitro*-*in vivo* correlation (IVIVC) studies and in vivo pharmacokinetic evaluations are essential to validate these strategies. For inclusion as drug candidates or herbal ingredients, the compounds can be encapsulated in nanoparticles or microparticles to enhance their stability and bioavailability. The formulation can be further optimized by employing natural permeation enhancers or by using permeation enhancer-loaded microspheres. Additionally, the compounds can be incorporated into solid lipid nanoparticles or nanoemulsions to improve their absorption and bioavailability. In summary, Gymnemic acid I to XIV display promising ADMET profiles with potential therapeutic utility. Advanced formulation techniques and medicinal chemistry strategies can be employed to maximize their efficacy and safety profiles for drug development or inclusion in herbal formulations. Further studies, including in vivo pharmacokinetic and pharmacodynamic evaluations, are essential to determine their therapeutic potential, safety profiles, and optimal formulation strategies for drug development or herbal formulations.

**Fig. 14:**
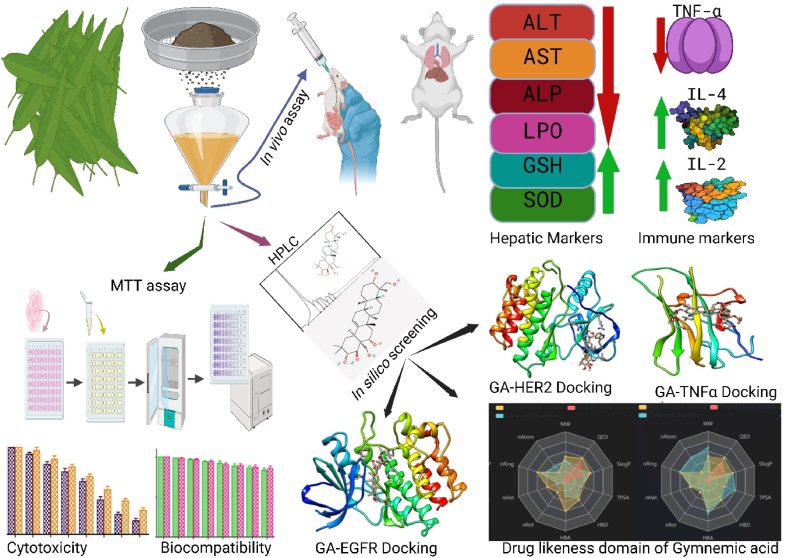
Schematic representation of investigating the therapeutic potential of triterpenoid saponins from *G. sylvestre*: Hepatoprotective, immunomodulatory, and anticancer activities, molecular docking, and pharmacokinetics insights.

Fig. 14 illustrates a schematic representation of the comprehensive investigation into the hepatoprotective, immunomodulatory, and anticancer activities of the triterpenoid saponins found in *G. sylvestre*. Additionally, molecular docking and pharmacokinetic insights are depicted, offering a holistic view of their potential therapeutic applications.

## 4 Conclusions

In conclusion, this comprehensive study robustly elucidates the multifaceted therapeutic potential of *G. sylvestre* triterpenoid saponin extract (GST). Employing a Prednisolone-induced immunosuppressed rat model, GST demonstrated significant hepatoprotective, and immunomodulatory activities. The study revealed that GST effectively modulates haematopoiesis, improving RBC, platelet, and WBC counts, thereby highlighting its potential in maintaining hematopoietic homeostasis. The hallmark of immunosuppression, organ atrophy, in spleen, thymus, liver, and kidneys was reversed with GST treatment, emphasizing its hepatotrophic and organotropic capabilities. Elevated hepatic biomarkers (ALT, AST, ALP, LPO) indicative of hepatocellular injury and oxidative stress were notably reduced with GST, underscoring its hepatoprotective and antioxidative effects. Furthermore, GST restored depleted antioxidants (GSH and SOD), emphasizing its potent antioxidative and free radical scavenging capabilities. Molecular insights into immune dysregulation revealed a downregulation of IL-2 and IL-4 mRNA expression in the spleen of immunosuppressed rats. Notably, GST treatment led to a significant upregulation of IL-2 and IL-4 mRNA expression, accentuating its immunomodulatory potential in orchestrating immune homeostasis. Additionally, the elevated levels of TNF-α associated with immune dysregulation were effectively decreased with GST, further highlighting its role in modulating inflammatory responses and restoring immune balance. Molecular docking studies unveiled robust binding interactions between *G. sylvestre* compounds and TNF-α. Gymnemic acid VI, along with Gymnemic acids IX, I, XIII, and XIV, exhibited the most potent binding affinities, underscoring their potential as promising TNF-α inhibitors. The identification of key residues involved in these interactions offers crucial insights into the varied inhibitory capabilities of *G. sylvestre* compounds against TNF-α. These pivotal findings set the stage for the development of innovative biological inhibitors aimed at treating inflammatory and autoimmune diseases.

In terms of anticancer activity, GST demonstrated significant cytotoxicity against breast cancer cell lines, particularly MCF-7. This cytotoxicity was corroborated by molecular docking studies, which identified strong binding of GST compounds to breast cancer proteins HER1 and HER2, with Gymnemic acid XIII and Gymnemic acid V exhibiting the highest binding energies. Moreover, the biocompatibility of GST was rigorously assessed against normal CHO and HUVEC cell lines to ensure safety, which is paramount for therapeutic applications. Encouragingly, GST did not exhibit any cytotoxic effects on these normal cell lines at lower concentrations. Molecular docking studies identified 15 lead compounds from *G. sylvestre* that selectively inhibit HER1 or EGFR, suggesting their potential utility in designing selective EGFR inhibitors. These compounds function by inhibiting HER2, thereby impeding the growth and proliferation of HER2-positive breast cancer cells. These findings pave the way for the development of novel inhibitors targeting these proteins, contributing significantly to the advancement of breast cancer treatment strategies.

Furthermore, pharmacokinetics, bioavailability, drug-likeness, and ADMET profiles predictions suggest potential of triterpenoid saponins of *G. sylvestre* as a pharmacologically favourable agent with no serious adverse effects. Advanced formulation techniques and medicinal chemistry strategies can further optimize their efficacy and safety for drug development or inclusion in herbal formulations. Strategies to enhance their bioavailability include prodrug design, nanoformulations, or lipid-based formulations, while encapsulation in nanoparticles or microparticles can improve stability and bioavailability.

## CRediT authorship contribution statement

**Vasudeva Reddy Netala:** Writing – original draft, Methodology, Investigation, Conceptualization. **Tianyu Hou:** Validation, Software. **Rajakumari Devarapogu:** Methodology, Investigation. **Murali Satyanarayana Bethu:** Methodology, Formal analysis. **Zhijun Zhang:** Writing – review & editing, Validation. **Tartte Vijaya:** Supervision.

## Ethical approval

Applicable and included approval number in the manuscript.

## Availability of data and materials

All data generated or analyzed during this study are included within this article.

## Funding

Not Applicable.

## Declaration of competing interest

The authors declare that they have no known competing financial interests or personal relationships that could have appeared to influence the work reported in this paper.
