# Supplementary material for: Exploring the therapeutic potential of triterpenoid saponins from Gymnema sylvestre: Mechanistic insights into hepatoprotection, immunomodulation, anticancer activities, molecular docking, and pharmacokinetics
Source: Heliyon. 2024 Nov 30;10(23):e40850. doi: 10.1016/j.heliyon.2024.e40850 (PMC11666954; doi:10.1016/j.heliyon.2024.e40850)
Supplement: Multimedia component 1 [file mmc1.doc]

**Fig. 1S** represents molecular docking between Triterpenoids of *G. sylvestre* and TNF-α

| 3D overview of Gymnemaside A-TNFα complex  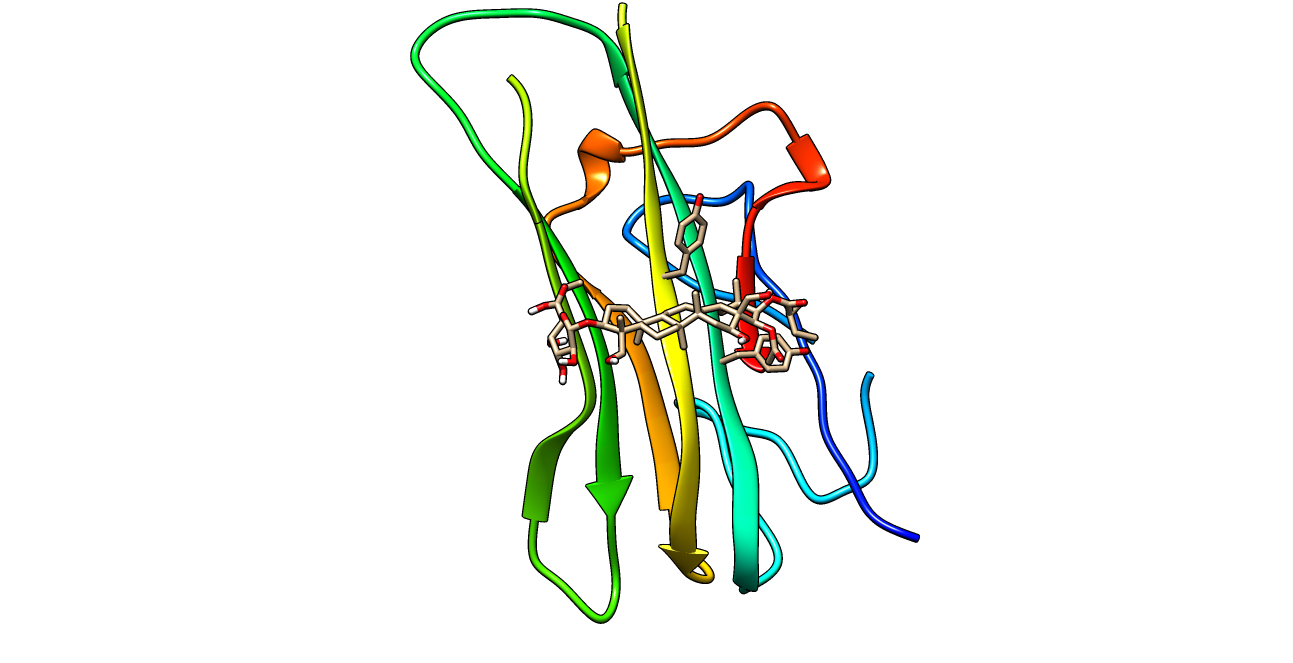 | 2D model of Gymnemaside A-TNFα complex showing active site residues and binding interactions  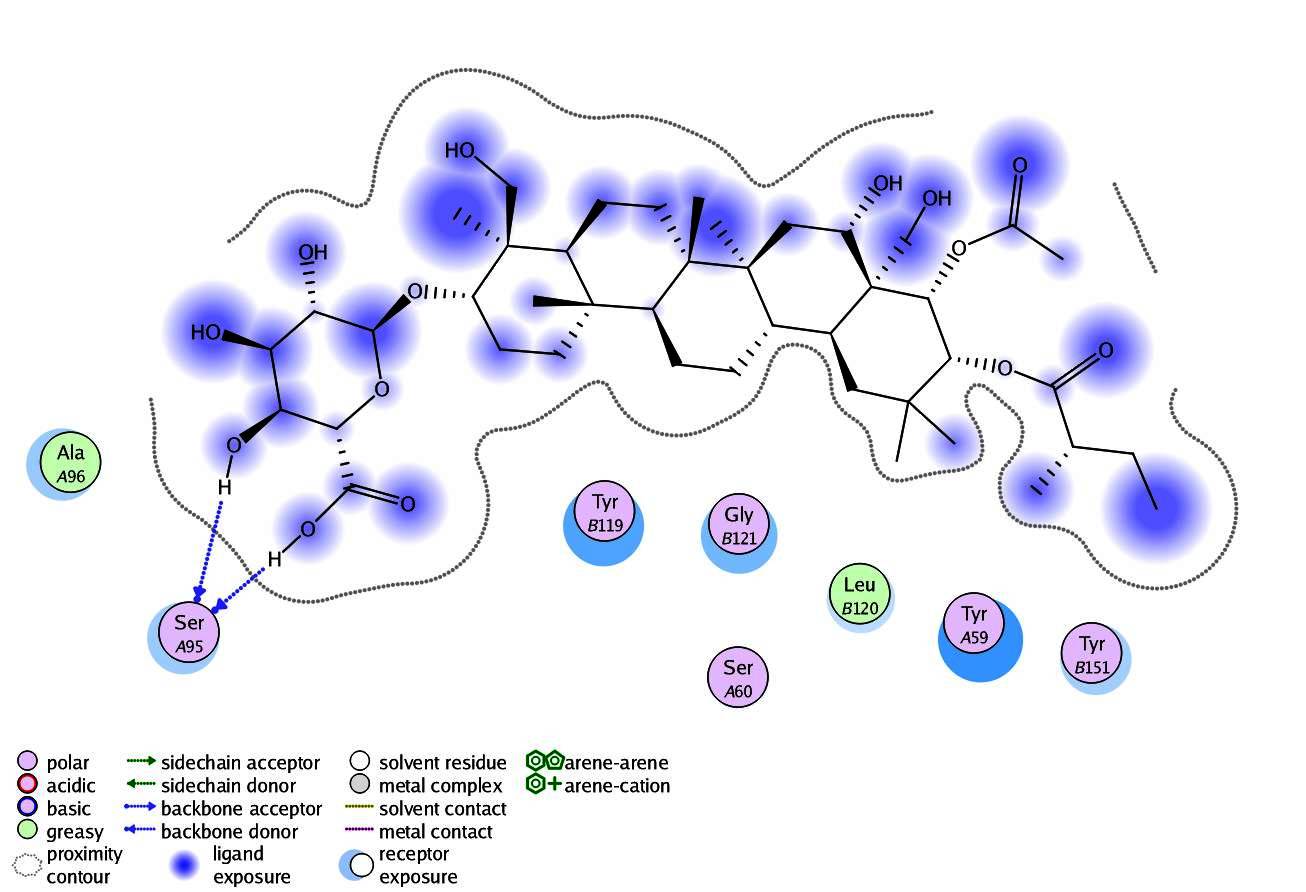 |
| --- | --- |
| 3D overview of Gymnemaside B-TNFα complex  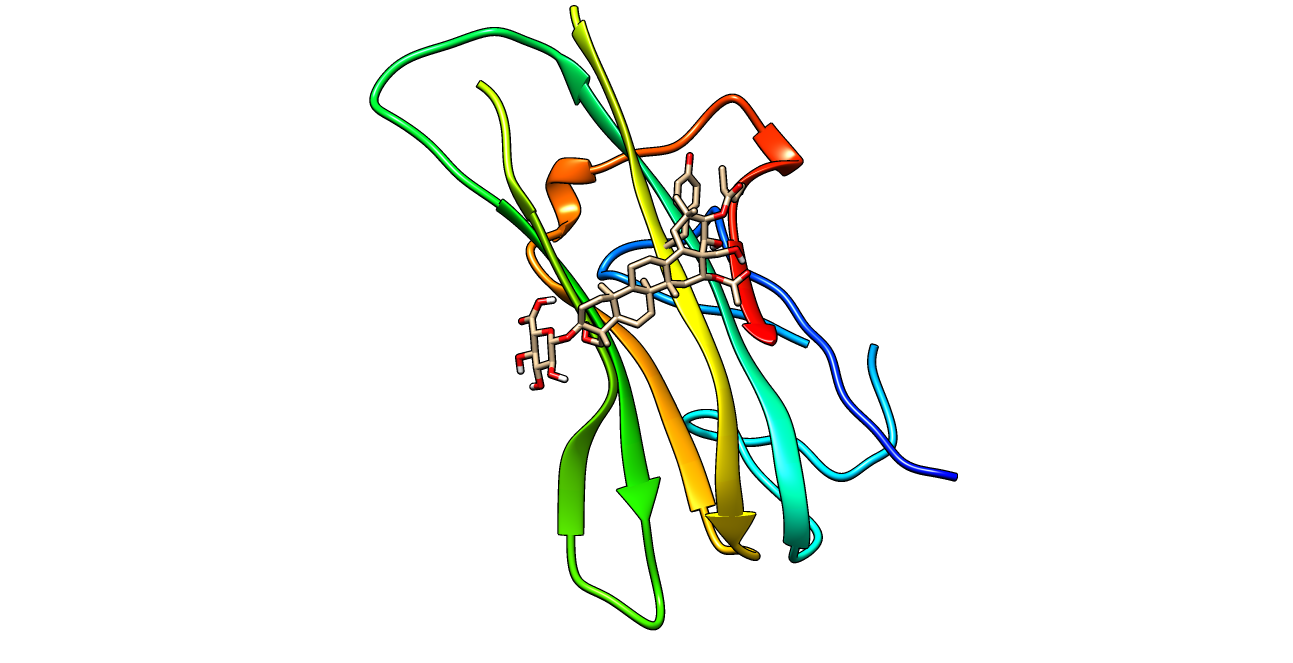 | 2D model of Gymnemaside B-TNFα complex showing active site residues and binding interactions  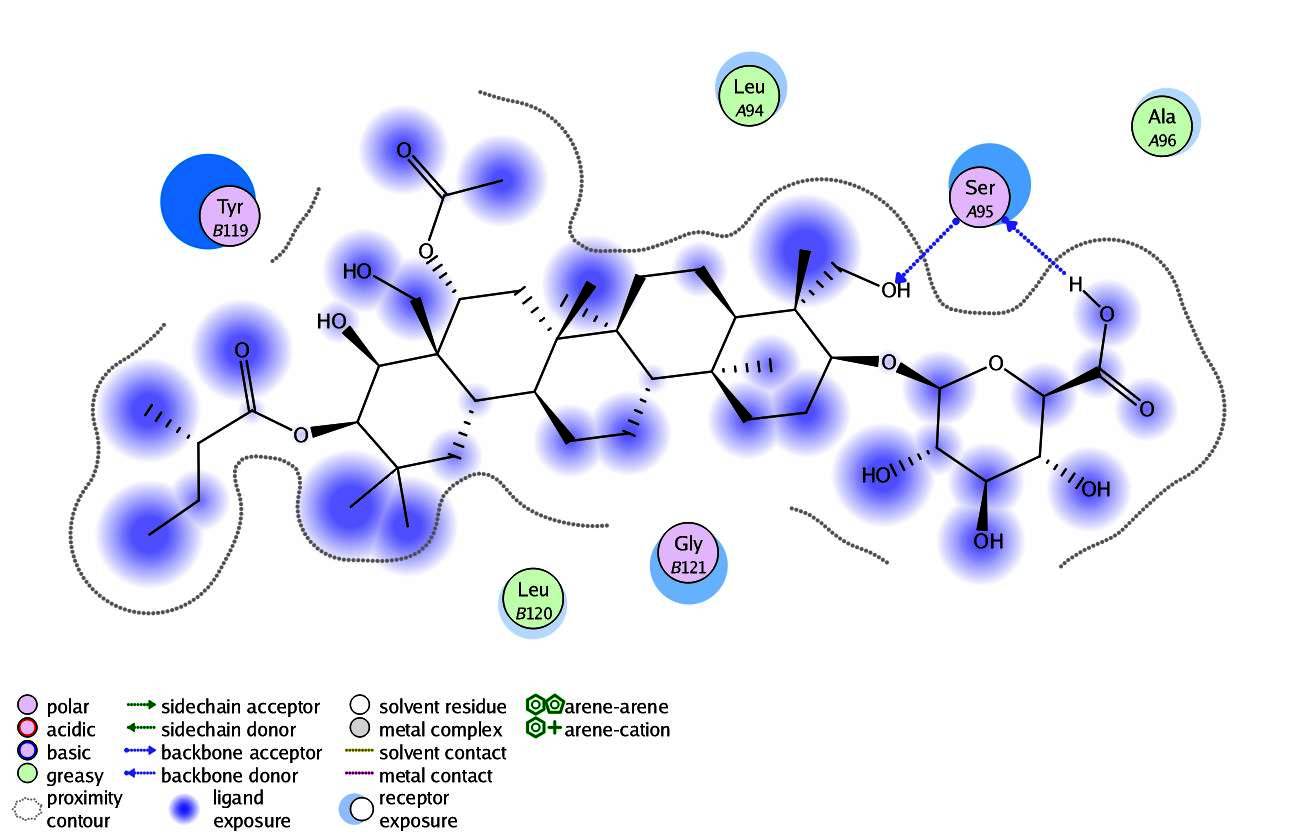 |
| 3D overview of Gymnemagenin-TNFα complex  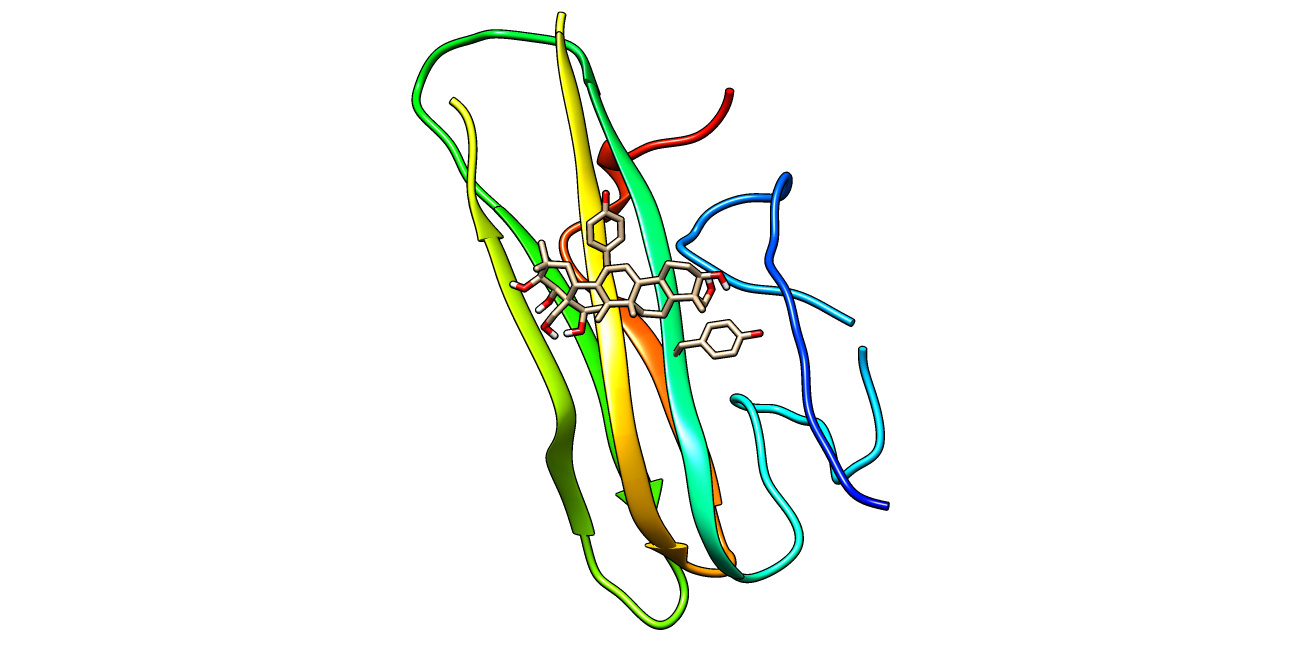  3D overview of Gymnemic acid I-TNFα complex  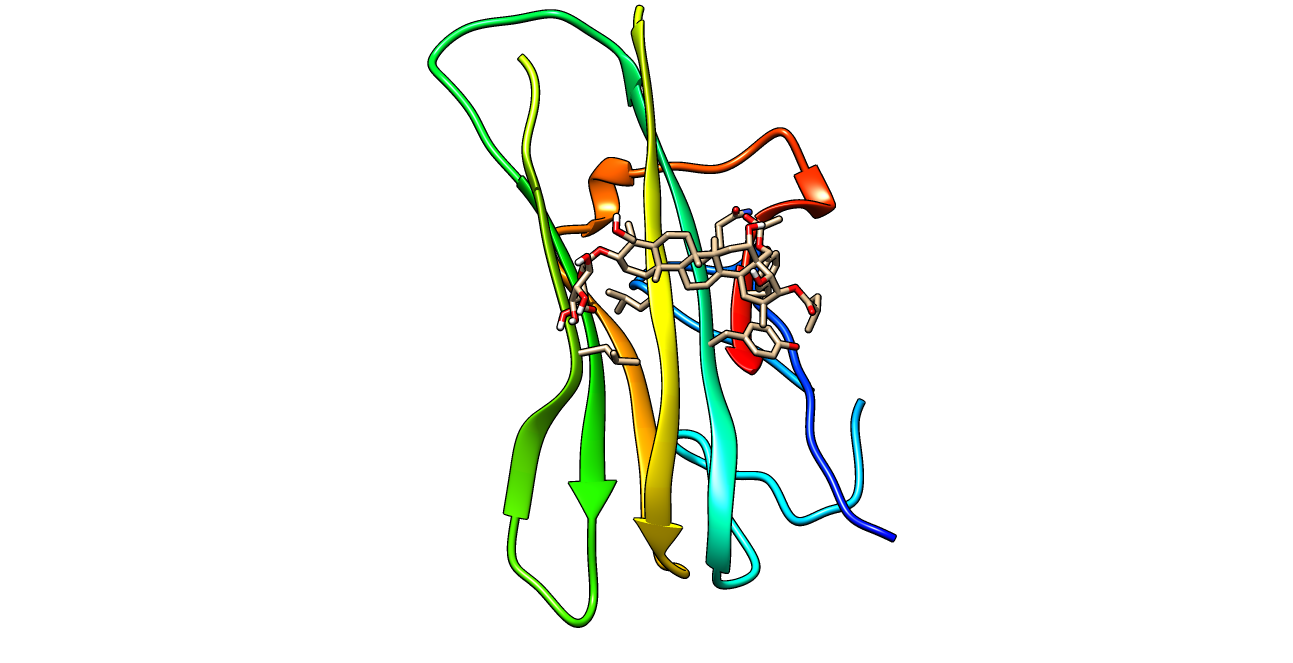 | 2D model of Gymnemagenin-TNFα complex showing  active site residues and binding interactions  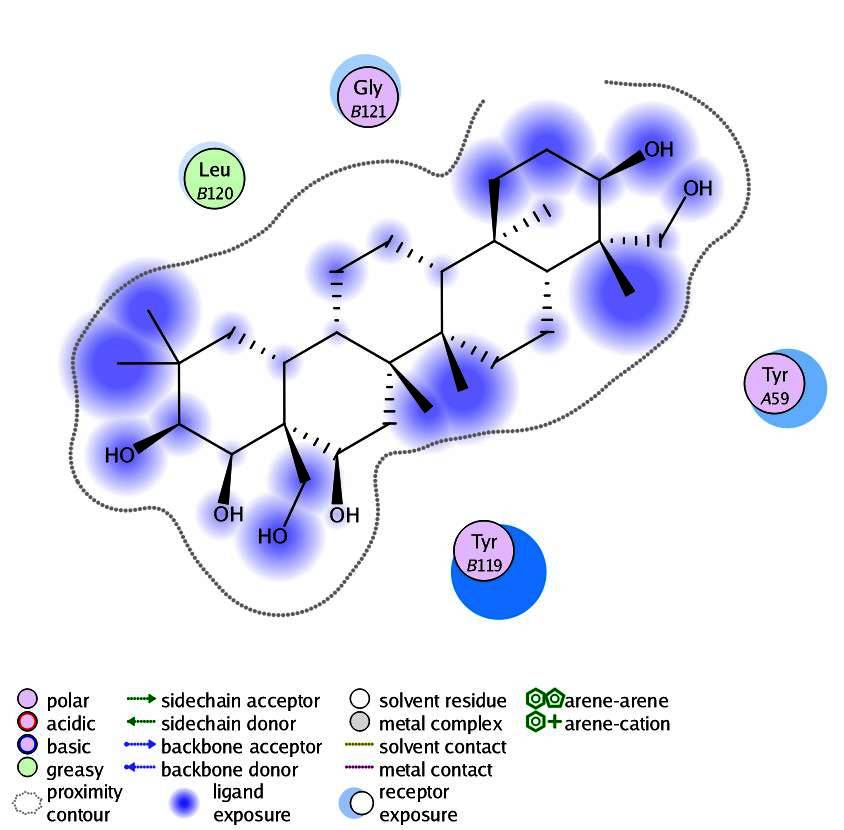  2D model of Gymnemic acid I-TNFα complex showing  active site residues and binding interactions  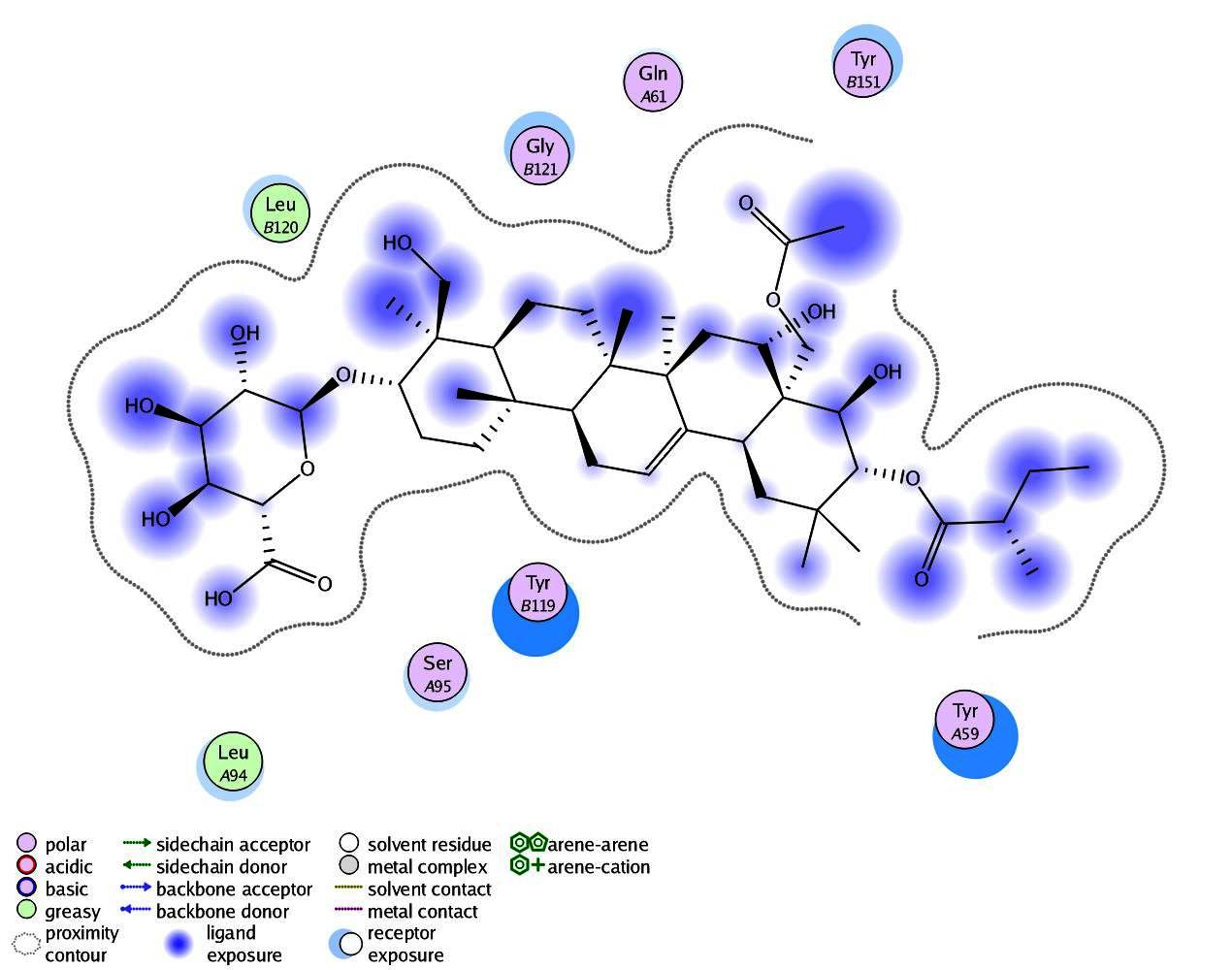 |

| 3D overview of Gymnemic acid II-TNFα complex  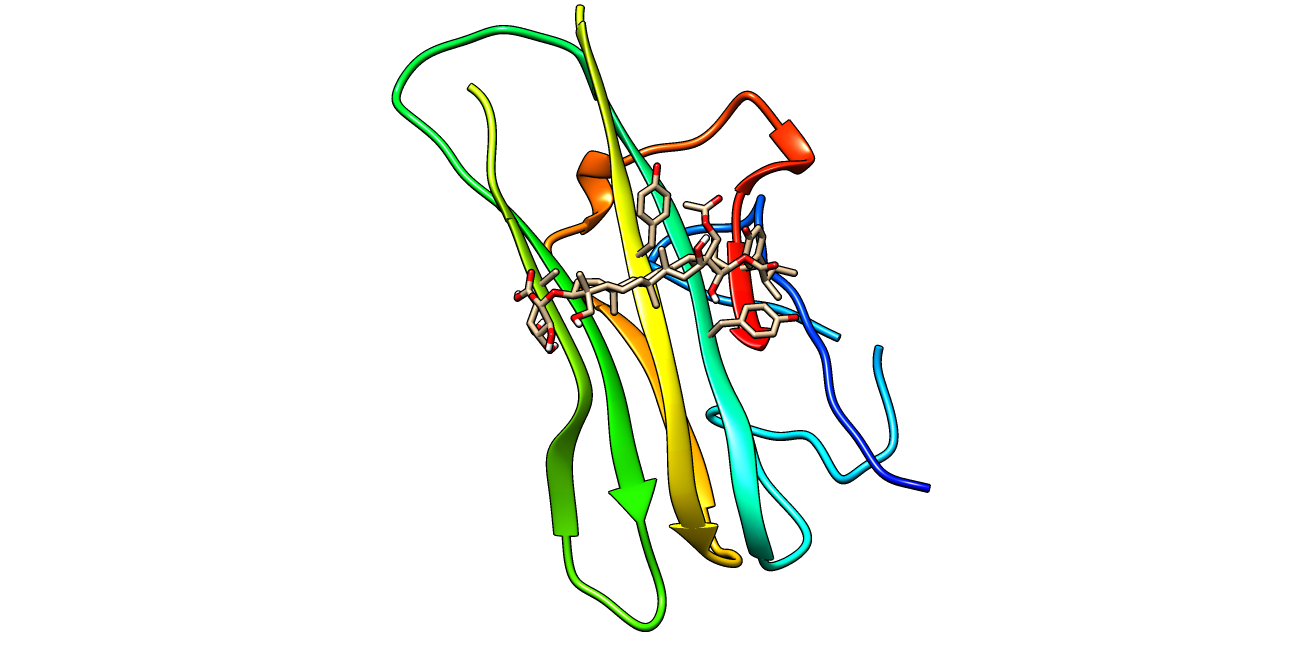 | | 2D model of Gymnemic acid II-TNFα  complex showing active site residues and binding interactions 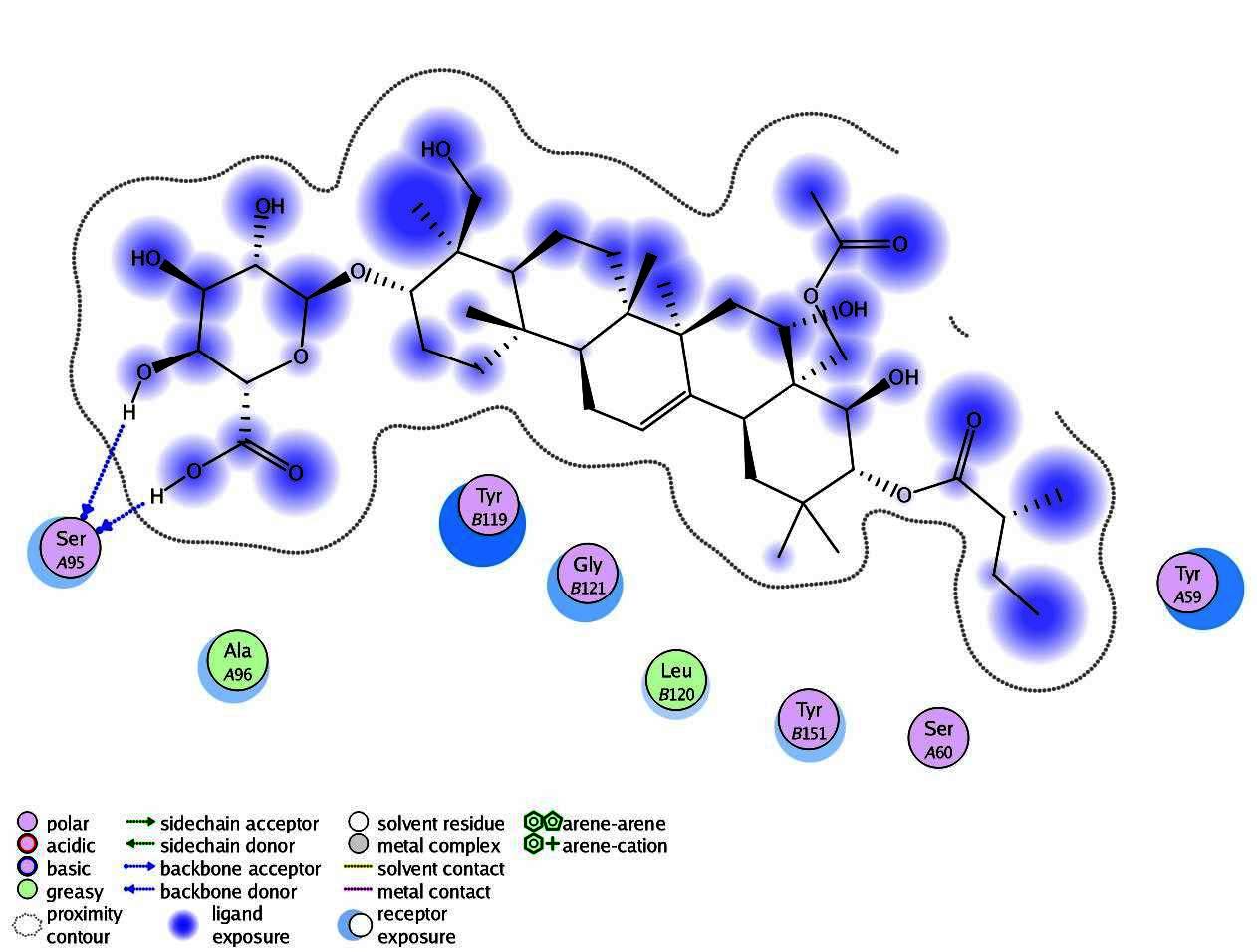 |
| --- | --- | --- |
| 3D overview of Gymnemic acid III-TNFα complex  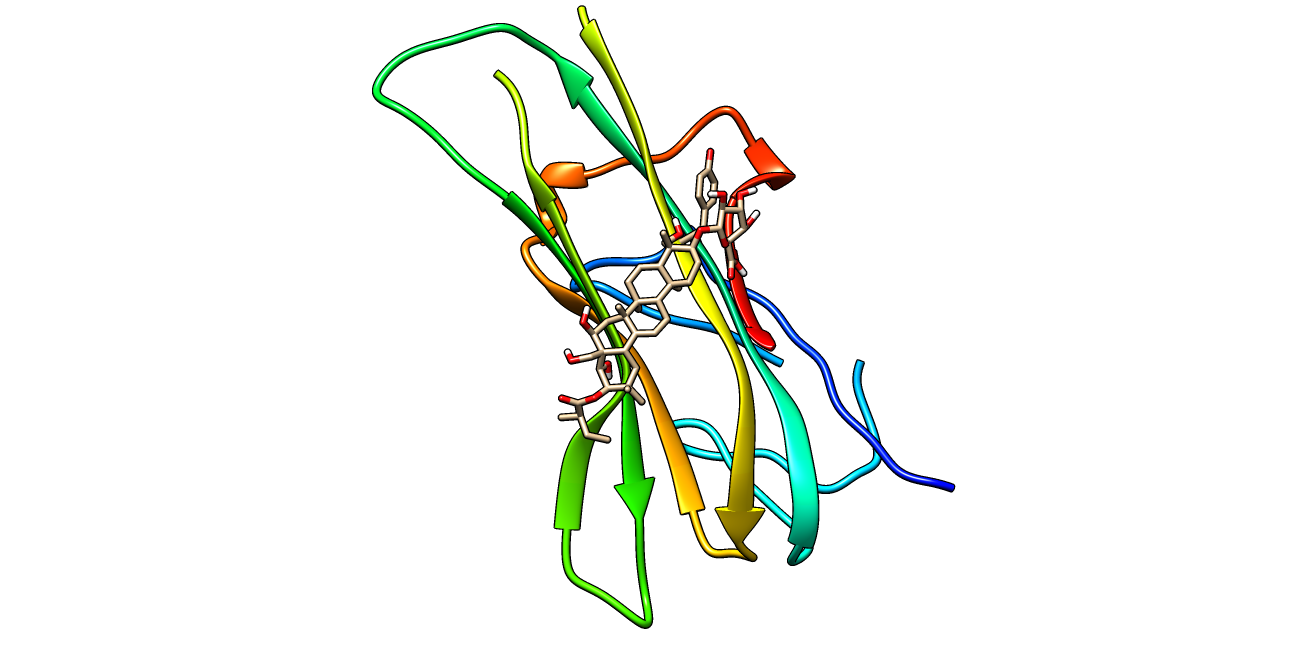 | | 2D model of Gymnemic acid III-TNFα complex showing active site residues and binding interactions  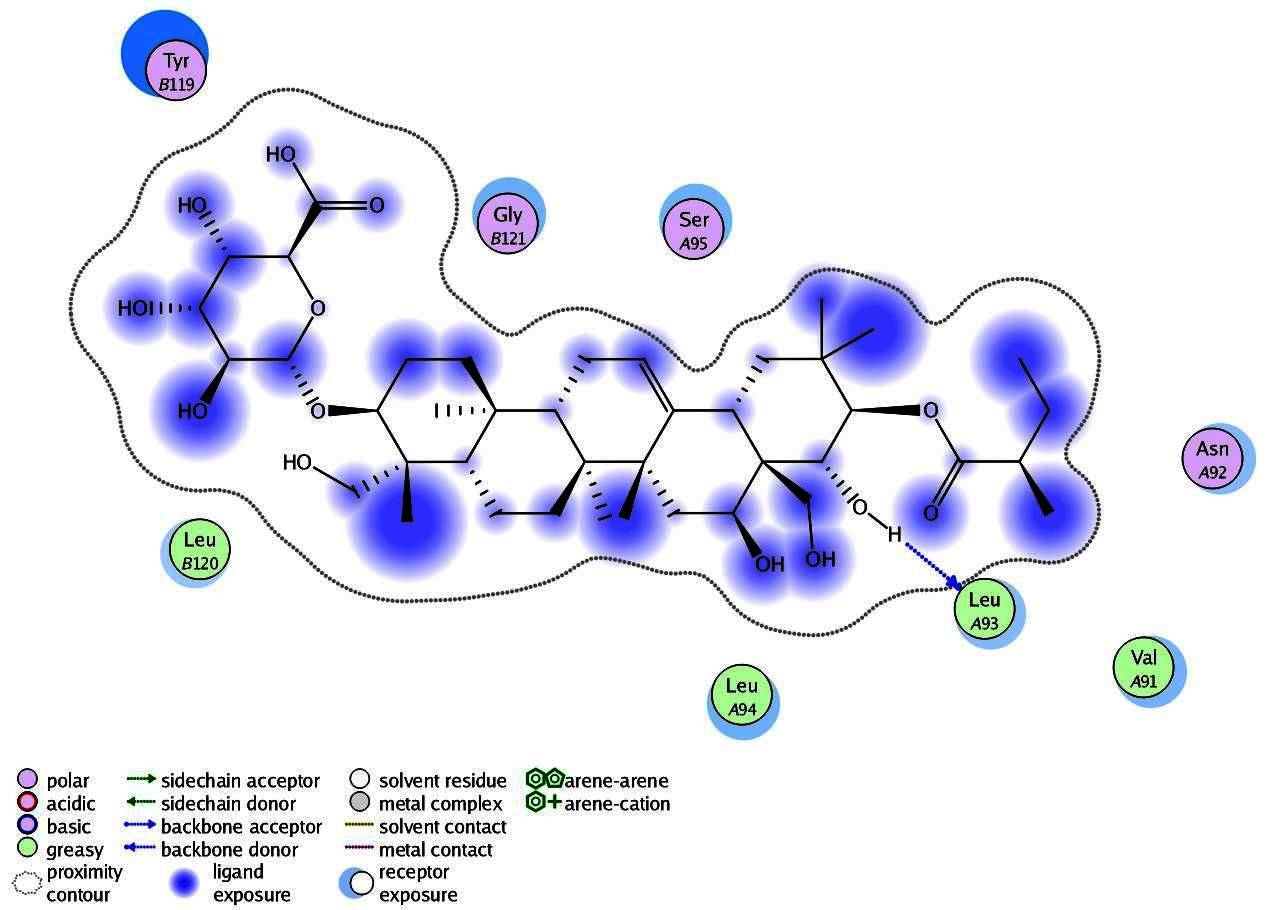 |
|  | |  |
| 3D overview of Gymnemic acid IV-TNFα complex  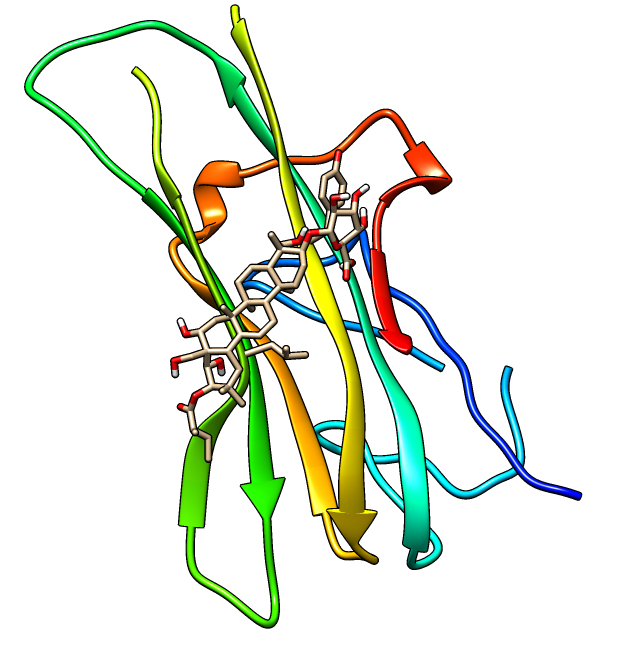 | 2D model of Gymnemic acid IV-TNFα complex showing binding interactions  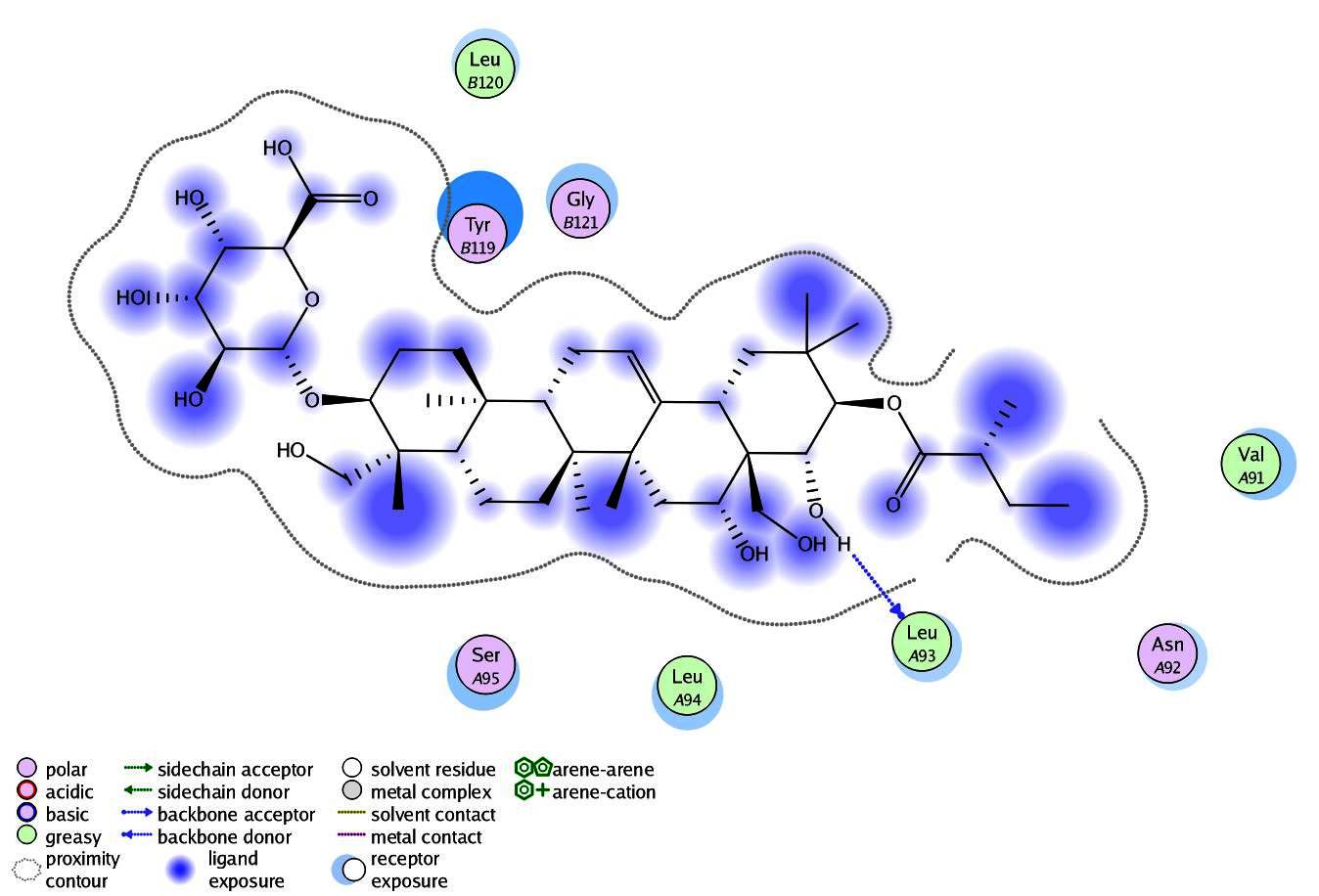 | |
| 3D overview of Gymnemic acid V-TNFα complex  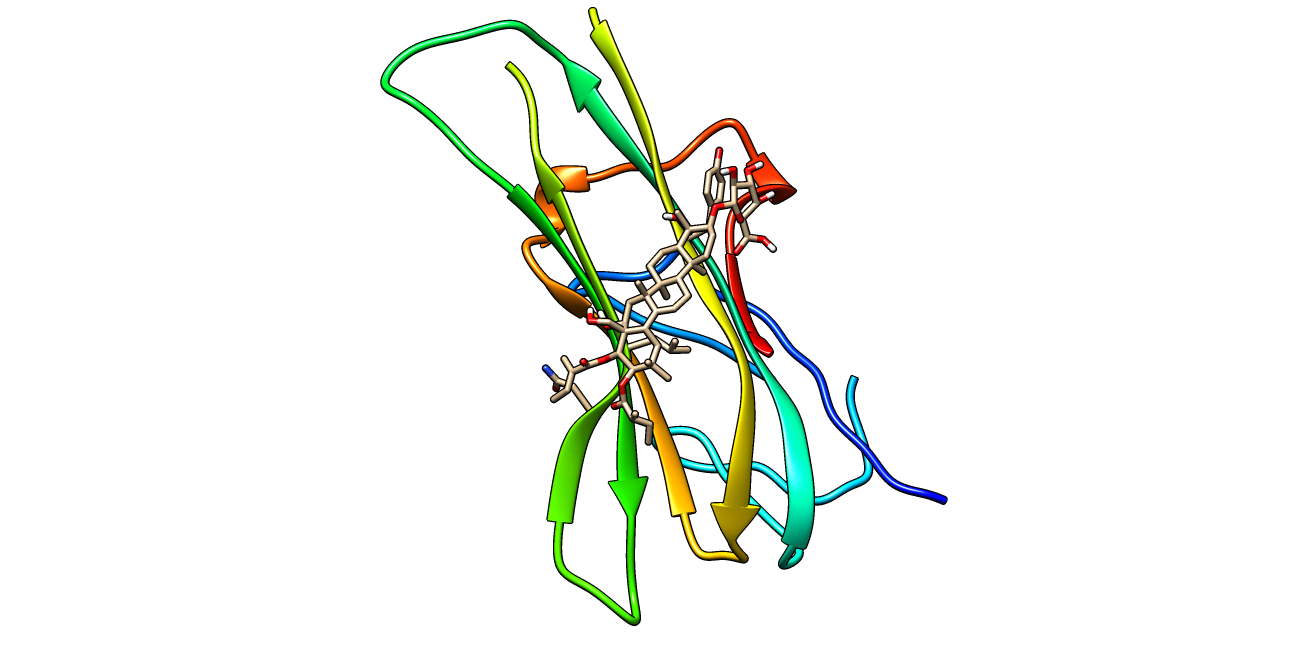 | 2D model of Gymnemic acid V-TNFα complex showing binding interactions  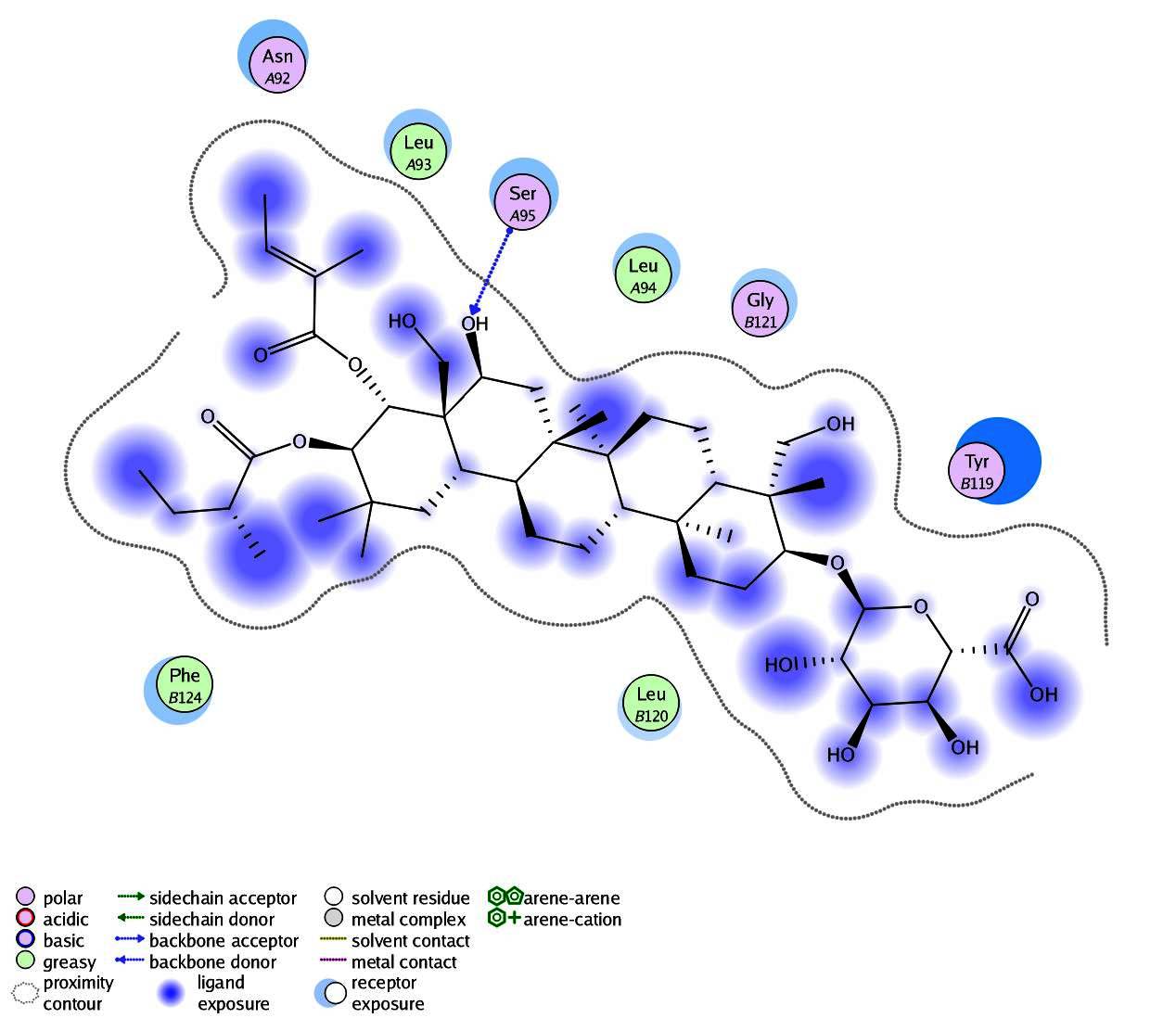 | |
| 3D overview of Gymnemic acid VI-TNFα complex  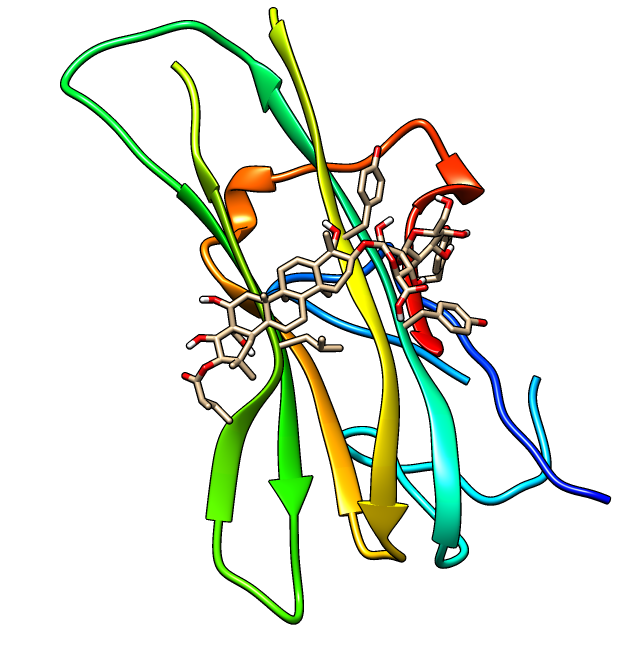 | 2D model of Gymnemic acid VI-TNFα complex showing binding interactions  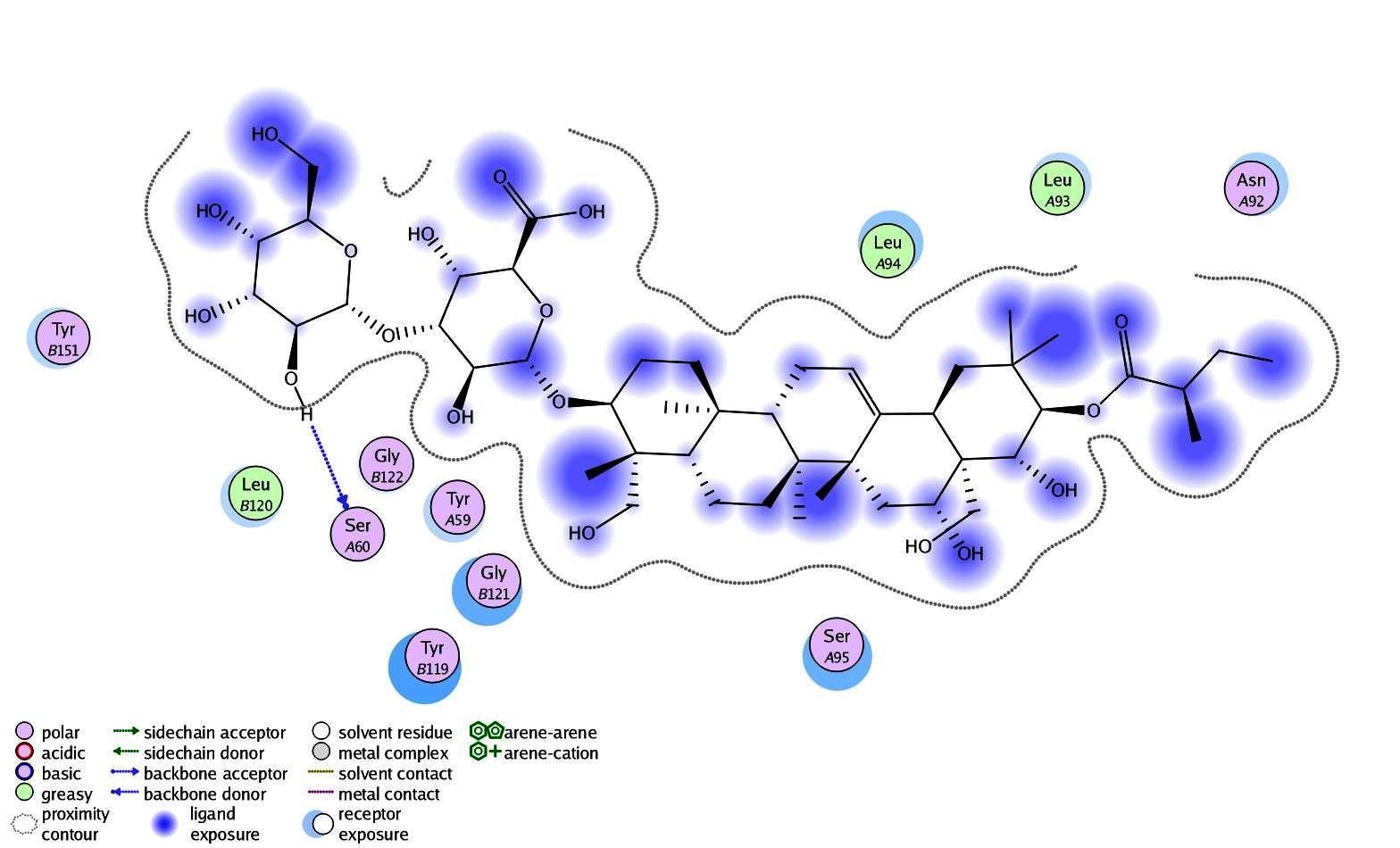 | |
| 3D overview of Gymnemic acid VII-TNFα complex  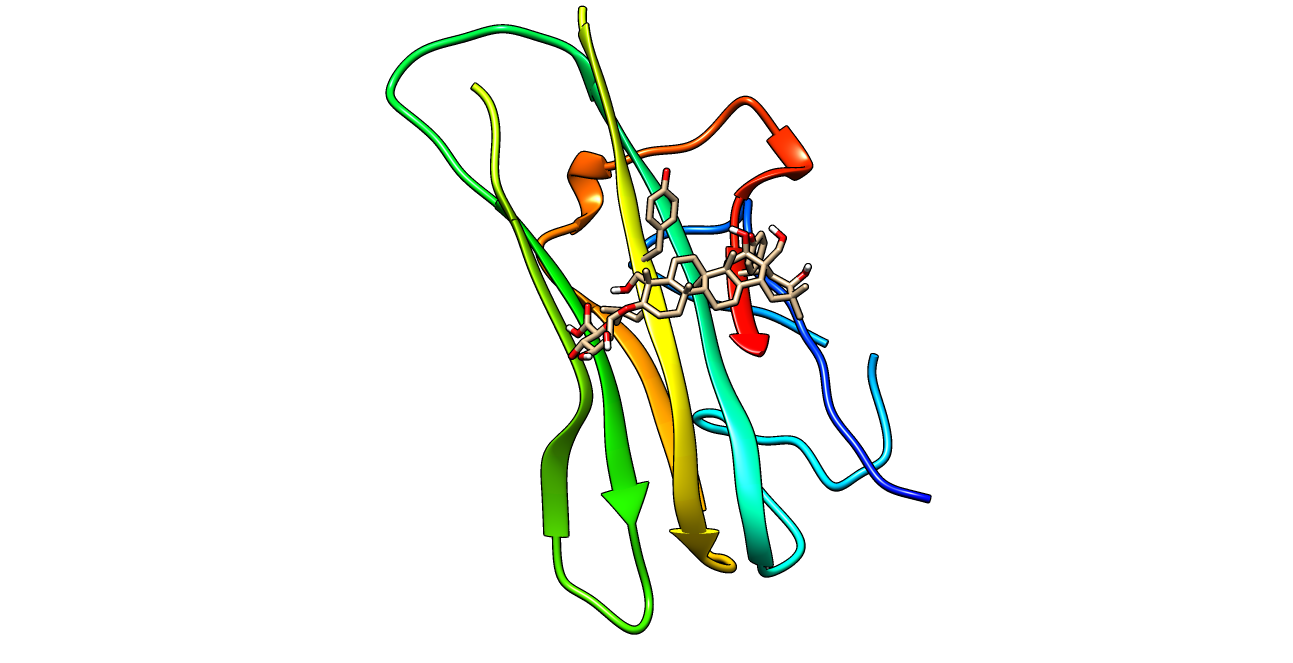 | 2D model of Gymnemic acid VII-TNFα complex showing binding interactions  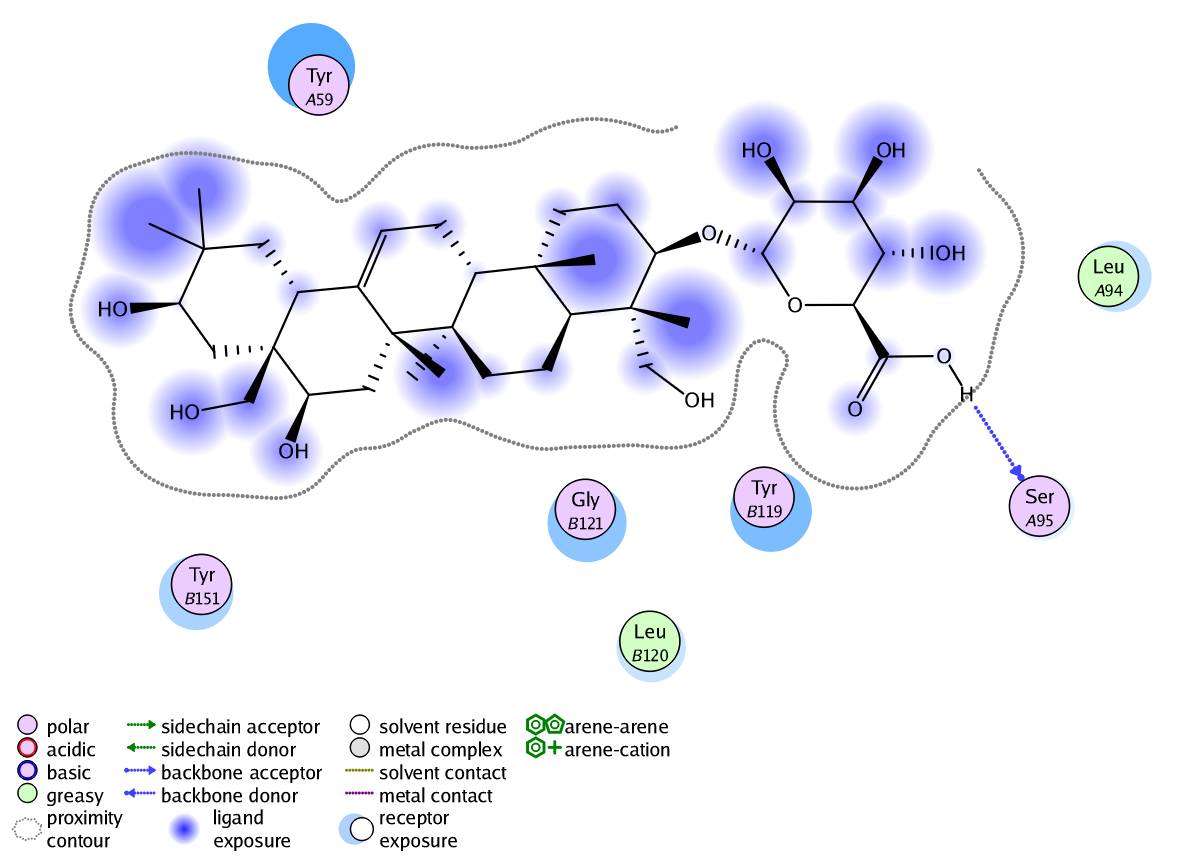 | |

| 3D overview of Gymnemic acid VIII-TNFα complex  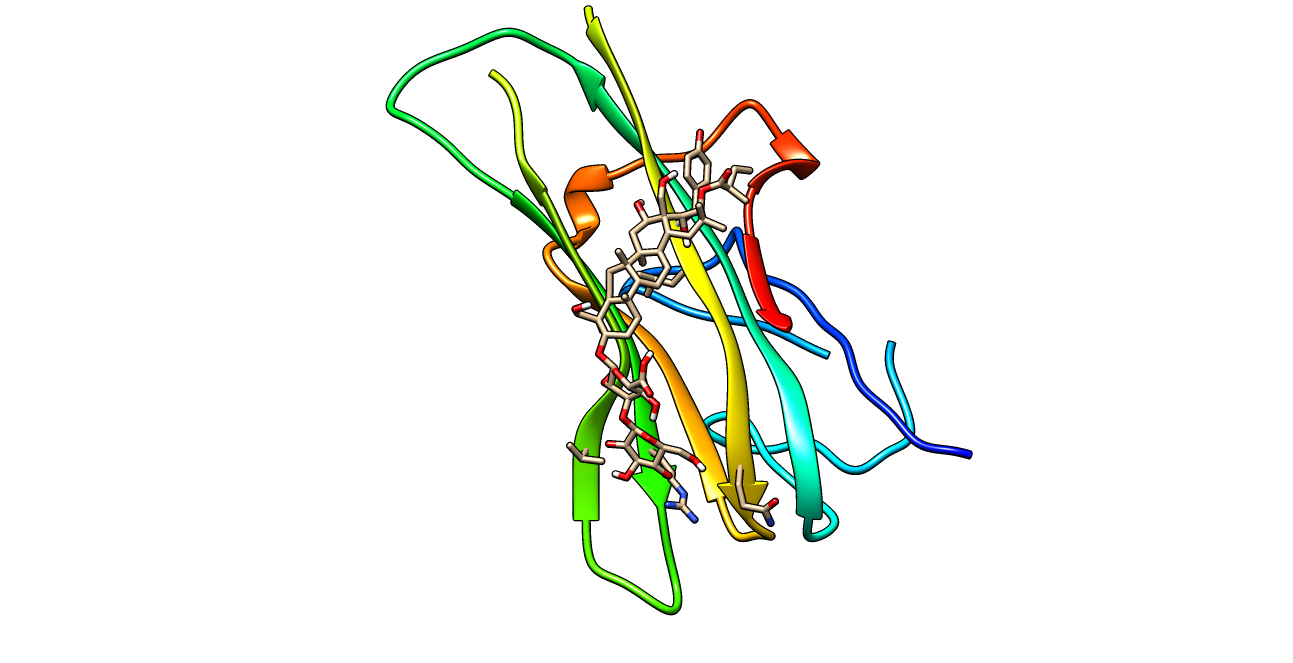 | | | 2D model of Gymnemic acid VIII-TNFα complex showing binding interactions  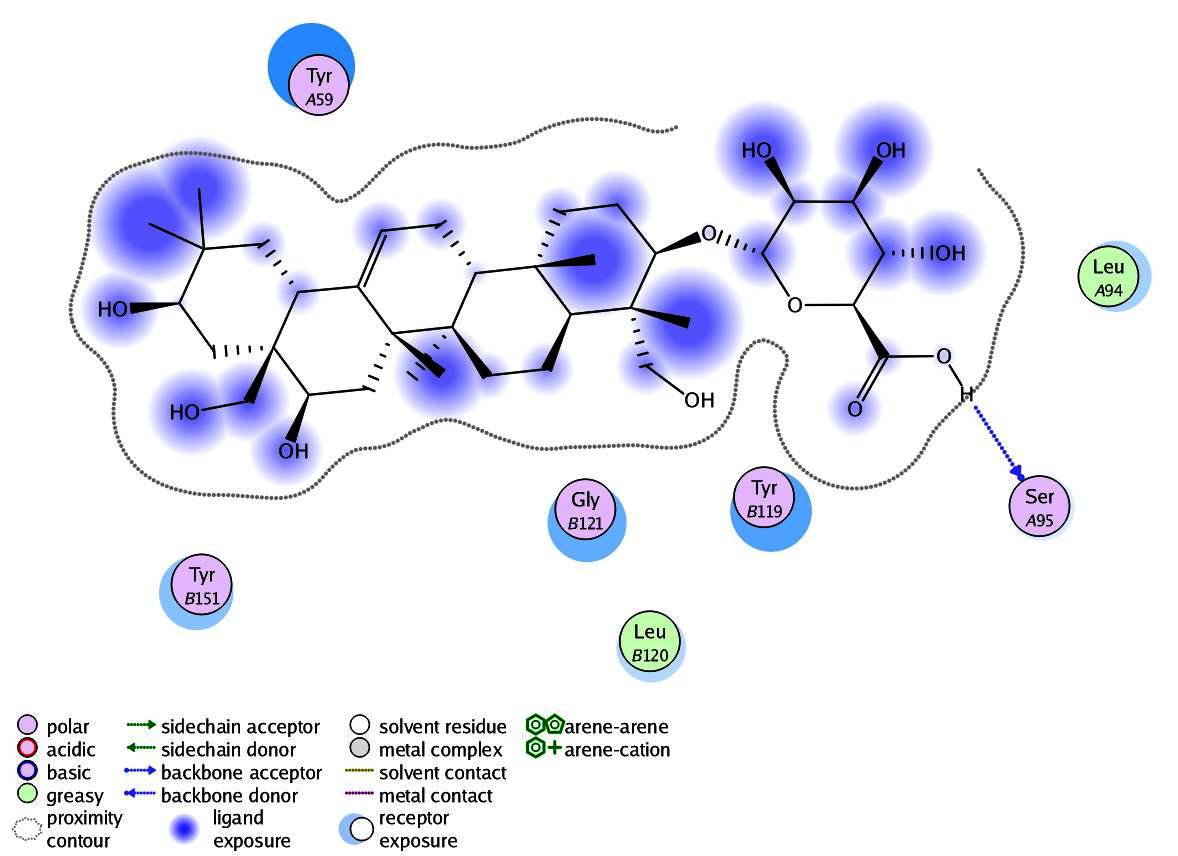 | |
| --- | --- | --- | --- | --- |
| 3D overview of Gymnemic acid IX-TNFα complex  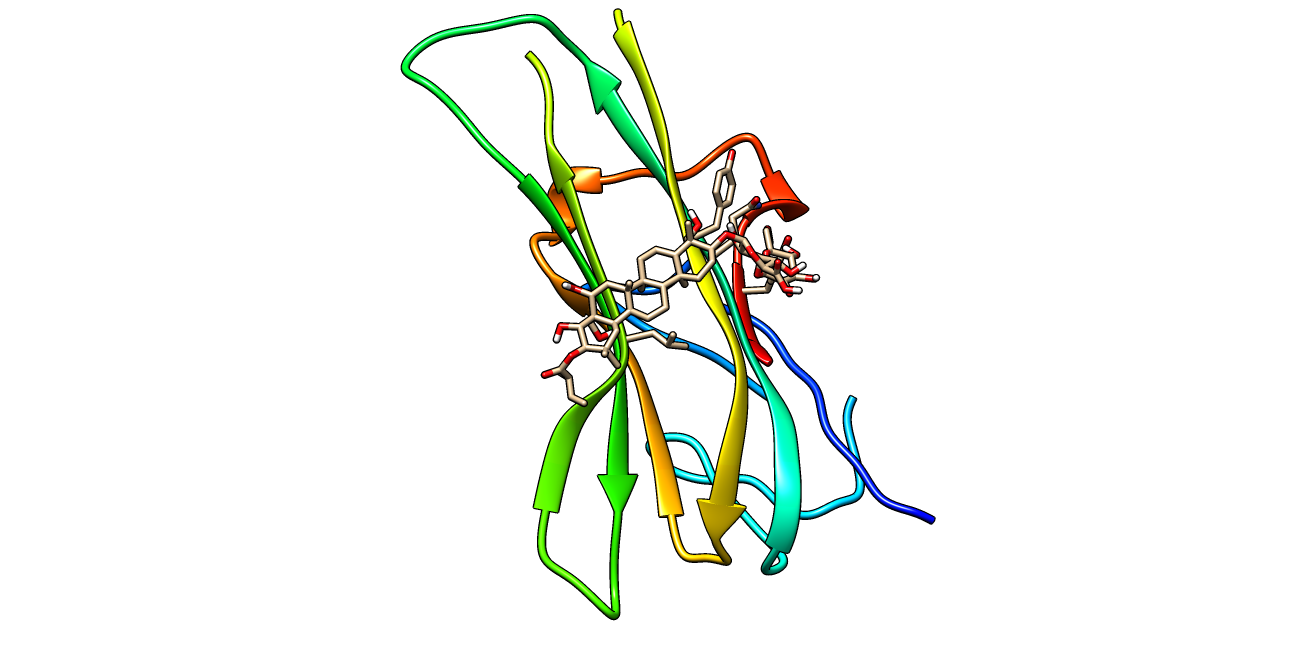 | | | 2D model of Gymnemic acid IX-TNFα complex showing binding interactions  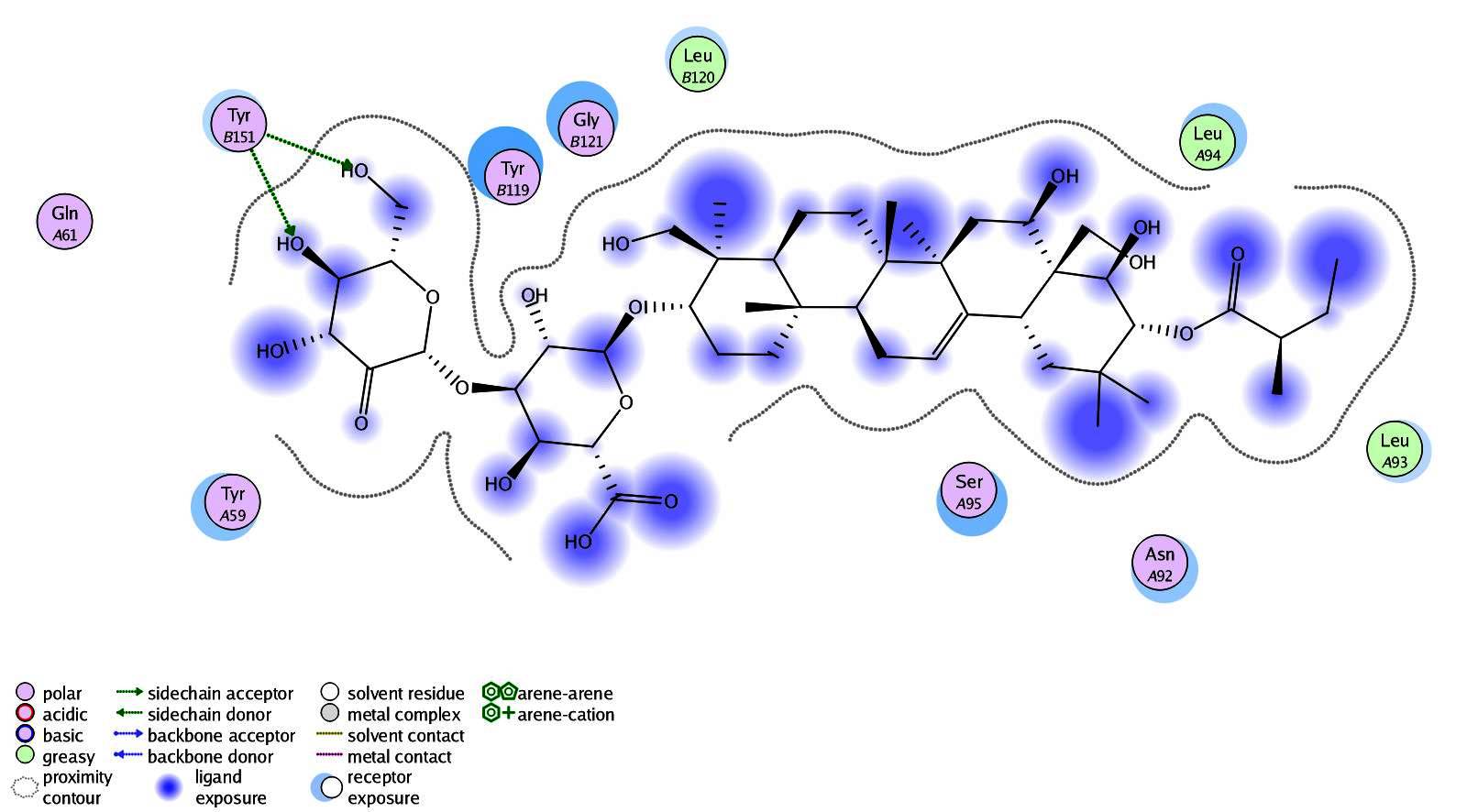 | |
| 3D overview of Gymnemic acid X-TNFα complex  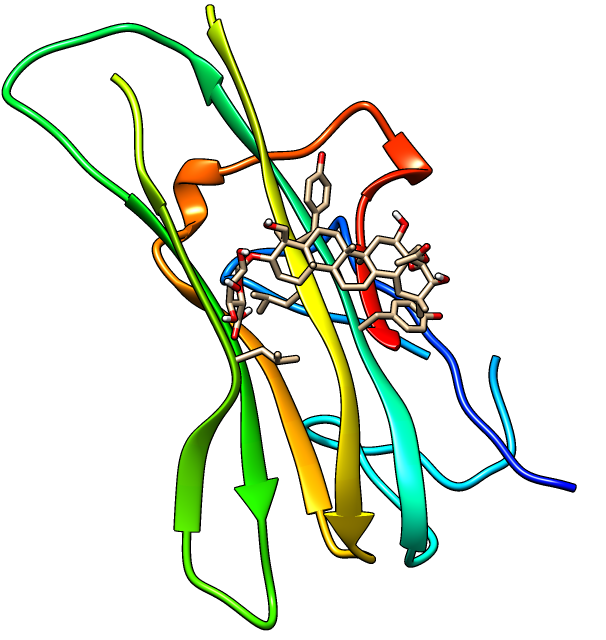 | | 2D model of Gymnemic acid X-TNFα complex showing binding interactions  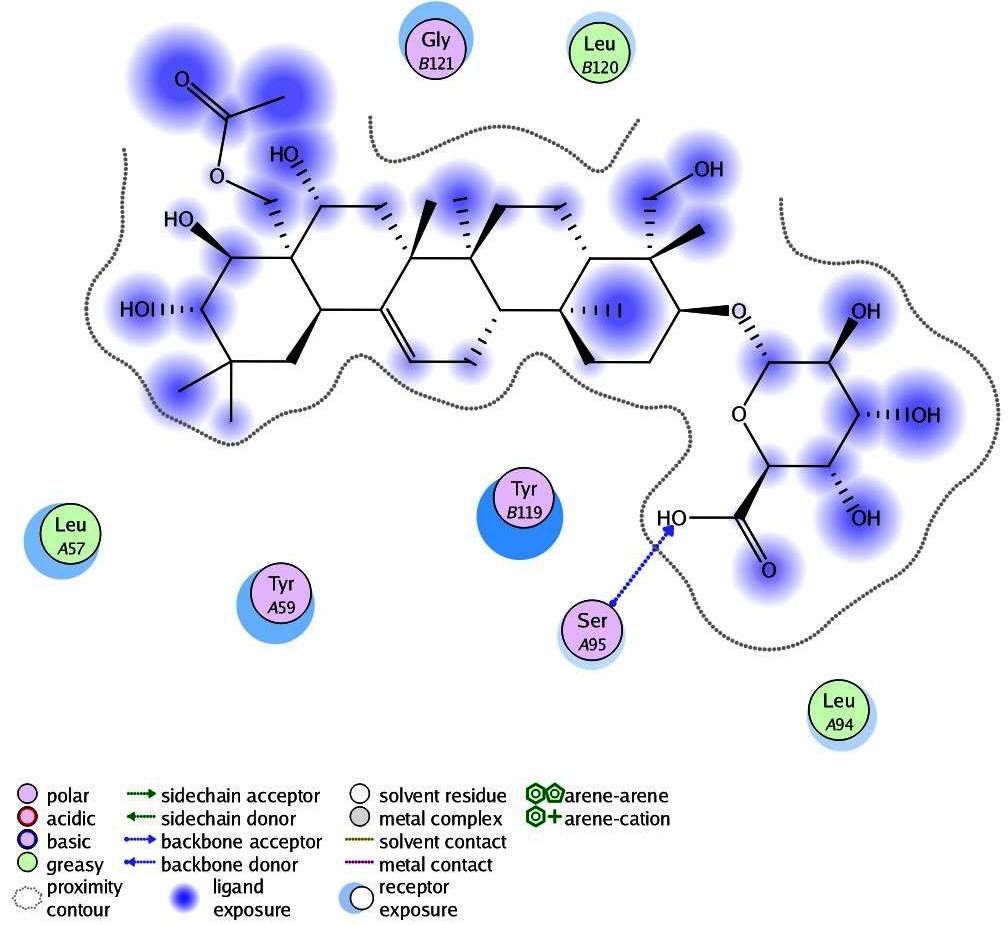 | | |
| 3D overview of Gymnemic acid XI-TNFα complex  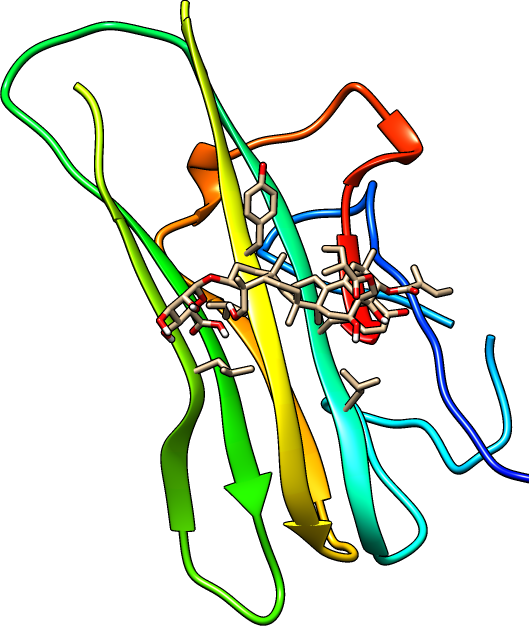 | | 2D model of Gymnemic acid XI-TNFα complex showing binding interactions  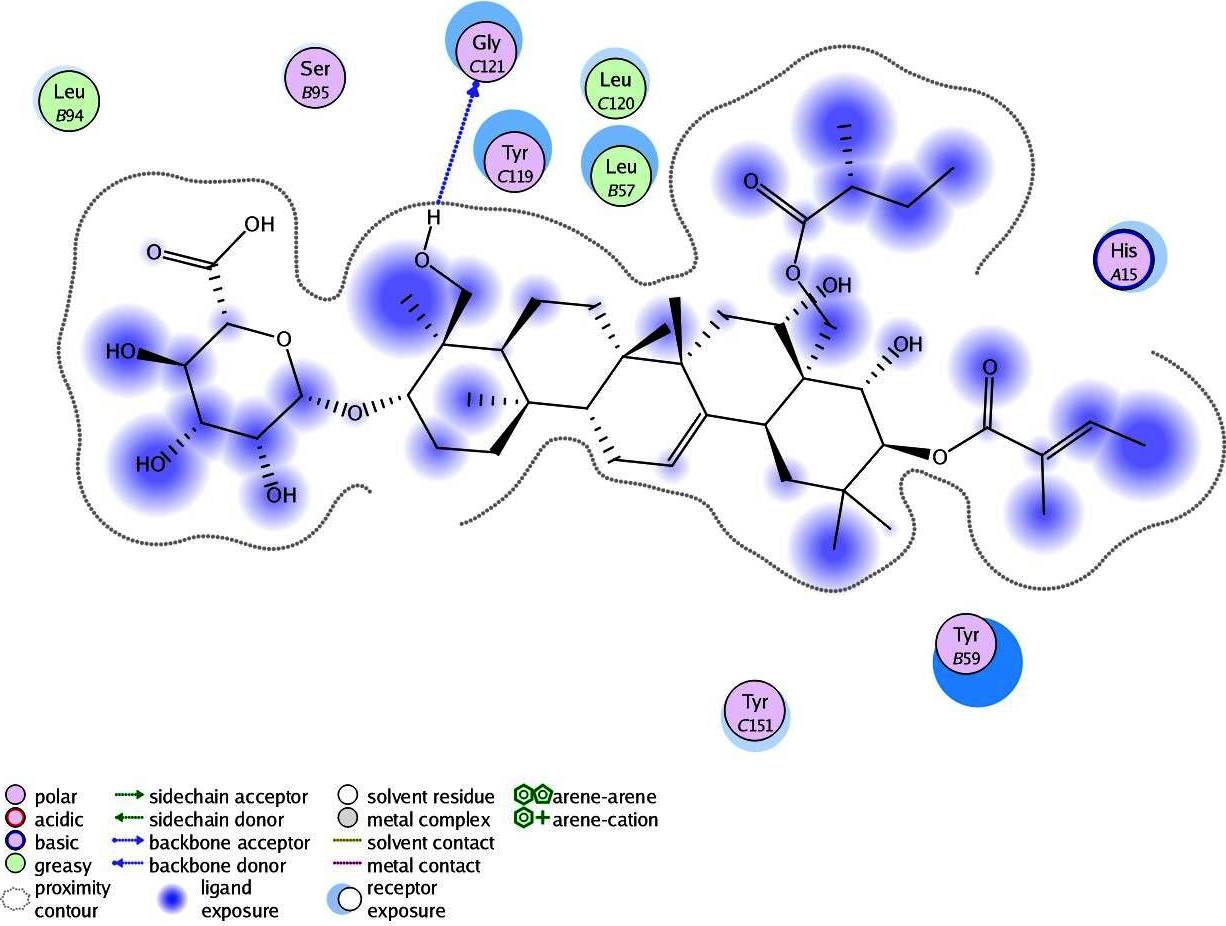 | | |
| 3D overview of Gymnemic acid XII-TNFα complex  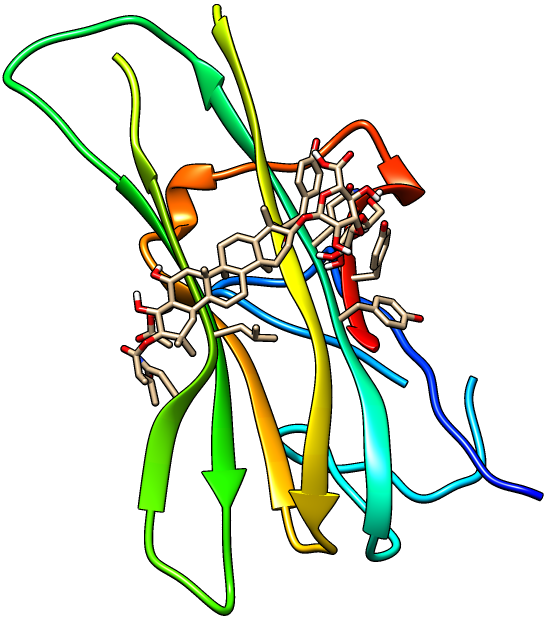 | 2D model of Gymnemic acid XII-TNFα complex showing binding interactions  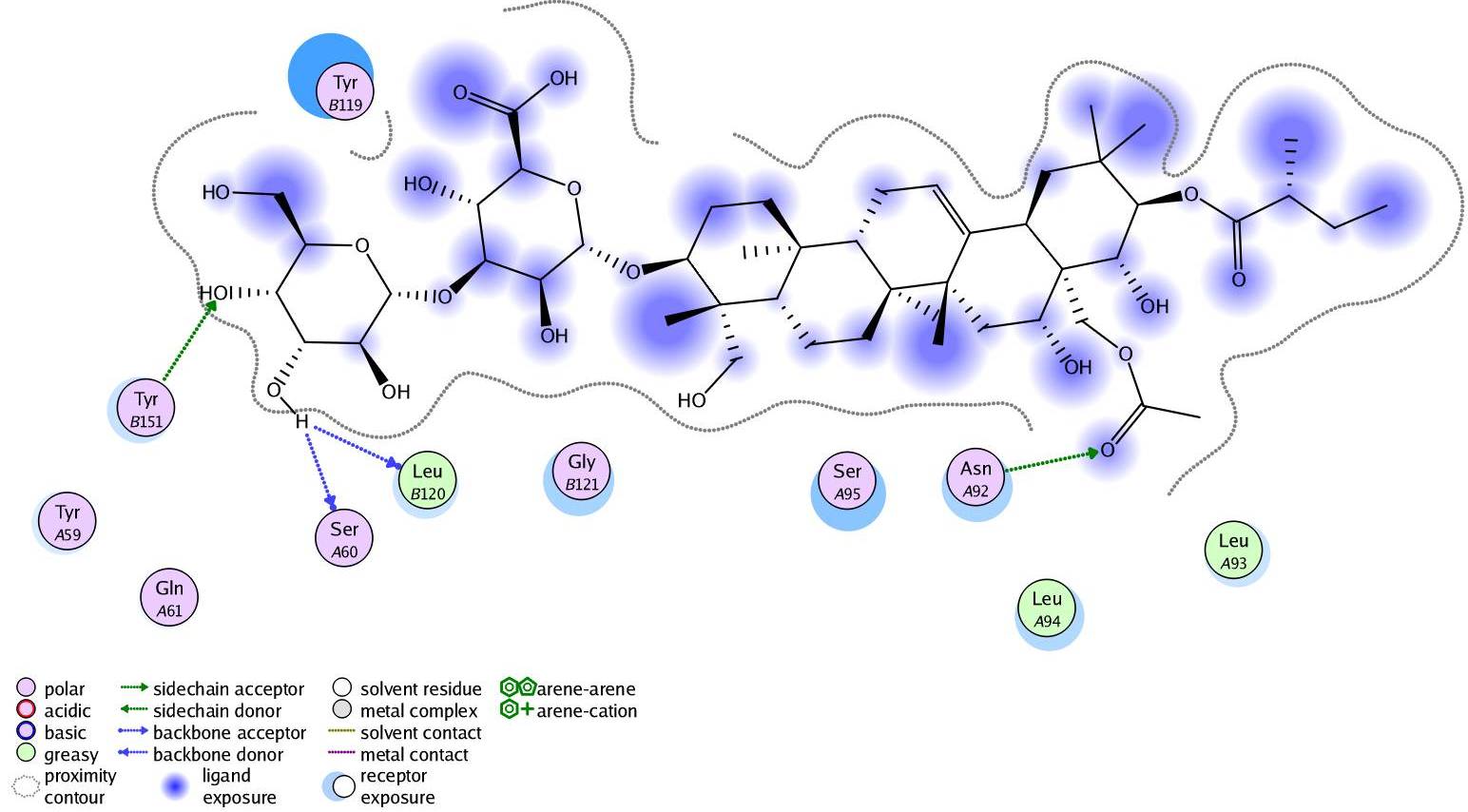 | | | |
| 3D overview of Gymnemic acid XIII-TNFα complex  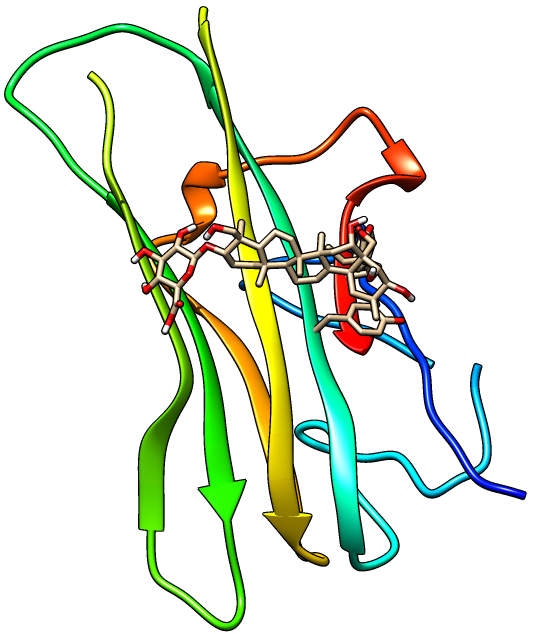 | 2D model of Gymnemic acid XIII-TNFα complex showing binding interactions  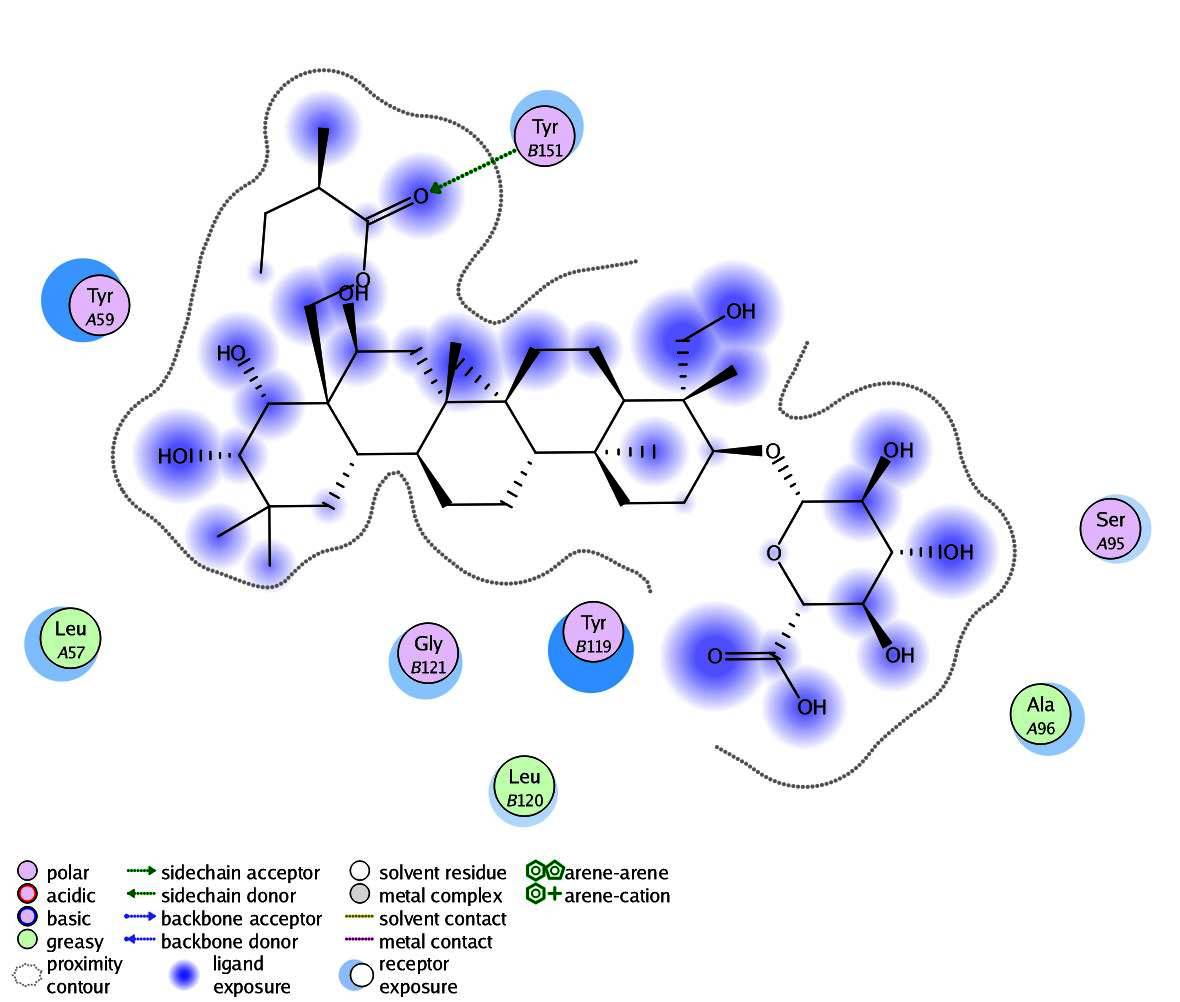 | | | |
| 3D overview of Gymnemic acid XIV-TNFα complex  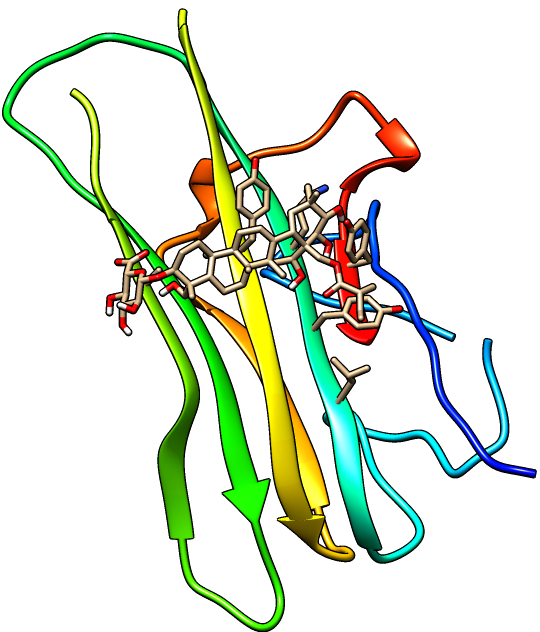 | | | | 2D model of Gymnemic acid XIV-TNFα complex showing binding interactions  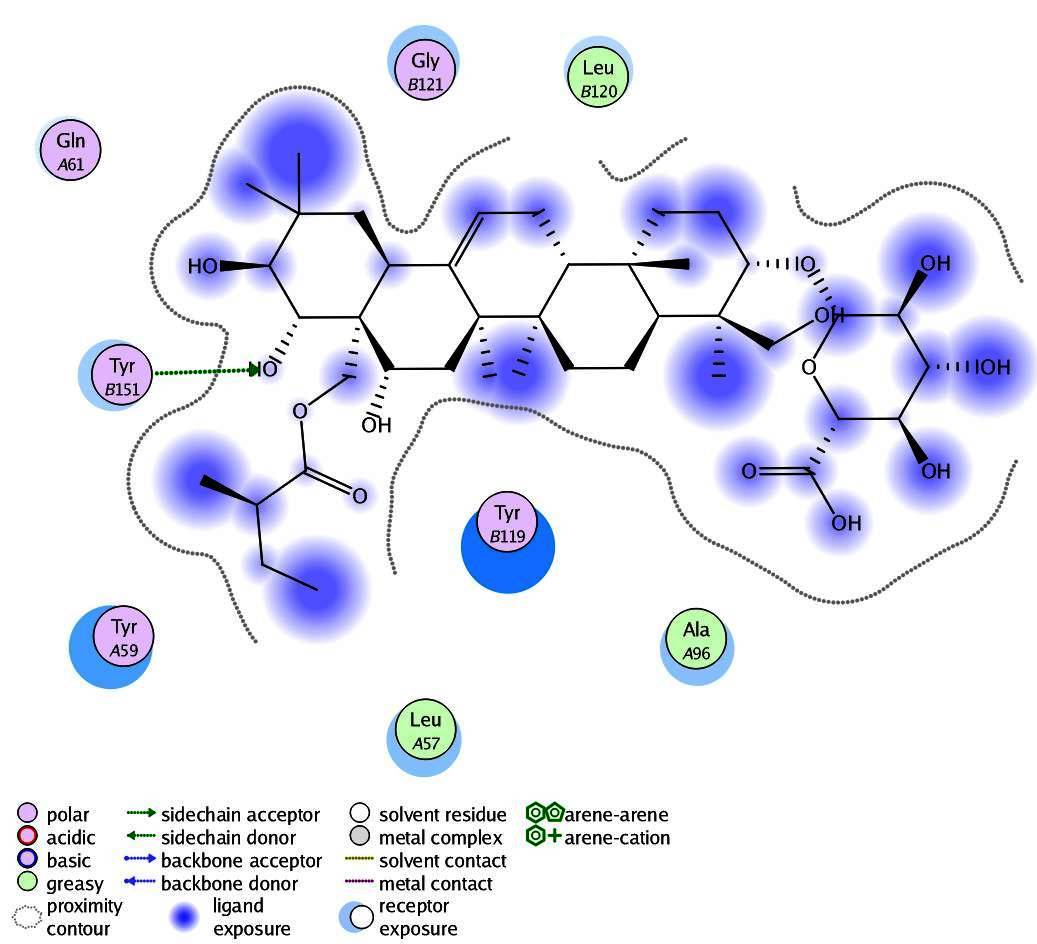 |
