# Supplementary material for: Exploring the therapeutic potential of triterpenoid saponins from Gymnema sylvestre: Mechanistic insights into hepatoprotection, immunomodulation, anticancer activities, molecular docking, and pharmacokinetics
Source: Heliyon. 2024 Nov 30;10(23):e40850. doi: 10.1016/j.heliyon.2024.e40850 (PMC11666954; doi:10.1016/j.heliyon.2024.e40850)
Supplement: Multimedia component 2 [file mmc2.docx]

**Table 1S. Acute toxicity studies of GST in male wistar strain albino rats**

The acute toxicity studies were carried out according to guideline No.420 of the Organization for European Economic Cooperation (OECD) using male Wistar strain albino rats (150-175 gm). GST was administered orally at doses of 100, 200, 400, 600, 800, and 1000 mg/kg body weight for three weeks to evaluate behavioural changes, mortality, and toxicological effects. Doses ranging from 100 to 400 mg/kg did not result in any notable behavioural changes, alterations in body weight, relative organ weights, or kidney and liver function, and no mortality was observed compared to the normal control group. Mild toxicity symptoms were observed at 600 and 800 mg/kg, whereas the 1000 mg/kg dose induced clear signs of toxicity, including behavioural changes, weight loss, reduced organ weights, elevated serum creatinine and alkaline phosphatase levels, and ultimately led to mortality (Table 1). Based on these findings, 600 mg/kg was identified as the dose at which mild toxicity symptoms were observed, while 1000 mg/kg led to mortality. Therefore, the lethal dose (LD50) was estimated to be between 600 mg/kg and 1000 mg/kg. For experimental purposes, a safer dose of 150 mg/kg body weight, equivalent to one-fourth of the 600 mg/kg dose, was selected to minimize toxicity risks during further studies.

| **PARAMETERS** | **Control** | **Experimental Groups**  **(Administered with triterpenoid saponin extract of *G. sylvestre*)** | | | | | |
| --- | --- | --- | --- | --- | --- | --- | --- |
|  |  | **100 mg/kg** **BW** | **200 mg/kg BW** | **400 mg/kg BW** | **600 mg/kg BW** | **800 mg/kg BW** | **1000 mg/kg BW** |
| **Mortality (D/T)** | 0/6 | 0/6 | 0/6 | 0/6 | 0/6 | 0/6 | 1/6 |
| **Behavioral changes** | None | None | None | None | None | Hypo activity | Hypo activity |
| **Body weight**  **(gm)** | 171.41±3.48 | 172.56±4.14 | 171.83±3.86 | 167.17±4.29 | 161.11^*^±3.06 | 154.66^*^±3.82 | 151.18^*^±3.35 |
| **Spleen weight**  **(gm)** | 0.54±0.04 | 0.57±0.05 | 0.53±0.06 | 0.49±0.05 | 0.43^*^±0.04 | 0.34^*^±0.03 | 0.29^*^±0.03 |
| **Thymus weight**  **(gm)** | 0.41±0.03 | 0.43±0.04 | 0.41±0.06 | 0.39±0.06 | 0.34^*^±0.04 | 0.28^*^±0.04 | 0.25^*^±0.03 |
| **Liver weight**  **(gm)** | 5.30±0.68 | 5.37±0.76 | 5.33±0.65 | 5.25±0.59 | 5.01±0.31 | 4.83±0.34 | 4.66±0.21 |
| **Kidney weight**  **(gm)** | 1.21±0.11 | 1.27±0.09 | 1.17±0.06 | 1.09±0.05 | 1.01±0.04 | 0.90±0.03 | 0.81±0.03 |
| **Serum creatinine**  **(mg/dl)** | 0.38±0.04 | 0.36±0.05 | 0.41±0.06 | 0.43±0.06 | 0.56^*^±0.08 | 0.72^*^±0.07 | 0.78^*^±0.09 |
| **Serum ALP**  **(KA units)** | 9.44±0.91 | 9.14±0.89 | 9.28±0.96 | 10.18±1.30 | 10.86^*^±1.59 | 12.92^*^±2.13 | 13.17^*^±1.37 |
