# Supplementary material for: Exploring the therapeutic potential of triterpenoid saponins from Gymnema sylvestre: Mechanistic insights into hepatoprotection, immunomodulation, anticancer activities, molecular docking, and pharmacokinetics
Source: Heliyon. 2024 Nov 30;10(23):e40850. doi: 10.1016/j.heliyon.2024.e40850 (PMC11666954; doi:10.1016/j.heliyon.2024.e40850)
Supplement: Multimedia component 3 [file mmc3.docx]

**Fig. 2S. Docking of triterpenoid saponins of *G. sylvestre* into the binding site of EGFR/HER using AutoDock4.2. Ribbon model represents the 3D interaction of docking. The interacting residues were depicted in circles and hydrogen bonds were as green dashed lines.**

| Gymnemaside A-EGFR  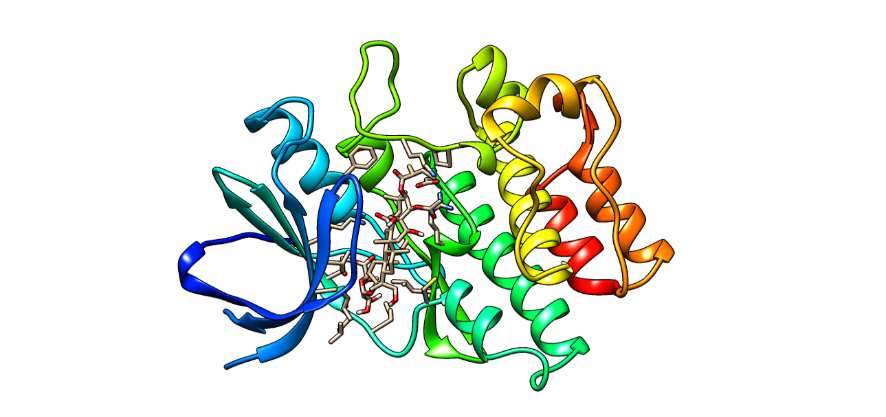 | Gymnemaside A-EGFR  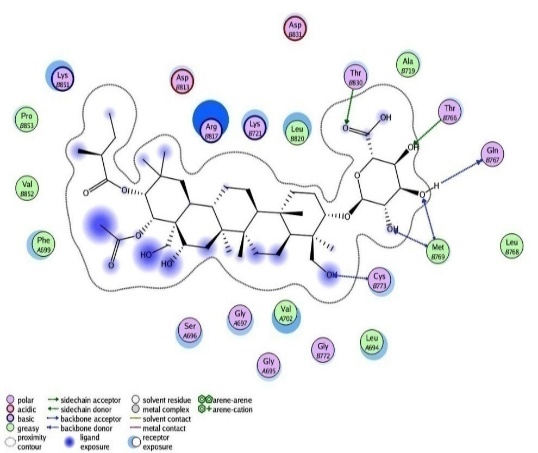 |
| --- | --- |
| Gymnemaside B-EGFR    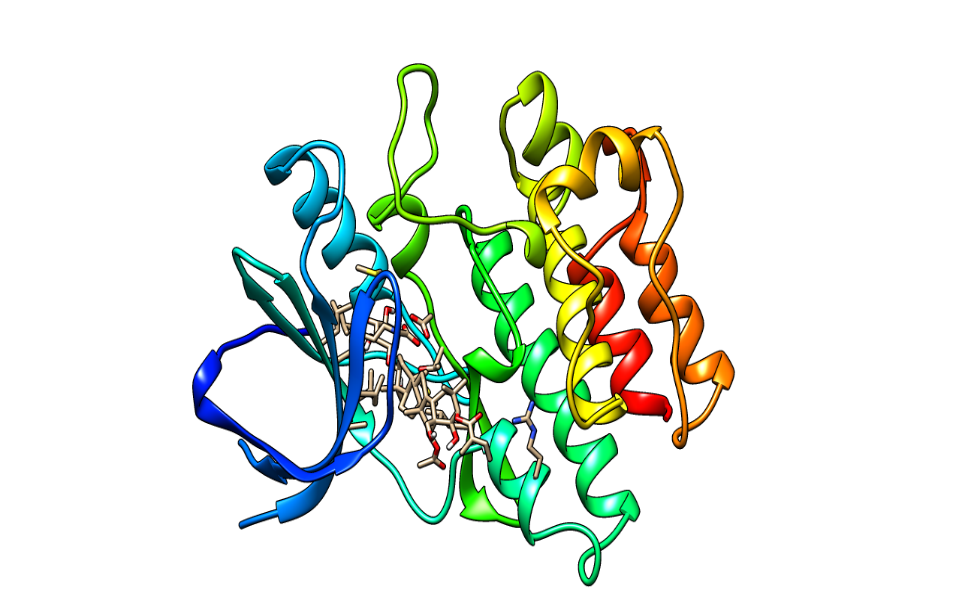 | Gymnemaside B-EGFR  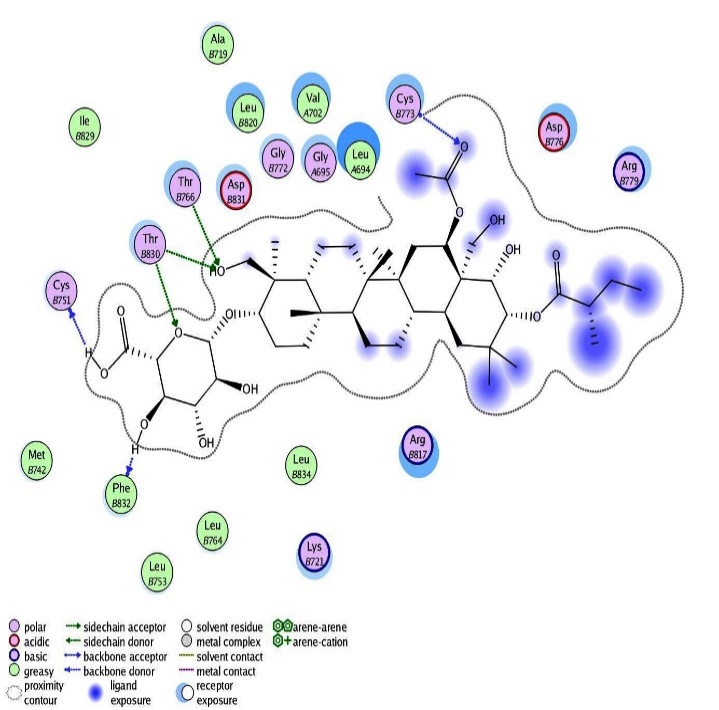 |

**Figure.7 Continued…..**

| Gymnemagenin-EGFR  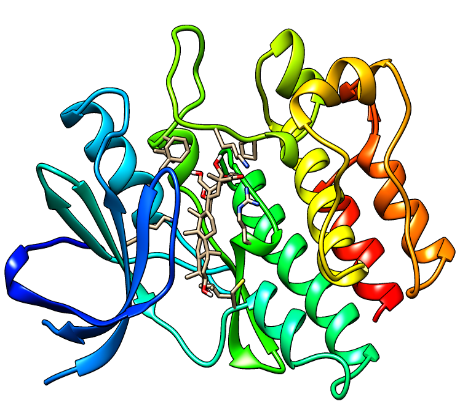 | Gymnemagenin-EGFR  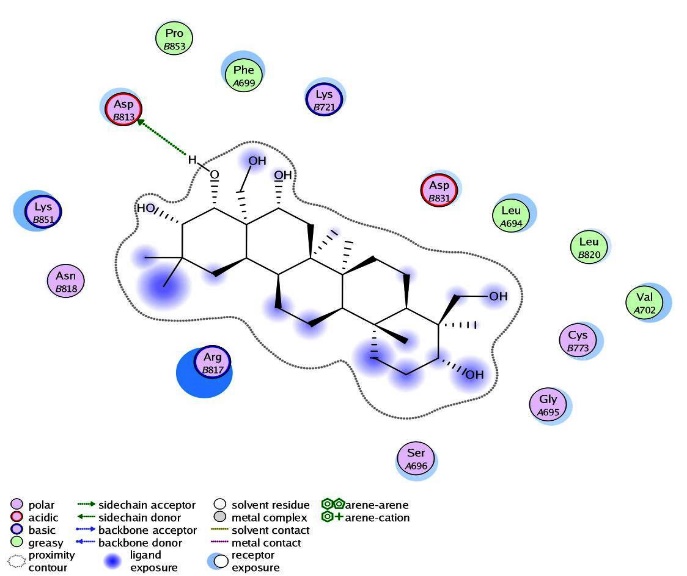 |
| --- | --- |
| Gymnemic acid I-EGFR  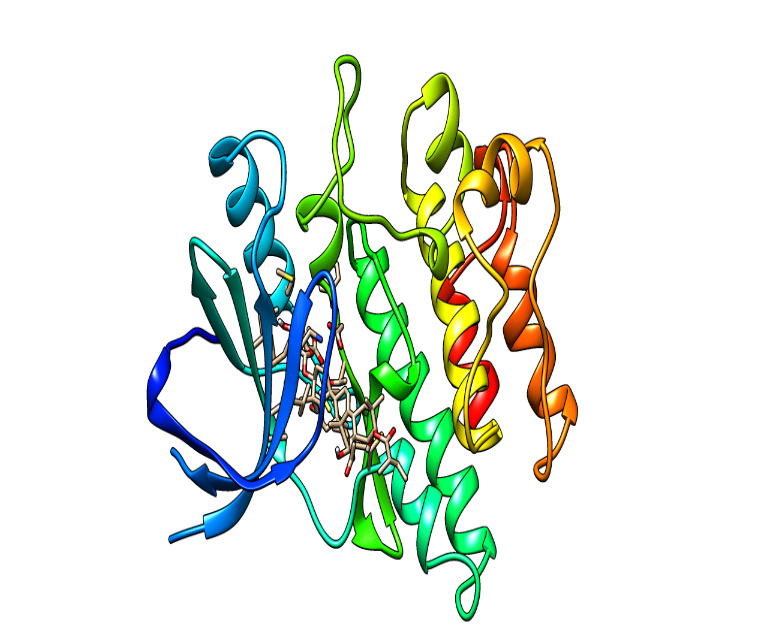  Gymnemic acid II-EGFR | Gymnemic acid I-EGFR  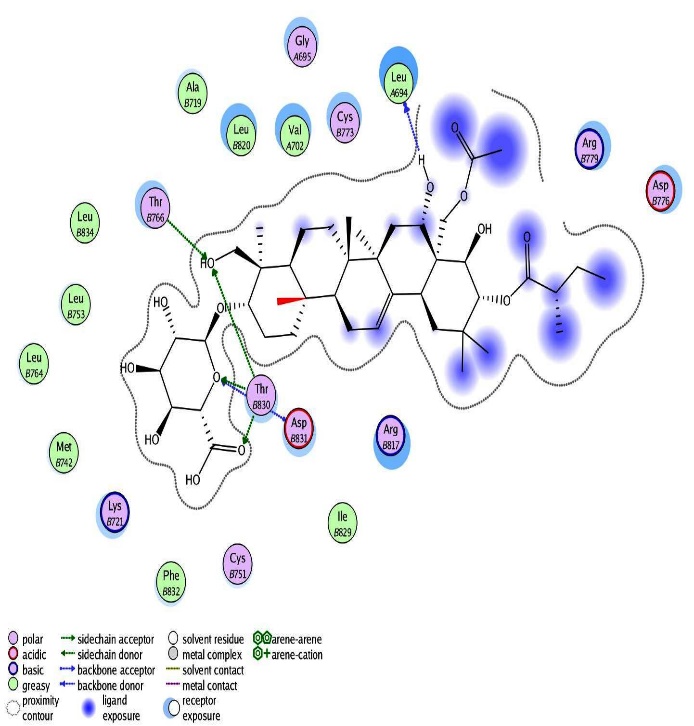  Gymnemic acid II-EGFR |


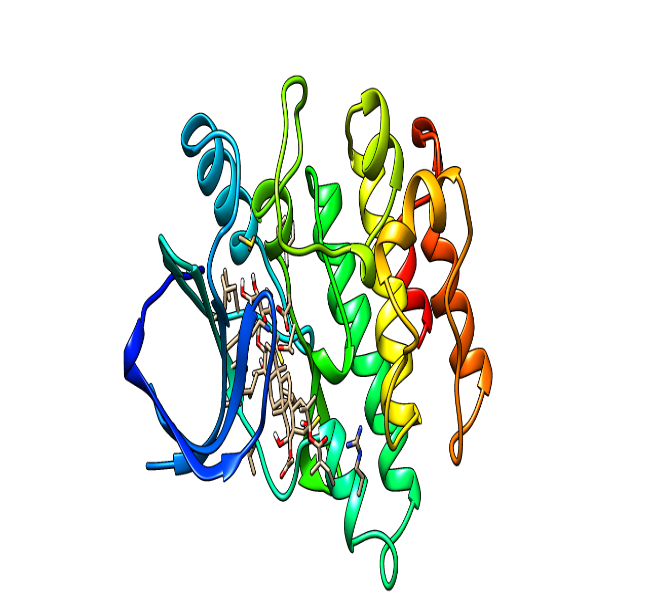

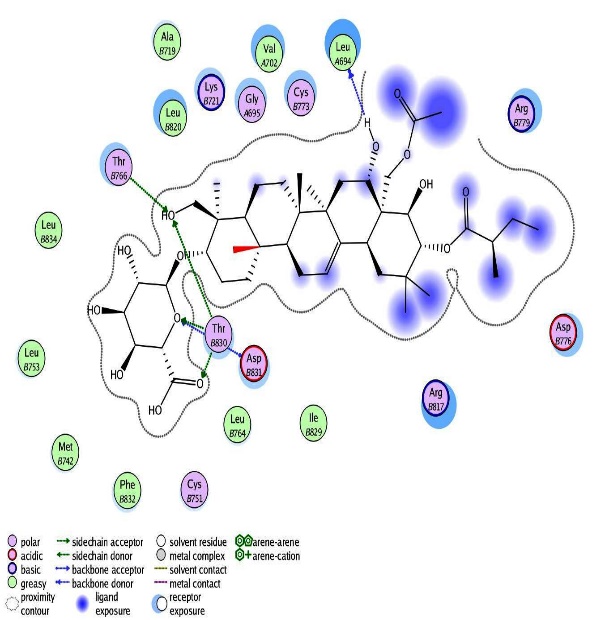


**Figure.7 Continued…..**

| Gymnemic acid III-EGFR  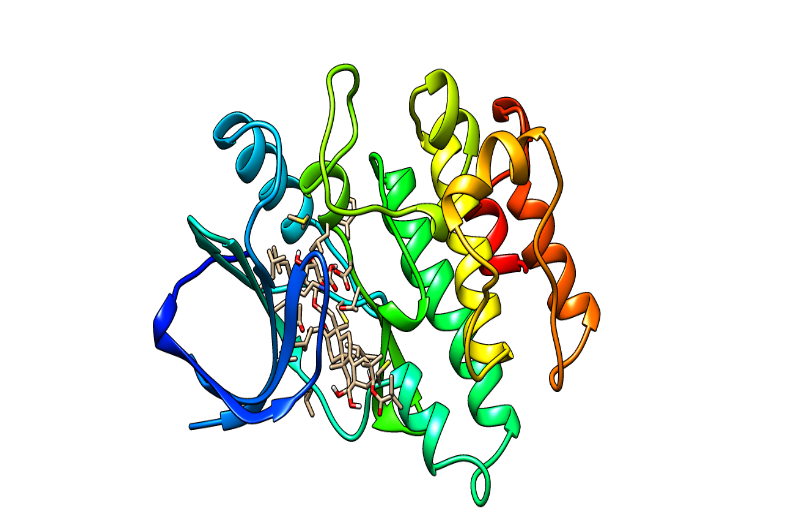  Gymnemic acid IV-EGFR | Gymnemic acid III-EGFR  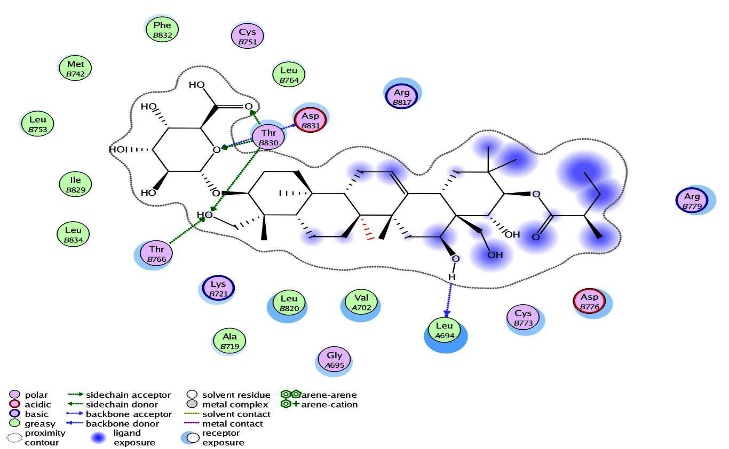  Gymnemic acid IV-EGFR |
| --- | --- |


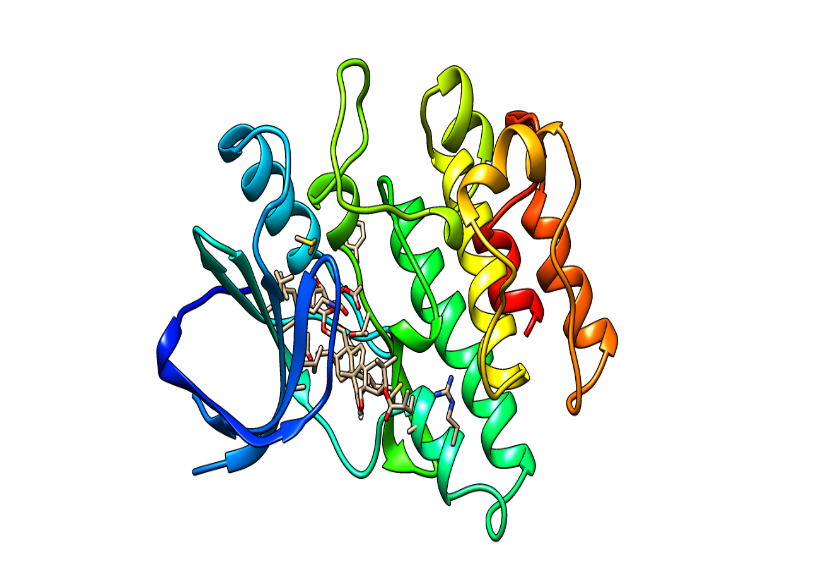

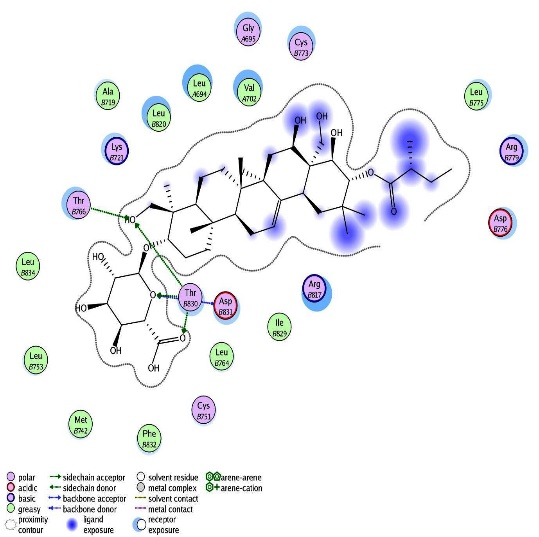


Gymnemic acid V-EGFR Gymnemic acid V-EGFR


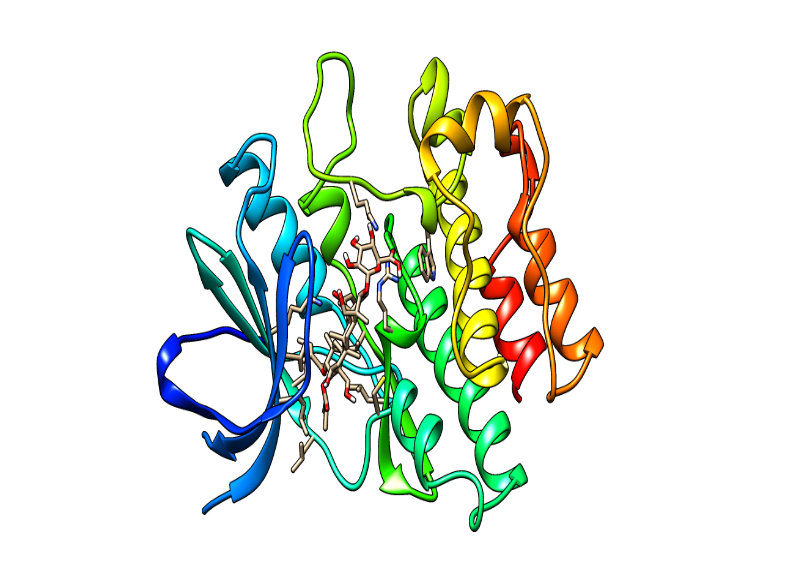

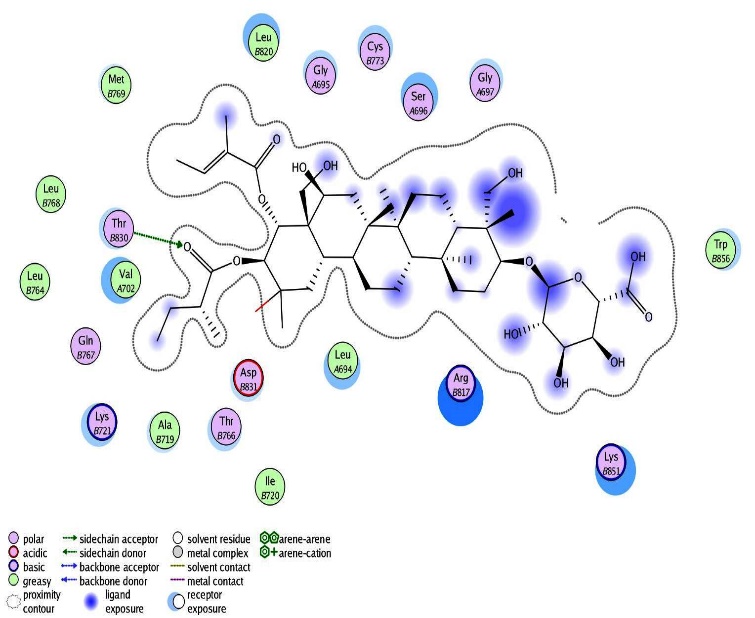
 **Figure.7 Continued…..**

Gymnemic acid VI-EGFR Gymnemic acid VI-EGFR


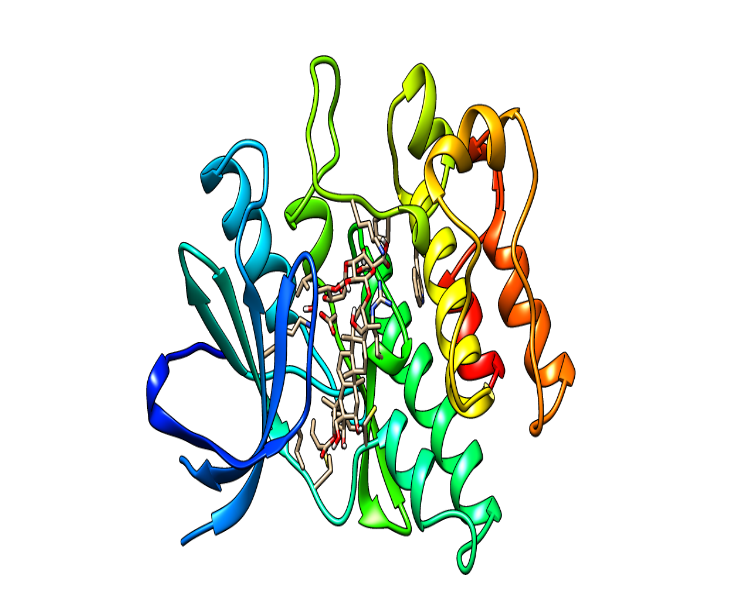

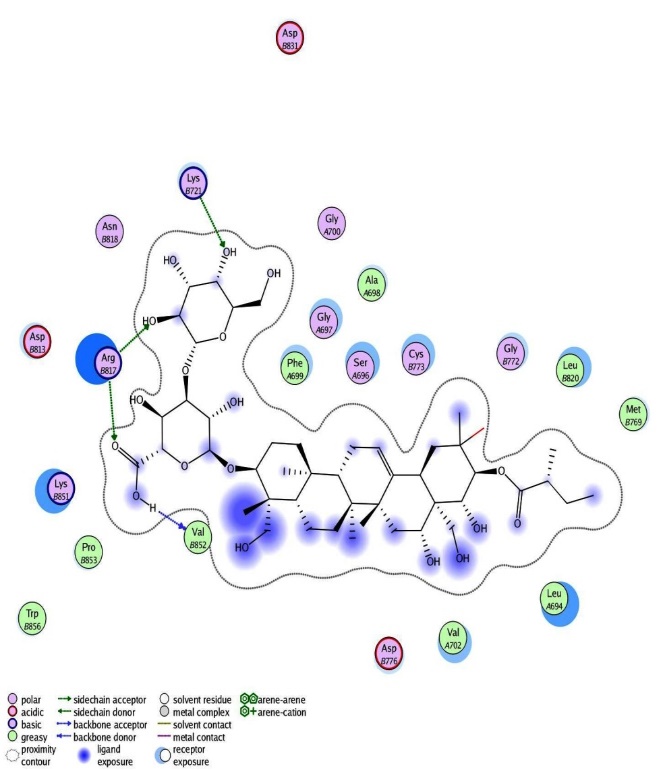


| Gymnemic acid VII-EGFR  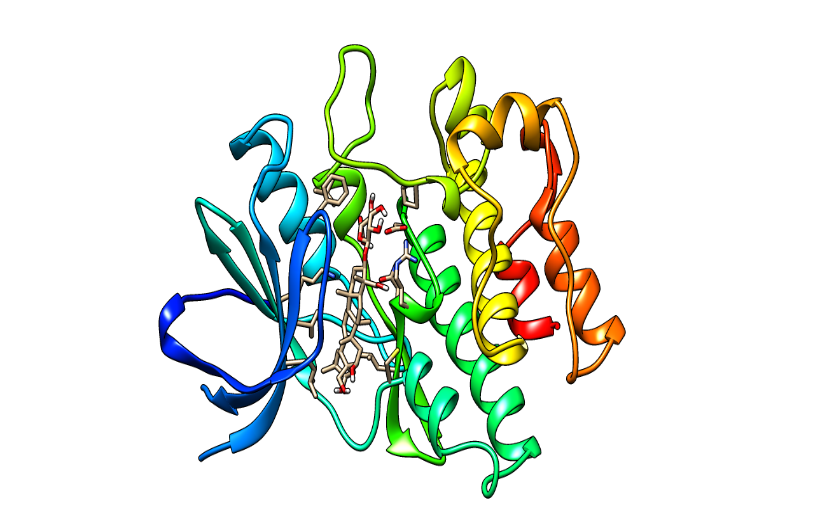 | Gymnemic acid VII-EGFR  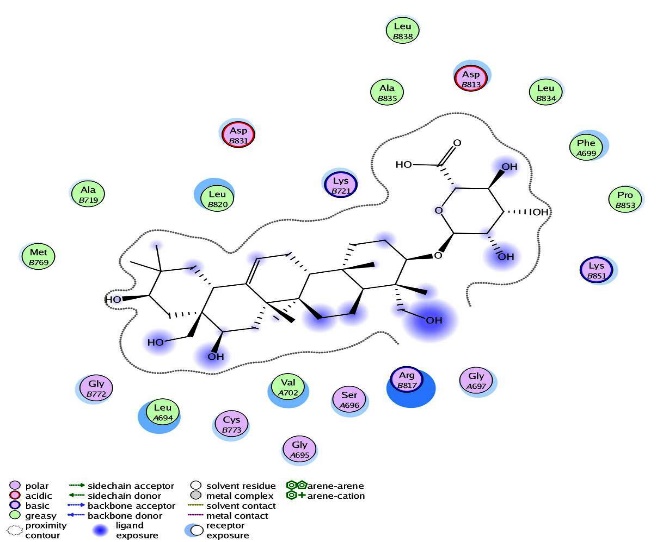 |
| --- | --- |

Gymnemic acid VIII-EGFR Gymnemic acid VIII-EGFR


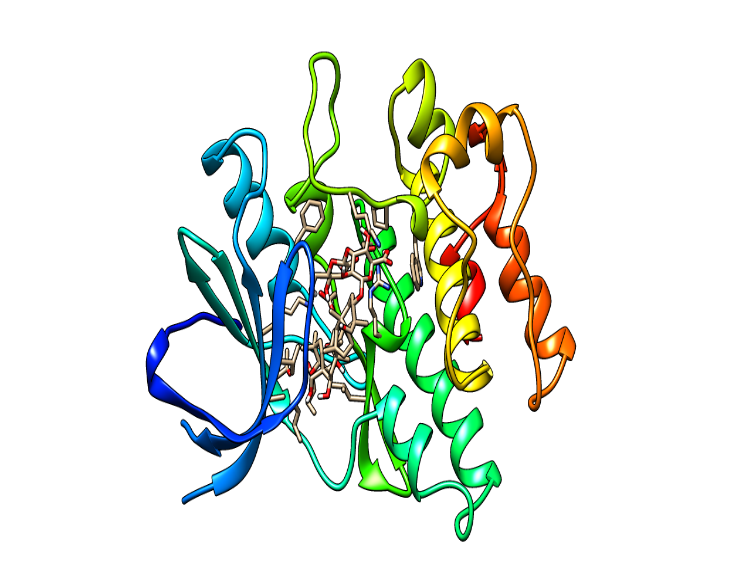

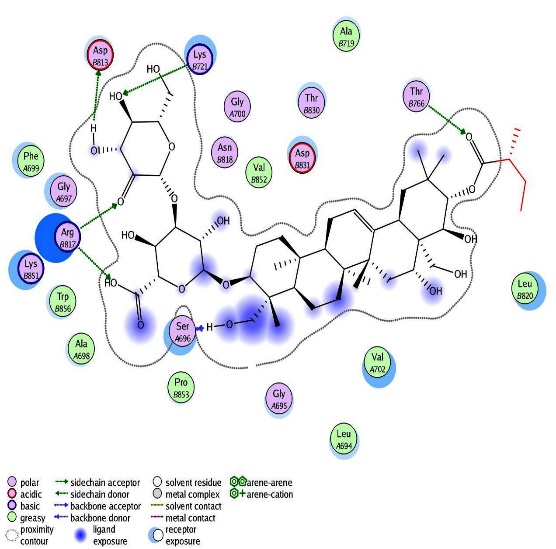


**Figure.7 Continued…..**

| Gymnemic acid IX-EGFR  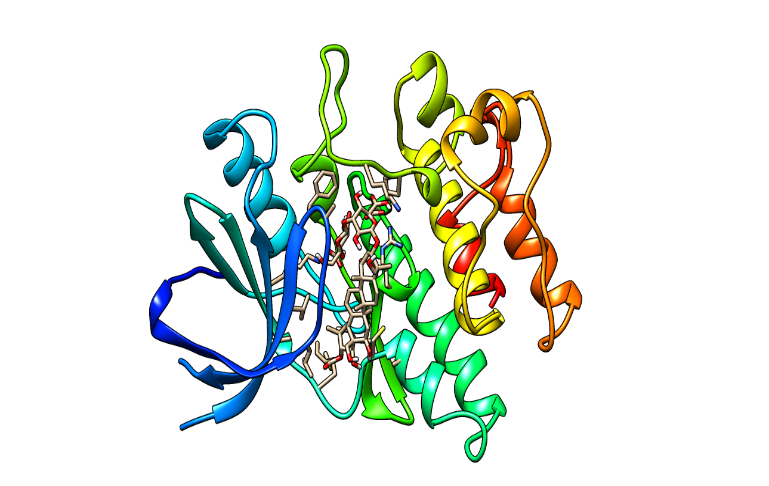 | Gymnemic acid IX-EGFR  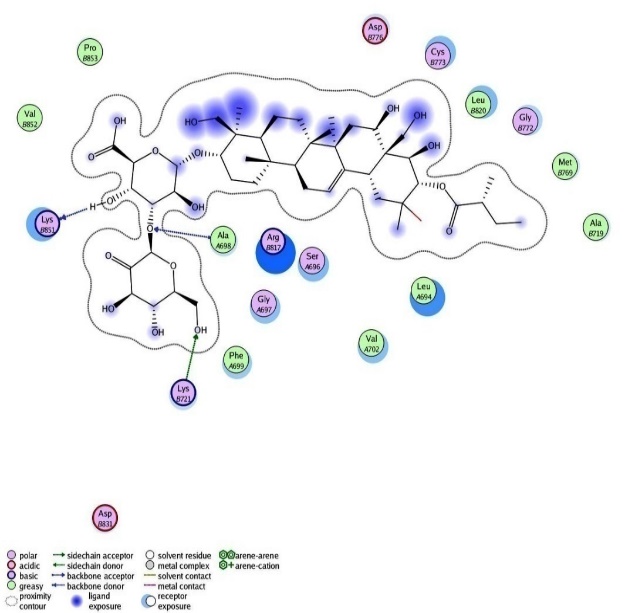 |
| --- | --- |

| Gymnemic acid X-EGFR  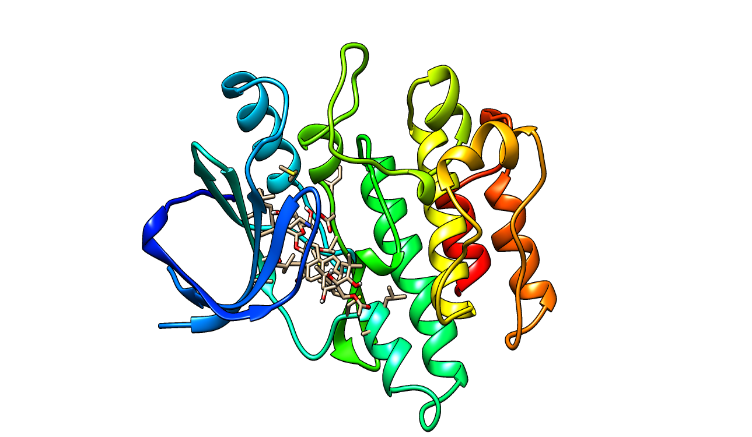 | Gymnemic acid X-EGFR  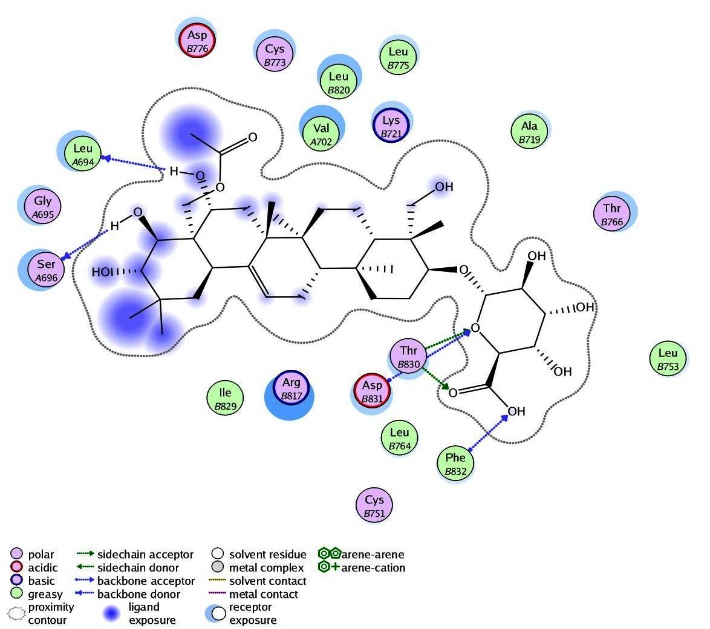 |
| --- | --- |
| Gymnemic acid XI-EGFR  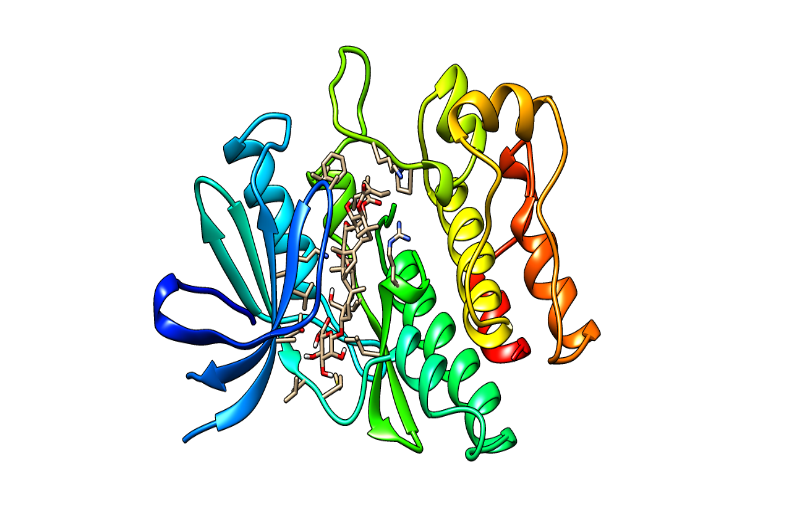 | Gymnemic acid XI-EGFR  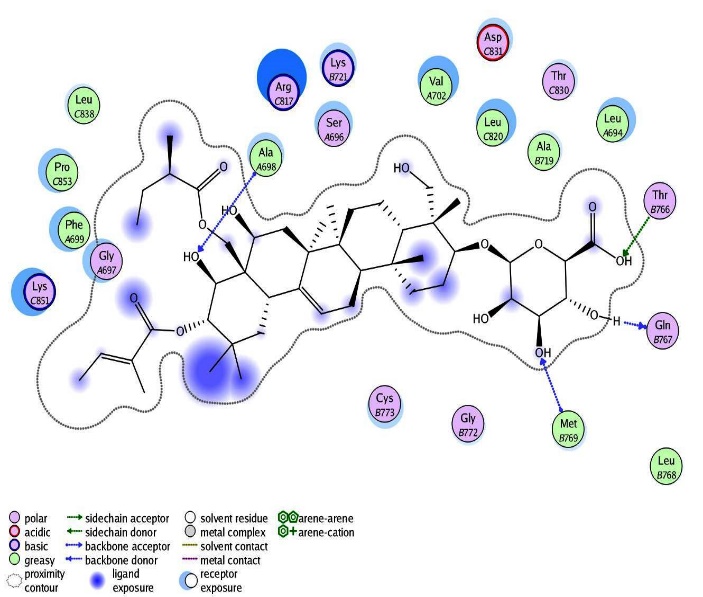 |

**Figure.7 Continued…..**

Gymnemic acid XII-EGFR Gymnemic acid XII-EGFR

**
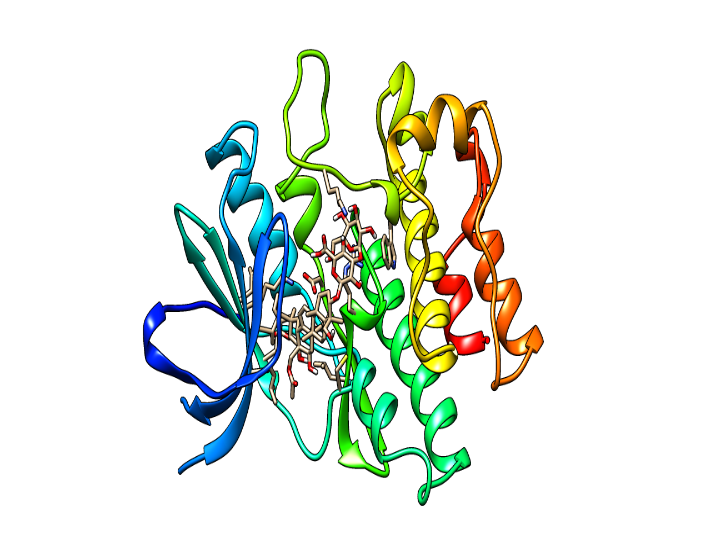

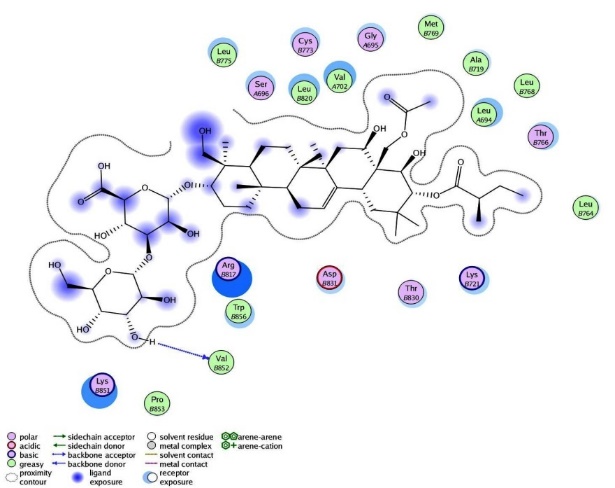
**

| Gymnemic acid XIII-EGFR  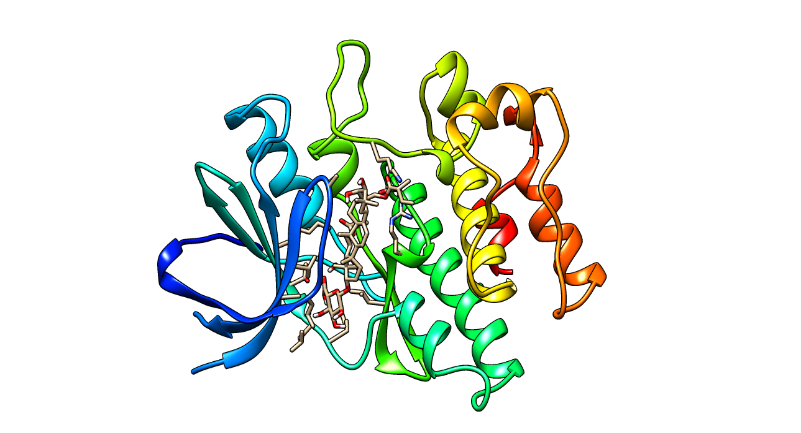 | Gymnemic acid XIII-EGFR  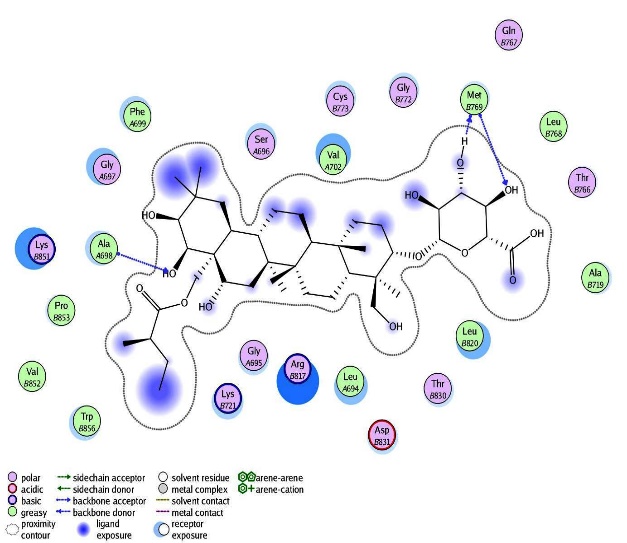 |
| --- | --- |

| Gymnemic acid XIV-EGFR  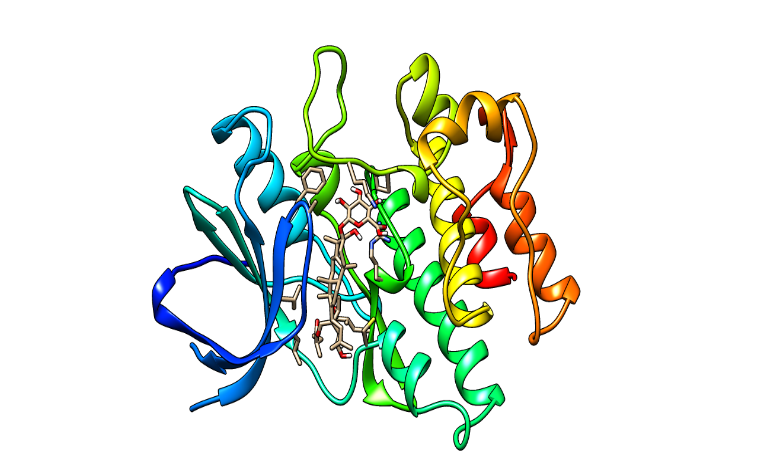 | Gymnemic acid XIV-EGFR  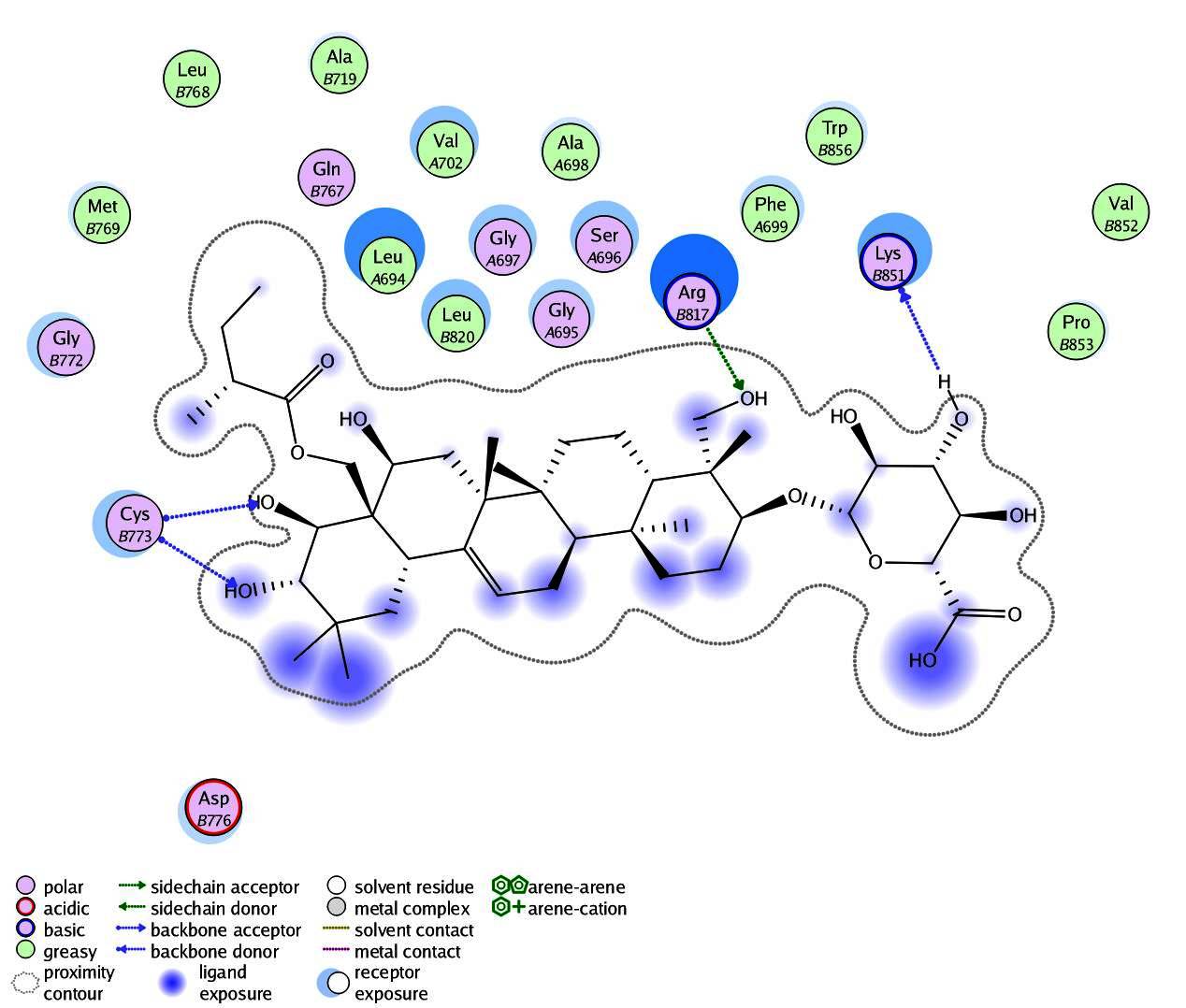 | |
| --- | --- | --- |
| **Fig. 3S. Docking of triterpenoid saponins of *G. sylvestre* into the binding site of HER-2 using AutoDock4.2. Ribbon model represents the 3D interaction of docking. The interacting residues were depicted in circles and hydrogen bonds were as green dashed lines.**   \| Gymnemaside A-HER 2  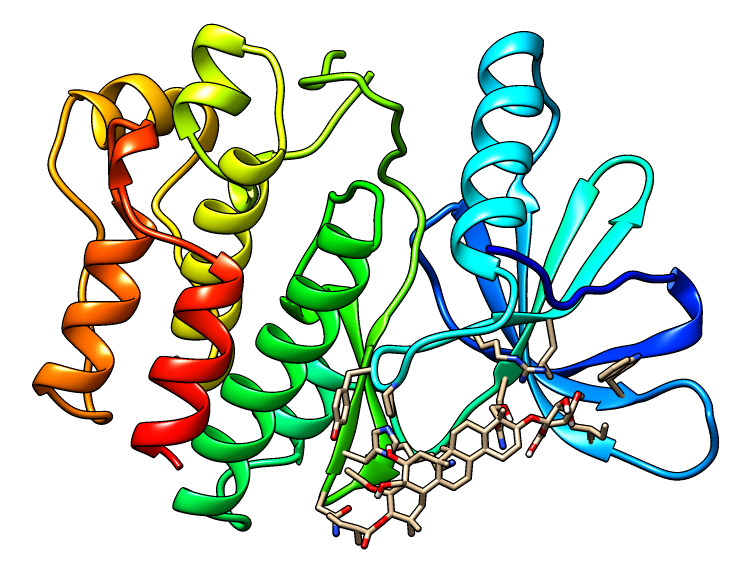 \| Gymnemaside A-HER 2  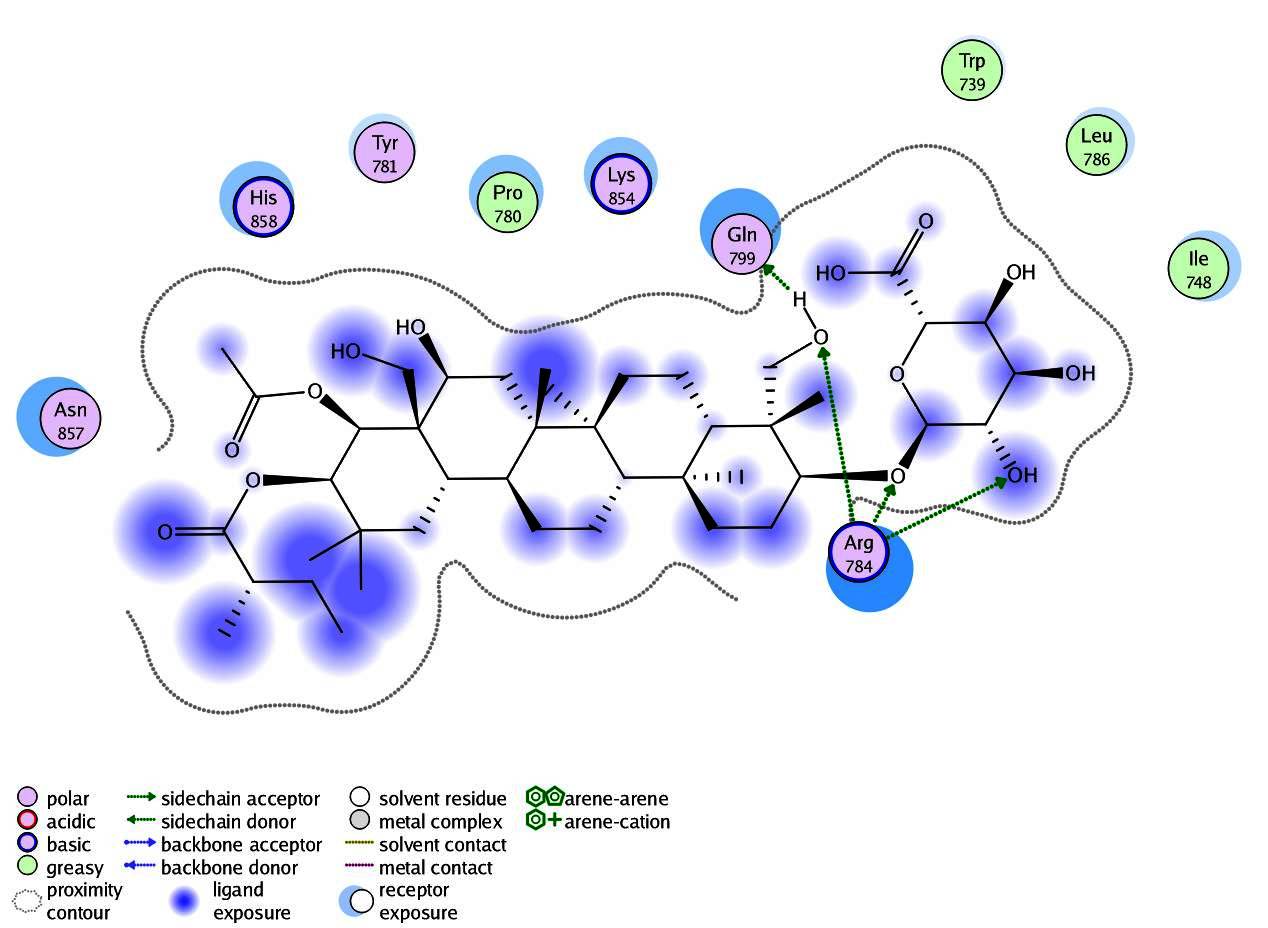 \| \| --- \| --- \| | | |
| Gymnemaside B-HER2  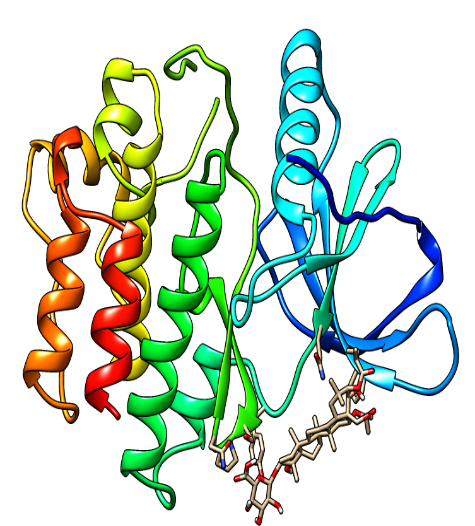 | | Gymnemaside B-HER2  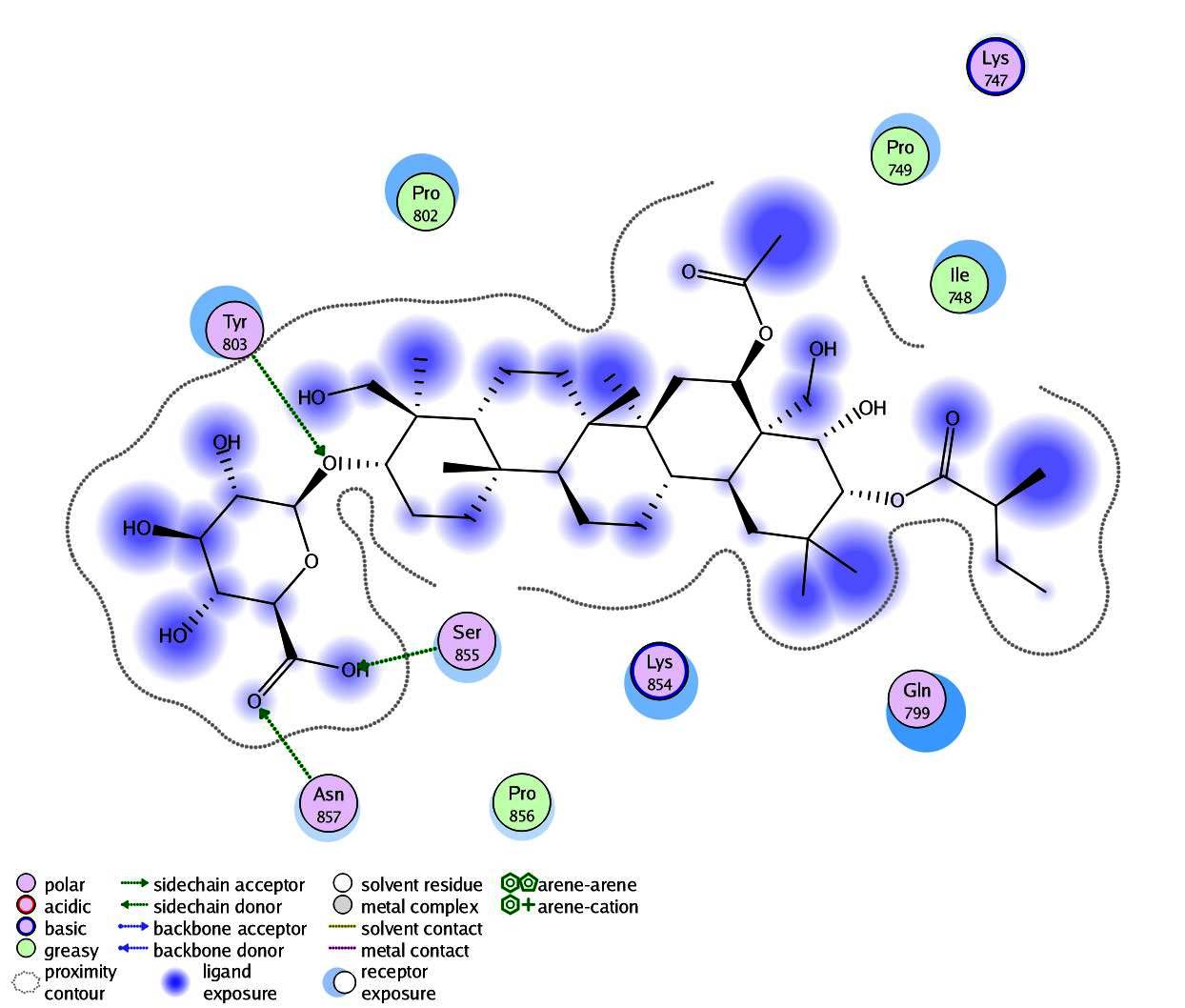 |

**Figure.8 Continued…..**

| Gymnemagenin-HER2  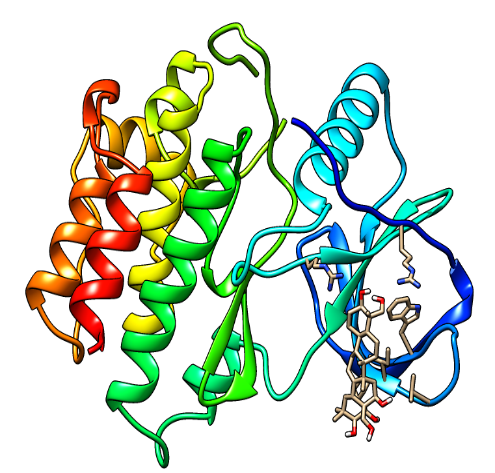 | Gymnemagenin-HER2  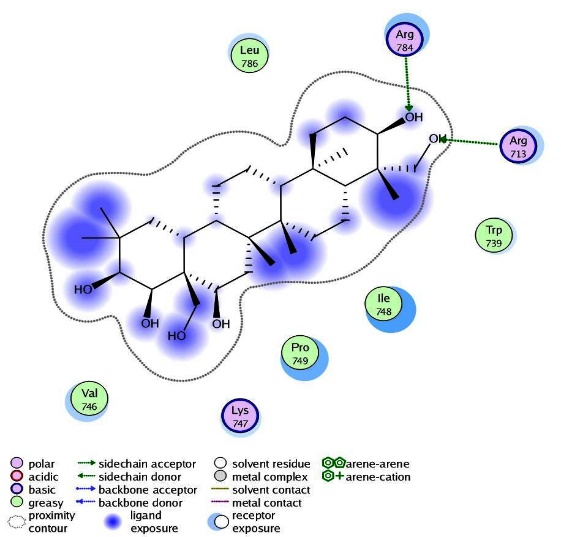 | |
| --- | --- | --- |
| Gymnemic acid I-HER2  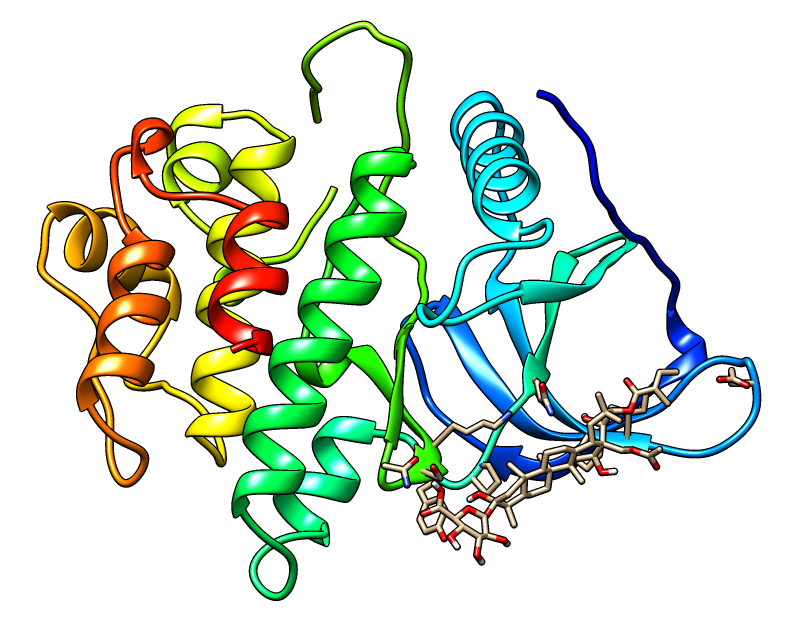 | Gymnemic acid I-HER2  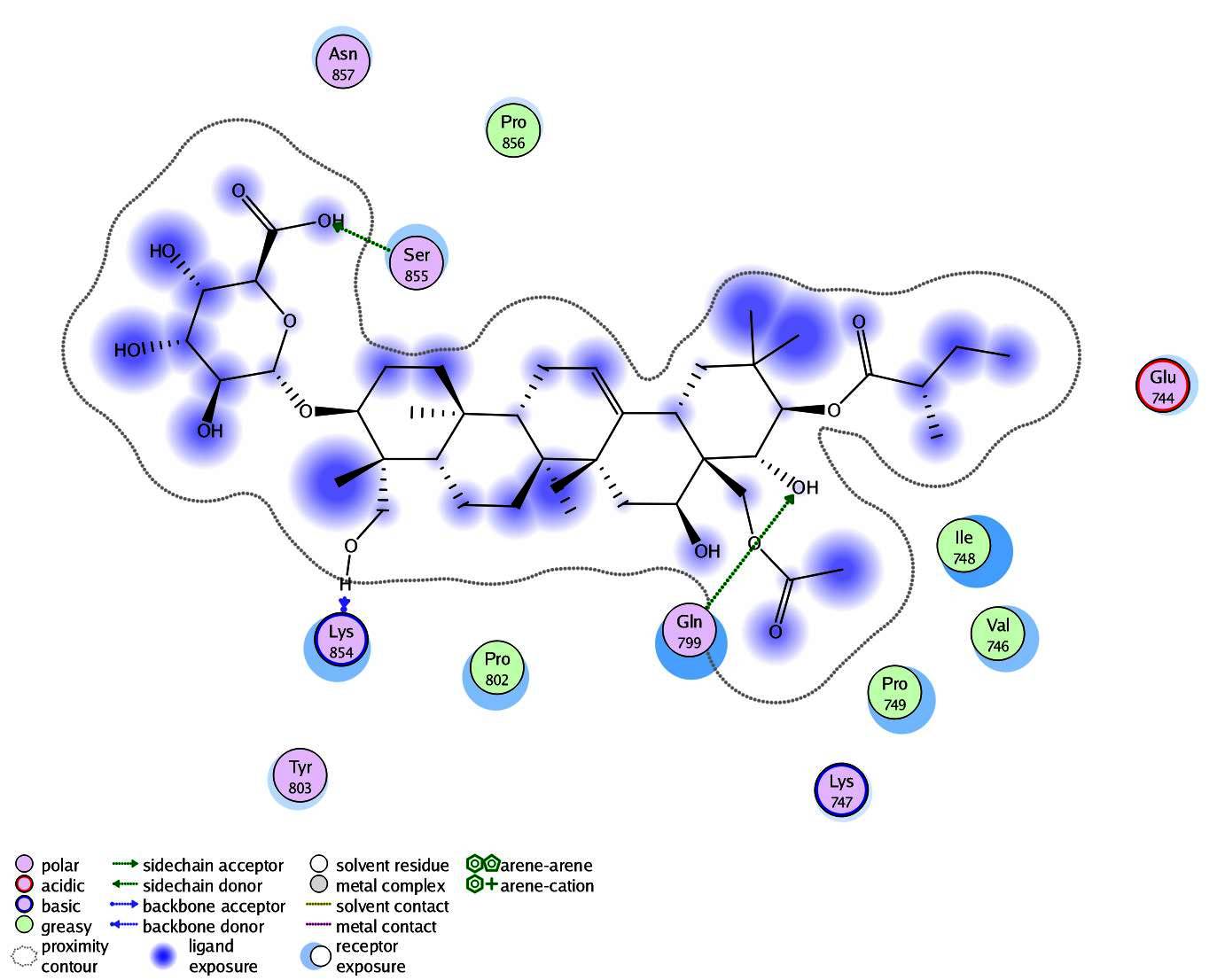 | |
| Gymnemic acid II-HER2  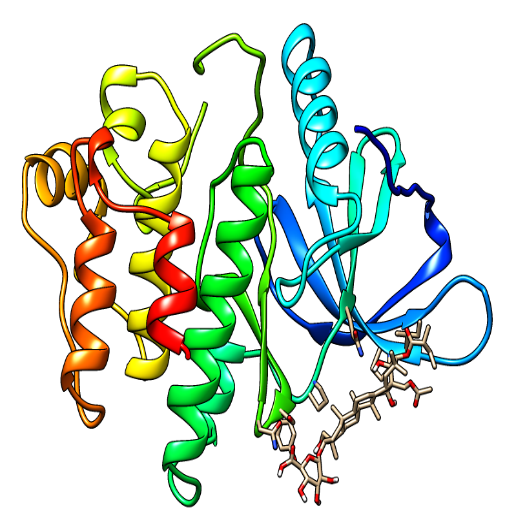 | | Gymnemic acid II-HER2  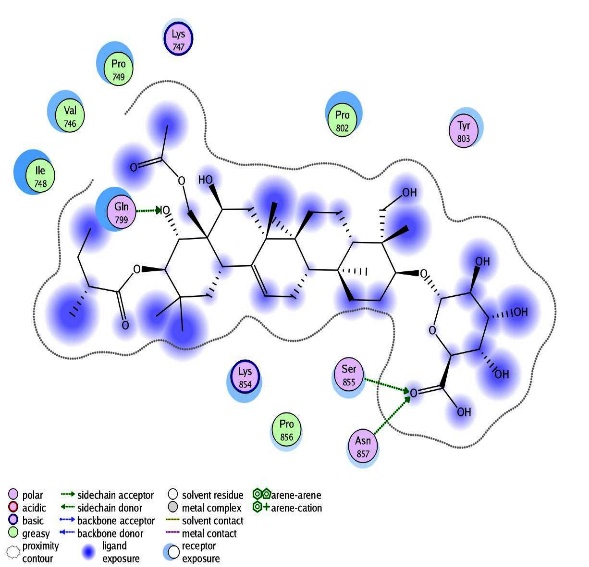  **Figure.8 Continued…..** |
| Gymnemic acid III-HER2  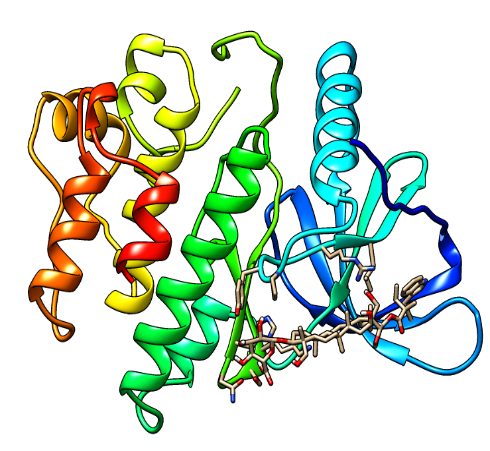 | | Gymnemic acid III-HER2    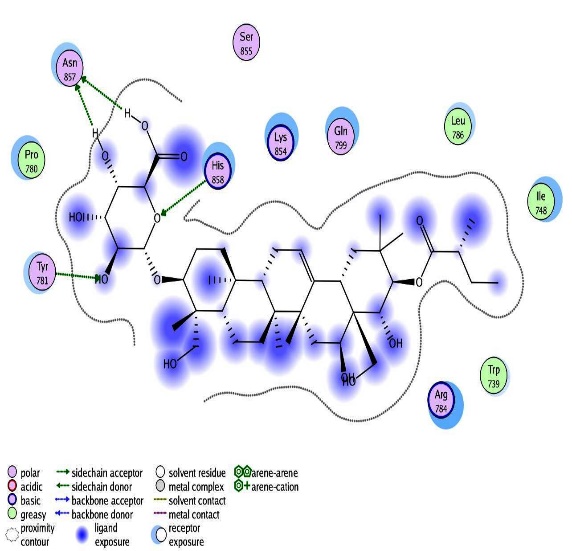 |

| Gymnemic acid IV-HER2  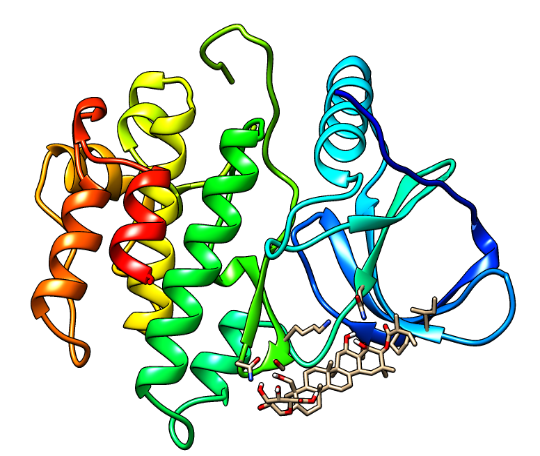 | Gymnemic acid IV-HER2  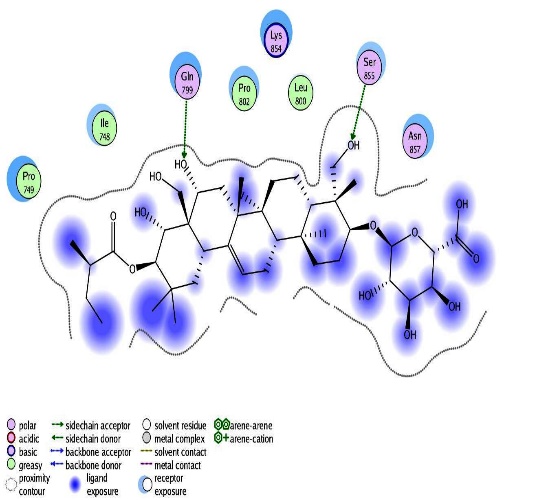 |
| --- | --- |
| Gymnemic acid V-HER2  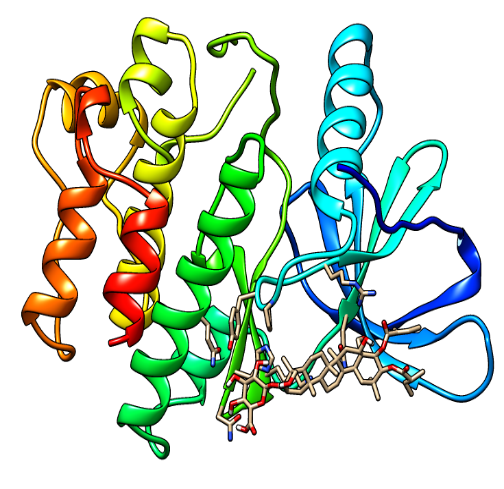 | Gymnemic acid V-HER2  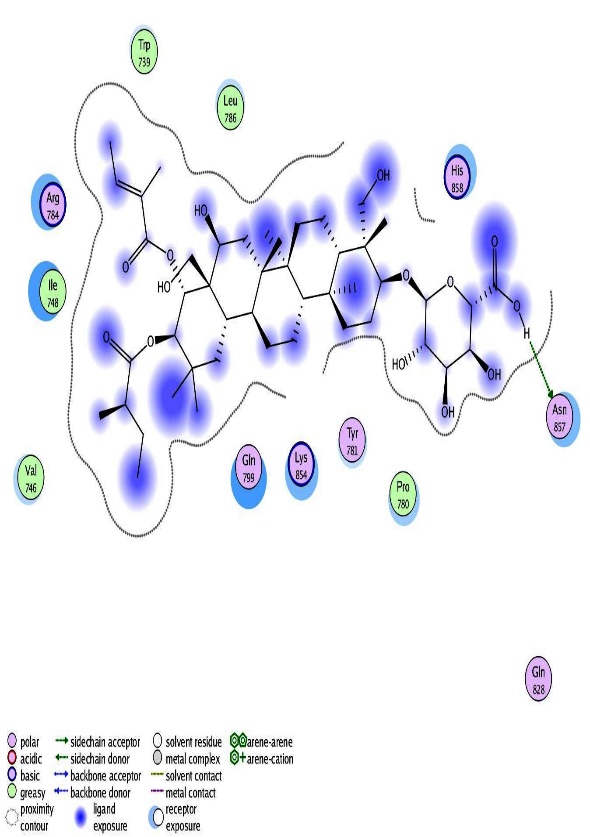 |

**Figure.8 Continued…..**

| Gymnemic acid VI-HER2  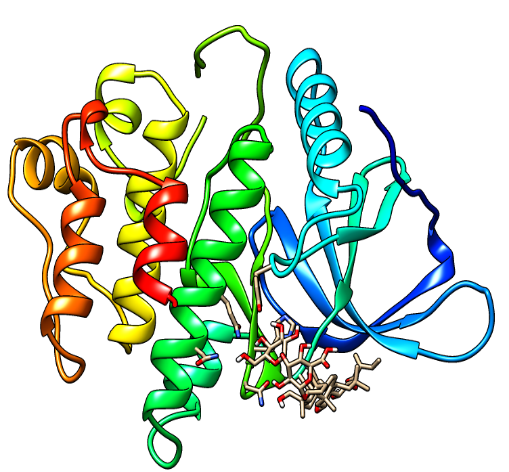  Gymnemic acid VII-HER2 | | Gymnemic acid VI-HER2  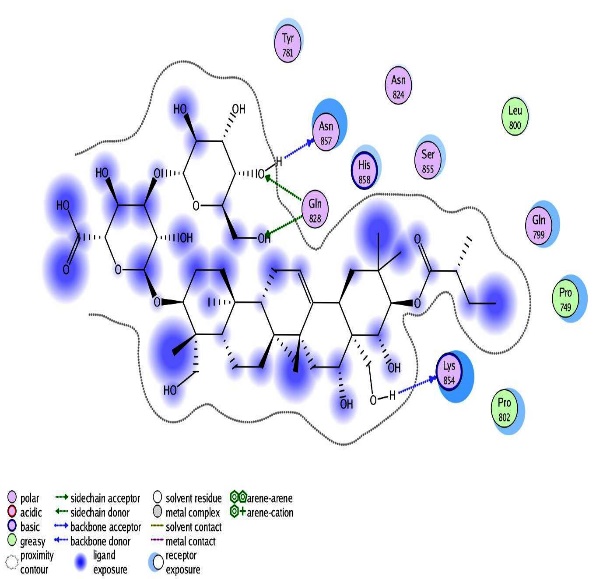  Gymnemic acid VII-HER2 | |
| --- | --- | --- | --- |
| 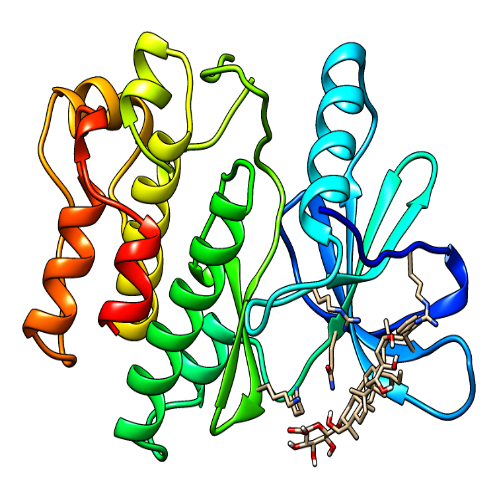 | | 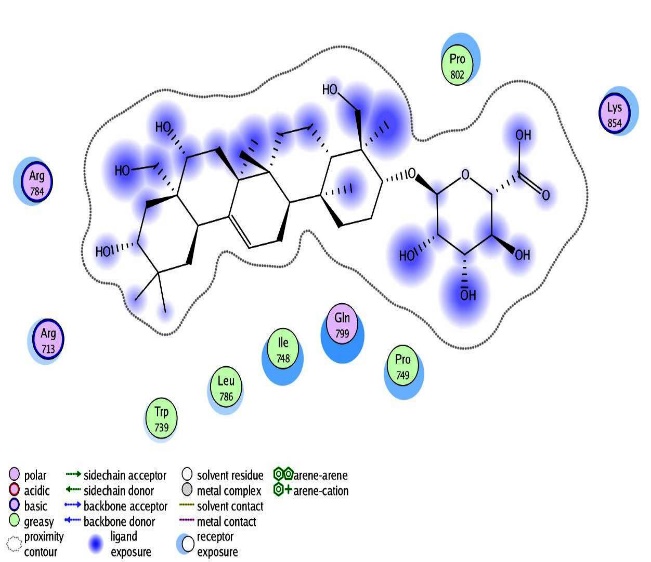 | |
| Gymnemic acid VIII-HER2  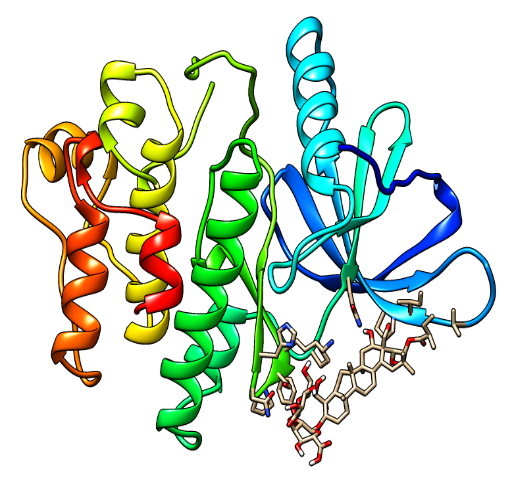 | Gymnemic acid VIII-HER2  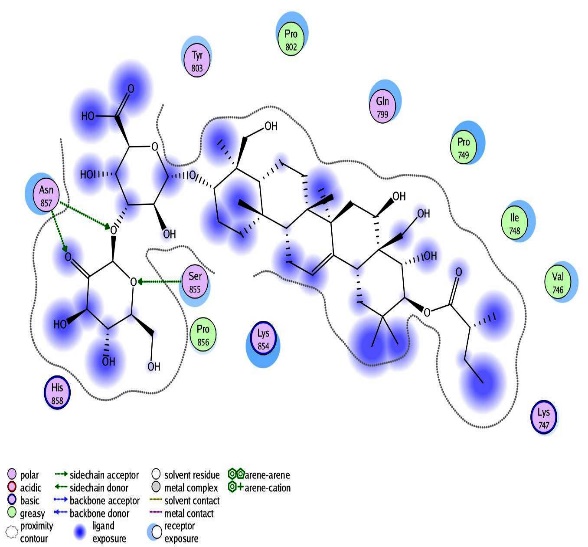  **Figure.8 Continued…..** | |  |
| Gymnemic acid IX-HER2  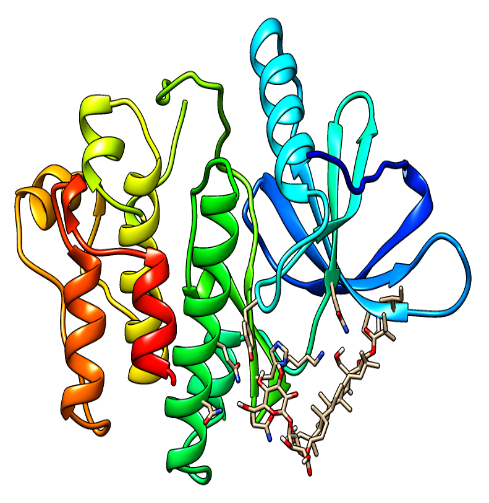 | Gymnemic acid IX-HER2  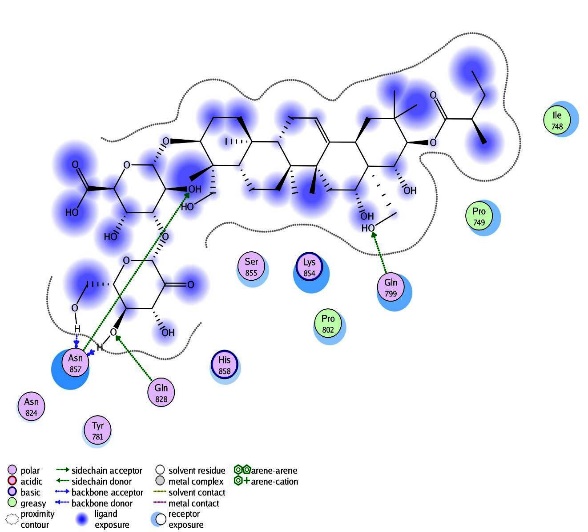 | |  |

| Gymnemic acid X-HER2  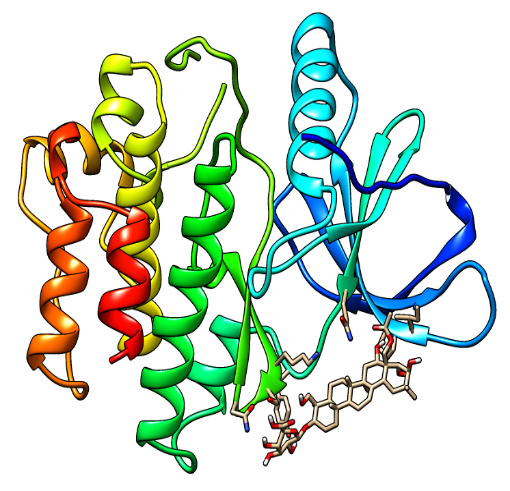 | | Gymnemic acid X-HER2  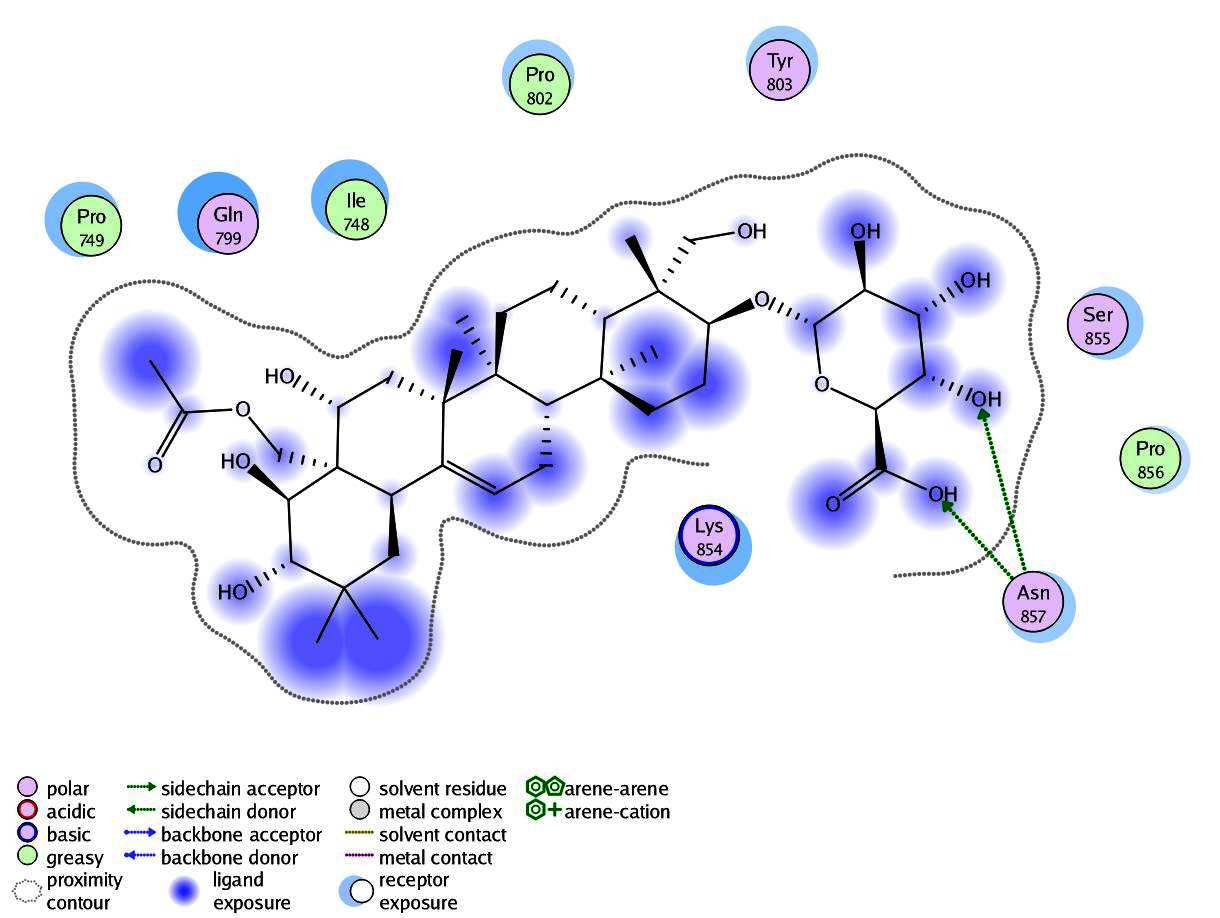 |  |
| --- | --- | --- | --- |
| Gymnemic acid XI-HER2  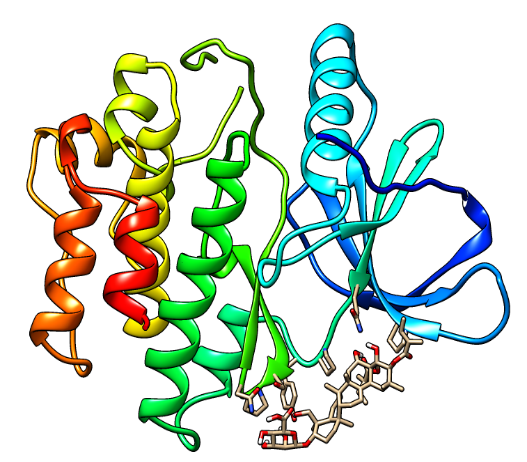 | | Gymnemic acid XI-HER2  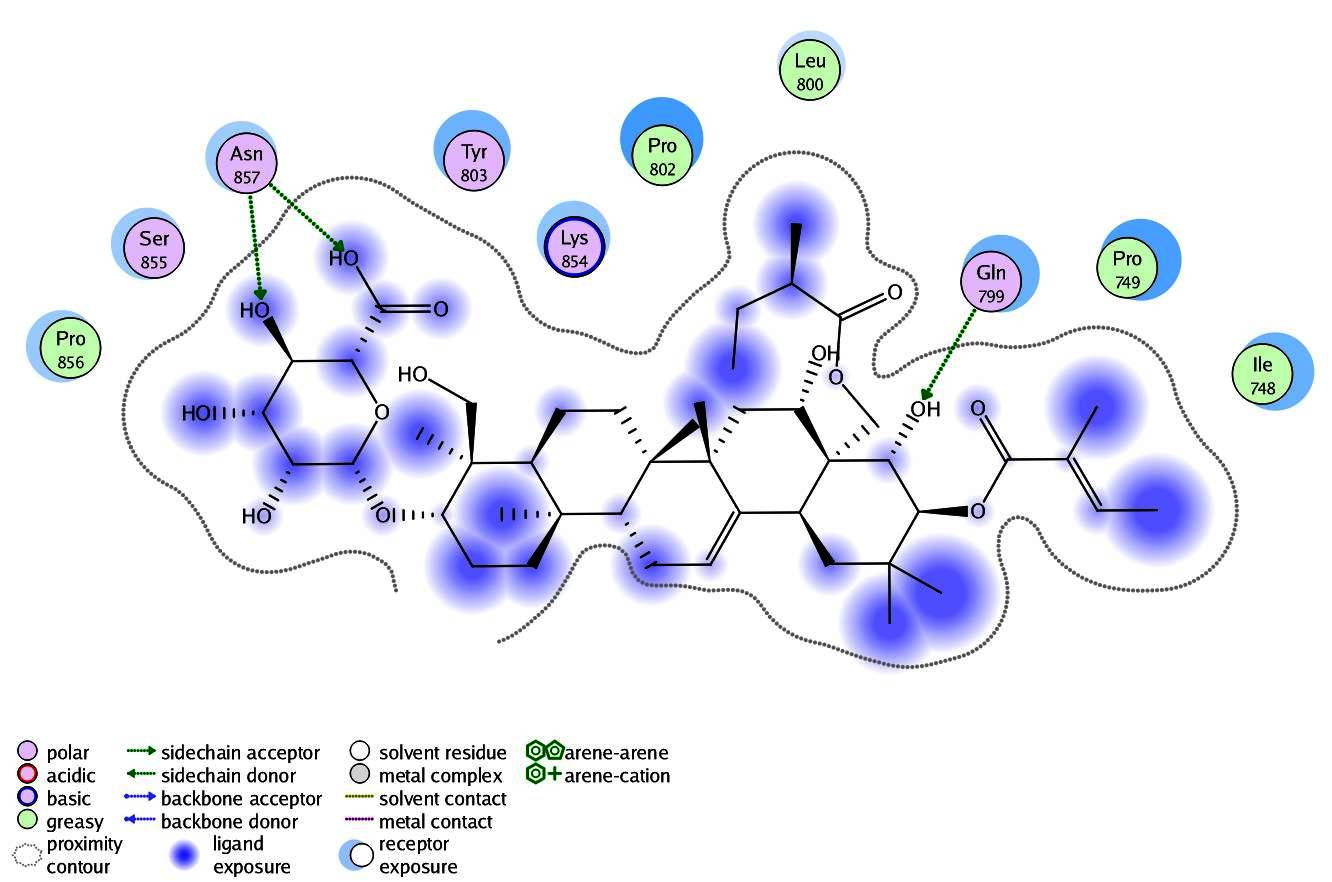  **Figure.8 Continued…..** |  |
| Gymnemic acid XII-HER2  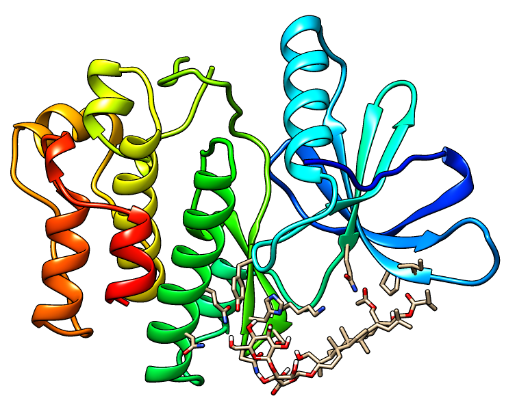 | Gymnemic acid XII-HER2  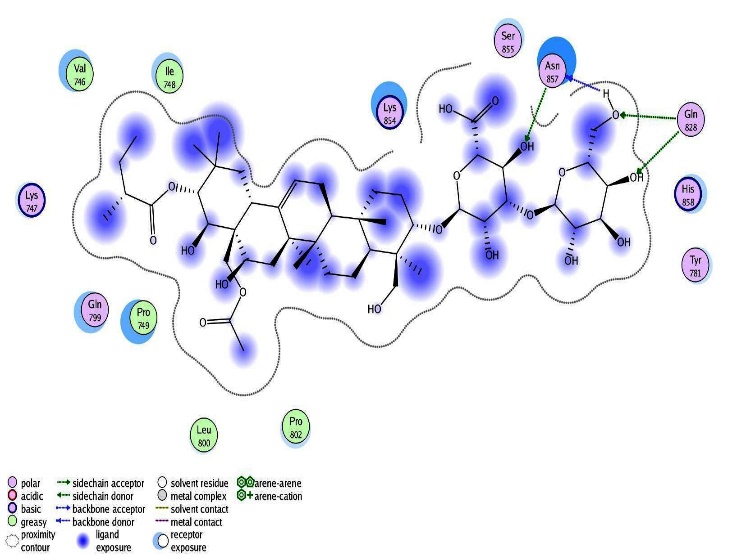 | | |
| Gymnemic acid XIII-HER2    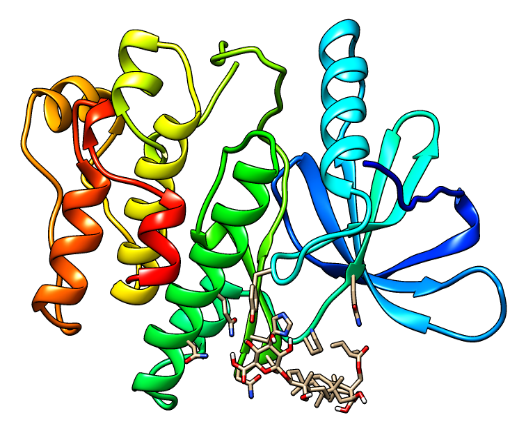 | Gymnemic acid XIII-HER2  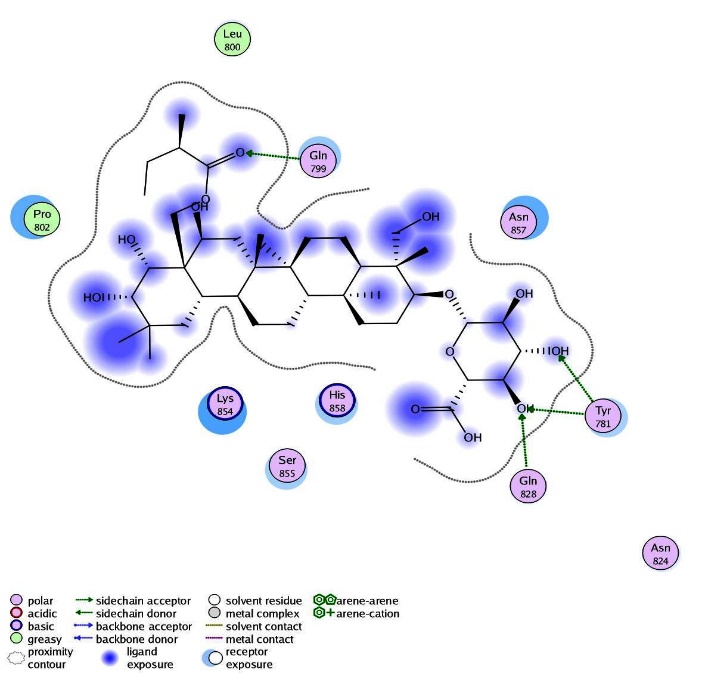 | | |

| Gymnemic acid XIV-HER2  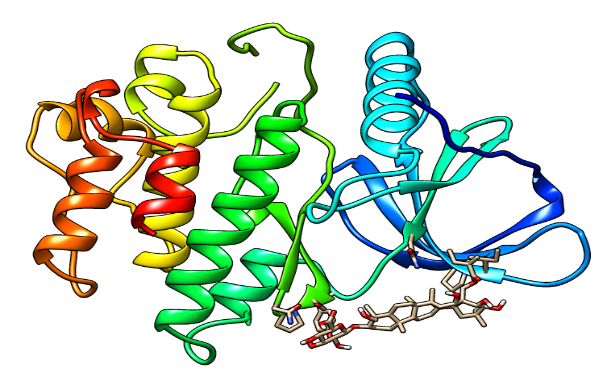 | Gymnemic acid XIV-HER2  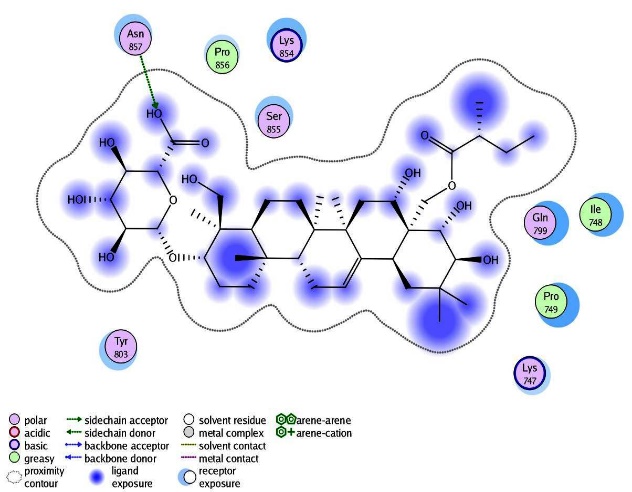 |
| --- | --- |
